# Contributions to the *Andrena* (Hymenoptera, Andrenidae) Fauna of the Mediterranean Region of Türkiye

**DOI:** 10.3897/BDJ.14.e182770

**Published:** 2026-03-24

**Authors:** Kerem Kaleli, Fatih Dikmen, Thomas James Wood, Çiğdem Özenirler, Ahmet Murat Aytekin

**Affiliations:** 1 Istanbul University, Institute of Graduate Studies in Sciences, Istanbul, Türkiye Istanbul University, Institute of Graduate Studies in Sciences Istanbul Türkiye; 2 Istanbul University, Faculty of Science, Department of Biology, Istanbul, Türkiye Istanbul University, Faculty of Science, Department of Biology Istanbul Türkiye; 3 Naturalis Biodiversity Center, Darwinweg 2, 2333 CR, Leiden, Netherlands Naturalis Biodiversity Center, Darwinweg 2, 2333 CR Leiden Netherlands; 4 Applied Biological Sciences Research Laboratory (ABS-Lab), Department of Biology, Faculty of Science, Hacettepe University, Ankara, Türkiye Applied Biological Sciences Research Laboratory (ABS-Lab), Department of Biology, Faculty of Science, Hacettepe University Ankara Türkiye

**Keywords:** *
Andrena
*, bee diversity, Mediterranean Region, pollinator-plant interactions, new records, taxonomy

## Abstract

**Background:**

*Andrena* is one of the most diverse bee genera in the Holarctic Region, yet its species diversity and distribution in Türkiye remain poorly documented. This study provides an updated faunistic assessment of the genus in the Mediterranean Region of southern Türkiye, based on extensive field sampling in 12 provinces between 2008 and 2009 and a comprehensive synthesis of existing records. Specimens were identified using the latest taxonomic revisions and provincial distributions were compiled to produce a contemporary regional baseline.

**New information:**

A total of 81 species were documented, belonging to 26 subgenera, including three endemic Turkish taxa (A. hungarica ssp. macroura, *A. purpureomicans* and *A. cinnamonea*), as well as *A. wolfi*, which was recorded in the country for the first time. Floral visitation data were also incorporated into the study. Furthermore, the species distribution was summarised with regional maps. The photos of morphological characters of regional and Turkish new records were also taken and included to the study. Together, these results provide the most current and comprehensive overview of *Andrena* diversity in southern Türkiye, offering a critical foundation for future research into the taxonomy, biogeography, pollination ecology and conservation of Mediterranean bee communities.

## Introduction

The order Hymenoptera, which contains over 150,000 identified species, is considered the fourth largest order within the class Insecta (Michener 2007, Mayhew 2007, Huber 2009). However, some studies suggest that this order may harbour a much greater number of species than previously thought, potentially making it the most species-diverse order (Forbes et al. 2018). Within this order, the group Anthophila, which belongs to the Apoidea superfamily, constitute the 'true bees', with approximately 21,000 known species (Gullan and Cranston 2010, Potts et al. 2016, Ascher and Pickering 2025). As bees are considered as being the primary pollinators for nearly 300,000 species of flowering plants (Angiospermae), this ecological service is indispensable for both the continuity of natural ecosystems and for global agricultural production (Ollerton et al. 2011, Güler 2011). Due to this multifaceted ecological and economic importance, bees continue to be one of the focal points for biodiversity and conservation studies (Michez et al. 2019).

It is estimated that bees (Anthophila) in Türkiye are represented by approximately 1,750 and possibly as many as 2,000 species with more than 75 genera (Özbek 2003, Hazır 2010, Dikmen 2018, Lhomme et al. 2020, Ascher and Pickering 2025). Although this figure shows how important a biodiversity centre Türkiye is for bees, our knowledge regarding the current status, precise distribution areas and population dynamics of these species is still incomplete nationwide (Güler 2011, Hazır et al. 2014, Dikmen 2018). One of the most common genus of bees in Türkiye is *Andrena* Fabricius, 1775 (Apoidea, Andrenidae) with 392 species reported (Wood 2023a, Wood 2024, Wood 2025, TJW unpublished data). This genus is the second richest globally, after *Lasioglossum* Curtis, 1833 (Apoidea, Halictidae), with around 1,738 species following recent revisions (Pisanty et al. 2022, Wood and Monfared 2022, Wood 2023a, Wood et al. 2024, Wood 2025, Ascher and Pickering 2025). However, this total is constantly growing due to ongoing active taxonomic revisions. This remarkable diversity is concentrated in the temperate and arid zones of the Holarctic Region, particularly within the Mediterranean Basin. Türkiye’s unique position as a bridge between continents, coupled with its varied topography and climate, makes it a significant hotspot for *Andrena* diversity (Hazır 2010, Wood 2023a). While pioneering researchers, such as Warncke (1965), Warncke (1967), Warncke (1975) and Özbek (1976), conducted foundational research that revealed the diversity of the genus in the country and established a strong baseline, comprehensive faunistic studies on the *Andrena* of Türkiye have remained limited since then (Scheuchl and Hazır 2012, Hazır et al. 2012, Hazır et al. 2014, Wood 2023a). Recent taxonomic interest in the region has revitalised research and the known diversity is once again expanding. A steady stream of studies is adding new species and country records to Turkish fauna, indicating that the actual number of species is likely to be much higher than previously reported (Wood 2021a, Pisanty et al. 2022, Wood 2023a, Wood 2024, Wood 2025).

Given the importance of these taxa and the existing knowledge gaps, this study aims to: (1) provide an up-to-date checklist for the region; (2) present preliminary distributional data for the species and (3) report new records and endemic species for the region, focusing on *Andrena* species in the Mediterranean Region of Türkiye, an area renowned for its rich biodiversity. Ultimately, we hope that this work will contribute to future research on the taxonomy, ecology and conservation on the genus *Andrena* of Türkiye.

## Materials and methods

### Field studies

This study was conducted within the Mediterranean Region of southern Türkiye. The study area encompassed twelve provinces representing the core distribution of the Mediterranean Basin in the country. Amongst these, eight provinces are entirely located within the Mediterranean Region (Adana, Antalya, Burdur, Hatay, Isparta, Mersin, Muğla and Osmaniye), whereas four provinces are only partly situated within the region (Denizli, Karaman, Kahramanmaraş and Niğde).

Field studies were conducted during the spring and summer seasons of 2008 and 2009. As the survey area covered more than 100,000 km², an opportunistic sampling protocol was employed to maximise the number of specimens collected. All bees were captured using hand nets and aspirators and floral resources visited by them were either recorded in the field or collected for subsequent identification. Bees were examined and photographed by Zeiss Discovery V8 stereoscopic microscope. Identification of the bee specimens was made by T. Wood. The species and their authorities are listed below in alphabetical order within subgenera (Table 1). The distribution of the species was compiled, based on a literature review and listed in alphabetical order. Identification of plant specimens were made by D. Töre according to Flora of Turkey and East Aegean Islands (Davis 1965, Davis 1988, Güner et al. 2000).

### Spatial data visualisation

**Figure 1.**
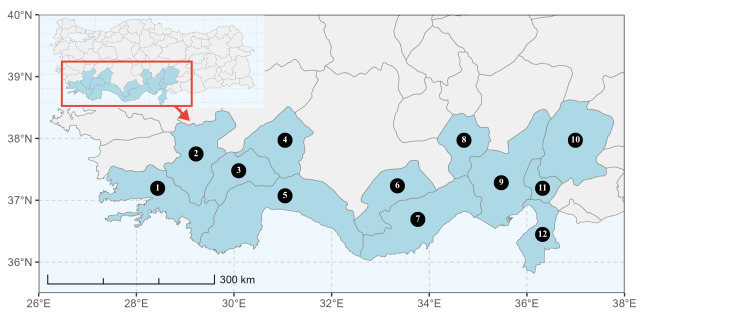
Study area in the Mediterranean Region of Türkiye, showing sampled provinces in blue. **1** Muğla; **2** Denizli; **3** Burdur; **4** Isparta; **5** Antalya; **6** Karaman; **7** Mersin; **8** Niğde; **9** Adana; **10** Kahramanmaraş; **11** Osmaniye; **12** Hatay.

The map of Türkiye (Fig. 1) displaying the Mediterranean Region and the sampled provinces was generated in R Studio (version 2025.05.1+513 or latest available; Posit team (2025)). The spatial extent was restricted to 27–37°E longitude and 35.5–38.5°N latitude to highlight the study area. Provincial boundaries were retrieved from publicly available shapefiles and the provinces were coloured according to the collected specimens. This visualisation was produced to facilitate geographic representation of the study area and to contextualise the distribution of the recorded *Andrena* species.

Specimen records were categorised into three data sources: literature records (L), collection-based records (C) and combined literature plus collection records (LC). For analytical purposes, both *L* and *LC* were treated as literature-based records, whereas species represented solely by *C* (i.e. present in the collection, but absent from the literature) were considered as new records (Table 2). Provinces were subsequently mapped according to these record categories, enabling comparison between previously known and newly-documented distributions. Taxonomic updates followed the most recent revisions of the *Andrena* fauna of the Palaearctic Region (see previous citations).

## Data resources

The data underpinning the analysis reported in this paper are deposited at GBIF, the Global Biodiversity Information Facility, https://ipt.pensoft.net/resource?r=andrena_med_tr

## Taxon treatments

### Andrena (Ulandrena) abbreviata

Dours, 1873

2765BD6B-05E4-5452-B27A-CA45626642CC

#### Materials

- **Type status:** Other material. **Occurrence:** recordedBy: Fatih Dikmen; individualCount: 1; sex: female; occurrenceID: 74954092-D803-5024-8830-2C6F9B0DCC0D; **Location:** countryCode: TR; stateProvince: Antalya; county: Akseki; locality: Akseki road; verbatimElevation: 1262 m; verbatimLatitude: 37.118; verbatimLongitude: 31.778; **Identification:** identifiedBy: Thomas J. Wood; **Event:** eventDate: 09-06-2009; **Record Level:** collectionCode: ZMUI
- **Type status:** Other material. **Occurrence:** recordedBy: Fatih Dikmen; individualCount: 1; sex: female; occurrenceID: B1FB5C84-10C4-5489-9D70-5D525250B7E8; **Location:** countryCode: TR; stateProvince: Mersin; county: Mut; locality: Güzlek Plateau; verbatimElevation: 1414 m; verbatimLatitude: 36.654; verbatimLongitude: 33.108; **Identification:** identifiedBy: Thomas J. Wood; **Event:** eventDate: 04-06-2009; **Record Level:** collectionCode: ZMUI
- **Type status:** Other material. **Occurrence:** recordedBy: Fatih Dikmen; individualCount: 2; sex: female; occurrenceID: F45DF9C8-8EA7-5C5F-ACD6-1A2FB8106403; **Location:** countryCode: TR; stateProvince: Afyon; county: Dazkırı; locality: Örtülü; verbatimElevation: 1192 m; verbatimLatitude: 37.899; verbatimLongitude: 29.754; **Identification:** identifiedBy: Thomas J. Wood; **Event:** eventDate: 12-06-2009; **Record Level:** collectionCode: ZMUI

#### Distribution

Albania, Bulgaria, Georgia, Greece, Iran, North Macedonia, Israel and the West Bank, Jordan, Lebanon, Syria and Türkiye (Wood 2024).

##### Distribution in Türkiye

**Figure 2.**
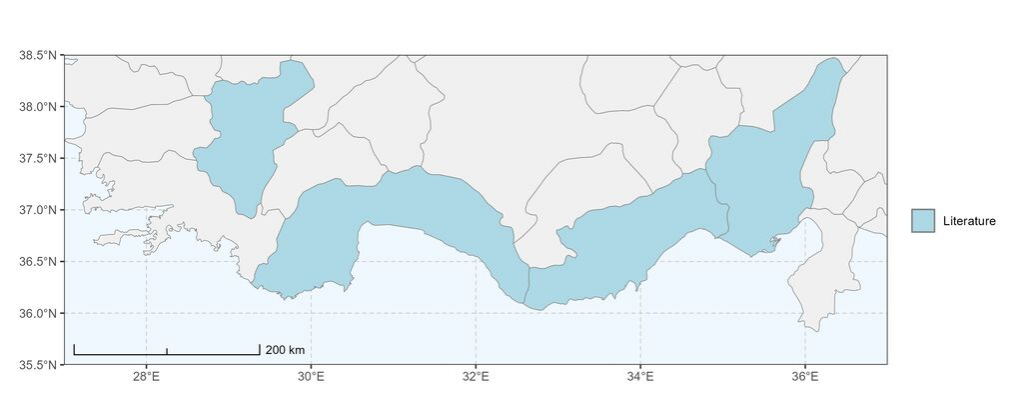
Distribution of *A. abbreviata* in the Mediterranean Region of Türkiye.

Antalya, Mersin (Warncke 1974); Adana, Adıyaman, Ağrı, Denizli, Erzurum, Eskişehir, Gaziantep, Konya, Kütahya, Malatya, Van (Wood 2024); Afyon, Antalya, Mersin (this study) (Fig. 2).

#### Flower records

**Brassicaceae**: *Brassica napus* L. (Khodarahmi Ghahnavieh and Monfared 2019); **Asteraceae**: *Anthemis pseudocotula* Boiss., Cota coelopoda var. coelopoda (Boiss.) Boiss., Anthemis coelopoda var. bourgaei Boiss.

### Andrena (Notandrena) aerinifrons

Dours, 1873

DE337F0A-F08C-5124-BDCB-679EBDE983E6

#### Materials

- **Type status:** Other material. **Occurrence:** recordedBy: Fatih Dikmen; individualCount: 1; sex: female; occurrenceID: 12120632-01B3-59AA-9BDB-A6DA373843D5; **Location:** countryCode: TR; stateProvince: Hatay; locality: Belen Pass; verbatimElevation: 274 m; verbatimLatitude: 36.46; verbatimLongitude: 36.279; **Identification:** identifiedBy: Thomas J. Wood; **Event:** eventDate: 21-03-2009; **Record Level:** collectionCode: ZMUI
- **Type status:** Other material. **Occurrence:** recordedBy: Fatih Dikmen; individualCount: 1; sex: male; occurrenceID: B0CDCEC4-9B84-5B1E-AF7C-969D05FB7B00; **Location:** countryCode: TR; stateProvince: Hatay; locality: Belen Pass; verbatimElevation: 274 m; verbatimLatitude: 36.46; verbatimLongitude: 36.279; **Identification:** identifiedBy: Thomas J. Wood; **Event:** eventDate: 21-03-2009; **Record Level:** collectionCode: ZMUI
- **Type status:** Other material. **Occurrence:** recordedBy: Fatih Dikmen; individualCount: 1; sex: male; occurrenceID: 9CC1D763-380C-55CE-8A6A-E0846D7435F1; **Location:** countryCode: TR; stateProvince: Hatay; county: Antakya; locality: Reyhanlı; verbatimElevation: 85 m; verbatimLatitude: 36.253; verbatimLongitude: 36.52; **Identification:** identifiedBy: Thomas J. Wood; **Event:** eventDate: 21-03-2009; **Record Level:** collectionCode: ZMUI
- **Type status:** Other material. **Occurrence:** recordedBy: Fatih Dikmen; individualCount: 1; sex: male; occurrenceID: 1E24B2E3-2320-5C0A-9EAE-113F8BF02D53; **Location:** countryCode: TR; stateProvince: Hatay; county: Kırıkhan; locality: Reyhanlı; verbatimElevation: 85 m; verbatimLatitude: 36.509; verbatimLongitude: 36.418; **Identification:** identifiedBy: Thomas J. Wood; **Event:** eventDate: 21-03-2009; **Record Level:** collectionCode: ZMUI

#### Distribution

Algeria, Egypt, Iran, Iraq, Israel and the West Bank, Italy, Jordan, Libya, Morocco, Portugal, Spain, Syria, Tunisia and Türkiye (Gusenleitner and Schwarz 2002, Wood and Monfared 2022).

##### Distribution in Türkiye

**Figure 3.**
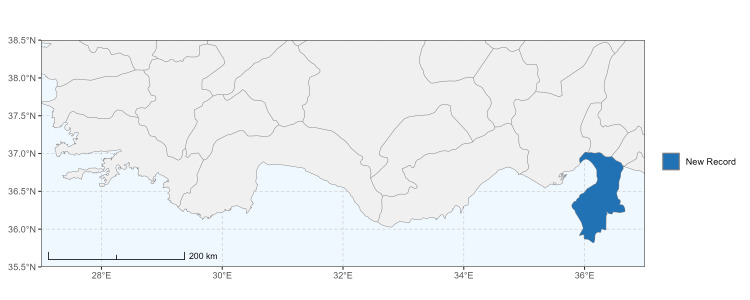
Distribution of *A. aerinifrons* in the Mediterranean Region of Türkiye.

**Figure 4.**
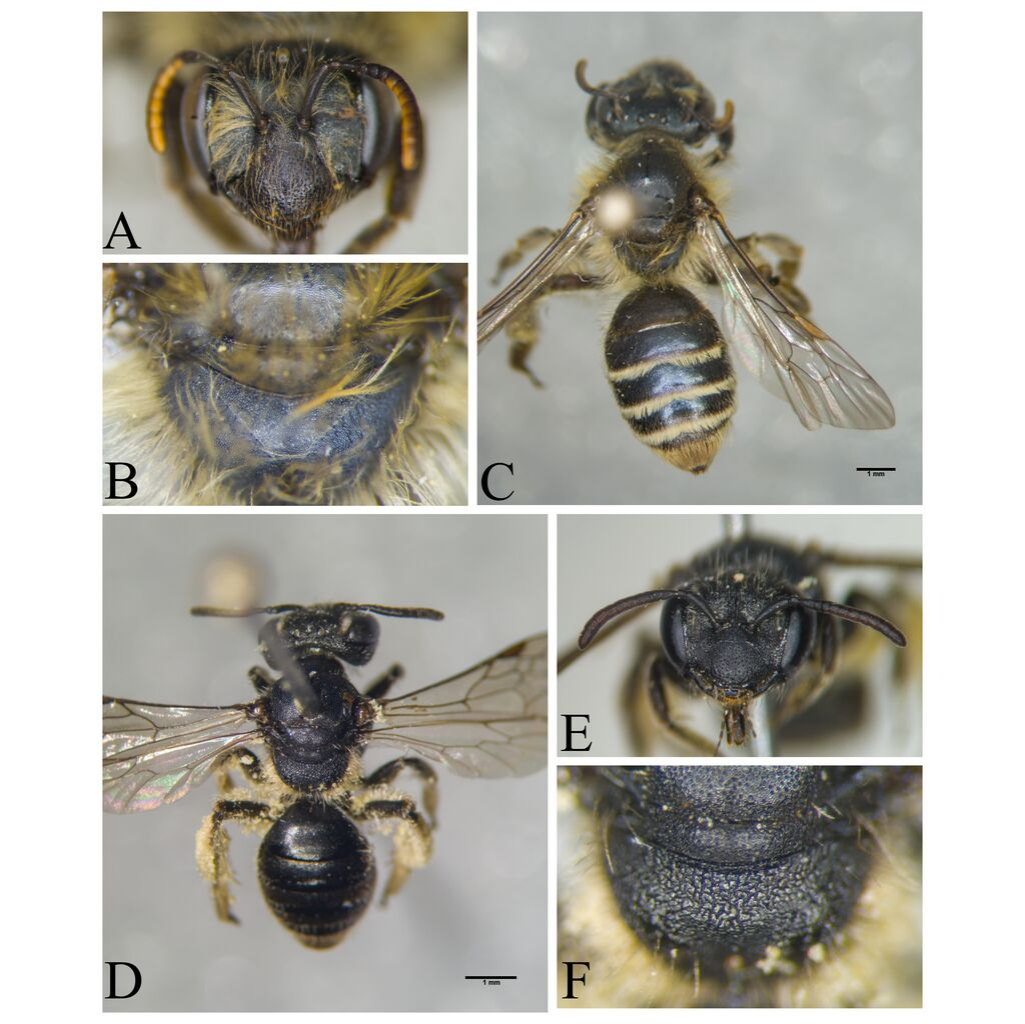
*Andrena aerinifrons*, female. Head, frontal view (A); propodeum, dorsal view (B); Habitus, dorsal view (C). *Andrena alfkenella*, female. Habitus, dorsal view (D), head, frontal view (E), propodeum, dorsal view (F).

Şanlıurfa (Warncke 1974); Kilis (Schönitzer 1997); Hatay (this study) (Fig. 3and Fig. 4A-C).

#### Flower records

**Brassicaceae**: *Eruca vesicaria* (L.) Cav., *Ochthodium aegyptiacum* (L.) DC., *Sisymbrium* L.

### Andrena (Micrandrena) alfkenella

Perkins, 1914

A94324E1-1ACD-5248-BE14-1B87107F090E

#### Materials

- **Type status:** Other material. **Occurrence:** recordedBy: Fatih Dikmen; individualCount: 2; sex: female; occurrenceID: 41B3D84E-42F3-5E05-A9D4-9E2320FE426C; **Location:** countryCode: TR; stateProvince: Hatay; locality: Belen-Radar road; verbatimElevation: 1124 m; verbatimLatitude: 36.519; verbatimLongitude: 36.243; **Identification:** identifiedBy: Thomas J. Wood; **Event:** eventDate: 30-06-2009; **Record Level:** collectionCode: ZMUI

#### Distribution

Albania, Austria, Azerbaijan, Belarus, Belgium, Bosnia and Herzegovina, Bulgaria, Croatia, Czechia, Denmark, France, Georgia, Germany, Great Britain, Greece, Hungary, Iran, Italy, Latvia, Lithuania, Luxembourg, Moldova, Morocco, Netherlands, North Macedonia, Poland, Portugal, Romania, Russia, Serbia, Slovakia, Slovenia, Spain, Sweden, Switzerland, Türkiye and Ukraine (Gusenleitner and Schwarz 2002, Wood et al. 2020, Wood 2024, Ascher and Pickering 2025).

##### Distribution in Türkiye

**Figure 5.**
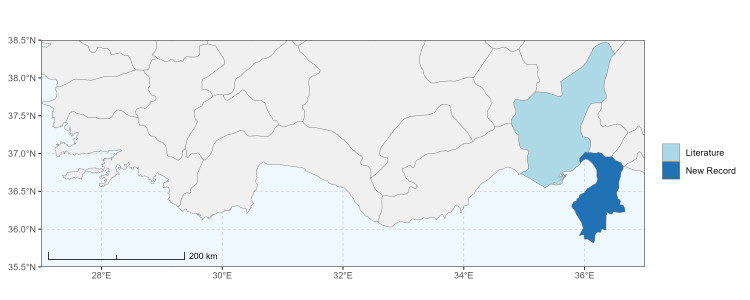
Distribution of *A. alfkenella* in the Mediterranean Region of Türkiye.

Erzurum, Diyarbakır (Warncke 1974); Adana (Dardón et al. 2014); Hatay (this study) (Fig. 5 and Fig. 4D-F).

#### Flower records

**Apiaceae**: *Daucus carota* L., *Heracleum sphondylium* L. (Balfour et al. 2022); **Apiaceae**: *Aegopodium podagraria* L., *Carum carvi* L., *Chaerophyllum bulbosum* L., *Daucus carota* L., *Ferula tingitana* L., *Heracleum sphondylium* L., *Pimpinella saxifraga* L., *Torilis arvensis* (Huds.) Link **Asteraceae**: *Achillea millefolium* L., *Erigeron annuus* (L.) Desf., *Matricaria chamomilla* L., *Tripleurospermum maritimum* (L.) W.D.J.Koch **Brassicaceae**: *Hirschfeldia incana* (L.) Lagr.-Foss., *Rorippa amphibia* (L.) Besser, *Rorippa sylvestris* (L.) Besser, *Sisymbrium loeselii* L. **Caryophyllaceae**: *Stellaria graminea* L. **Crassulaceae**: *Sedum acre* L. **Fabaceae**: *Lotus corniculatus* L., *Medicago lupulina* L. **Geraniaceae**: *Geranium pyrenaicum* Burm.f. **Lamiaceae**: *Thymus* L. **Ranunculaceae**: *Ranunculus paludosus* Poir., *Ranunculus polyanthemos* L. **Rosaceae**: *Potentilla reptans* L. **Rubiaceae**: *Galium lucidum* All. (Lanuza et al. 2025); **Rosaceae**: Rubus canescens var. glabratus (Godr.) Davis & Meikle.

### Andrena (Micrandrena) alfkenelloides

Warncke, 1965

2F5A0BE7-46C1-51C0-BA03-C1C62B58BF9C

#### Materials

- **Type status:** Other material. **Occurrence:** recordedBy: Fatih Dikmen; individualCount: 1; sex: female; occurrenceID: DDB26D2C-B27E-5419-A2DC-D9CA48057737; **Location:** countryCode: TR; stateProvince: Osmaniye; locality: Karatepe National Park; verbatimElevation: 325 m; verbatimLatitude: 37.26; verbatimLongitude: 36.219; **Identification:** identifiedBy: Thomas J. Wood; **Event:** eventDate: 20-03-2009; **Record Level:** collectionCode: ZMUI

#### Distribution

Bulgaria, Cyprus, Greece, Israel and the West Bank, Iran, Jordan, Lebanon, Syria and Türkiye (Gusenleitner and Schwarz 2002, Wood et al. 2020).

##### Distribution in Türkiye

**Figure 6.**
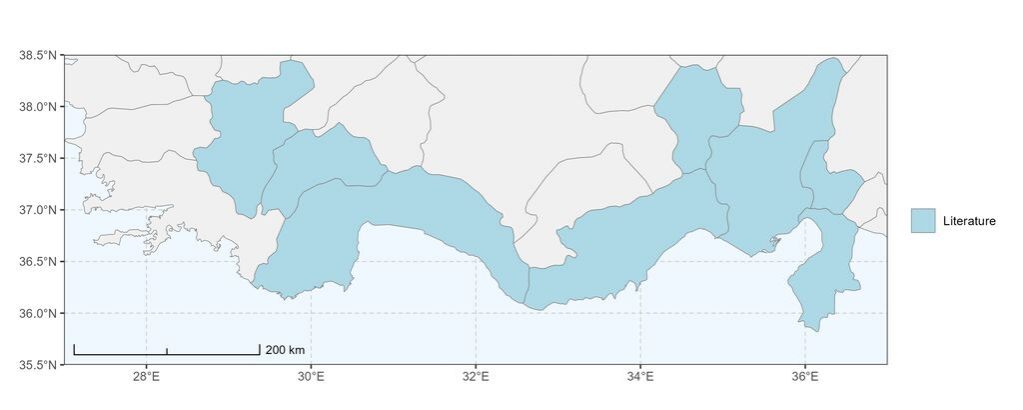
Distribution of *A. alfkenelloides* in the Mediterranean Region of Türkiye.

Ankara, Manisa, Nevşehir (Ürgüp), Denizli, Antalya, Mersin, Niğde, Tunceli, Adana, Osmaniye, Hatay (Warncke 1974); Aydın, Burdur, Mersin (Hazır et al. 2014); Osmaniye (this study) (Fig. 6).

#### Flower records

**Asteraceae**: *Anthemis chia* L., *Crepis commutata* (Spreng.) Greuter **Apiaceae**: *Pimpinella cretica* Poir., *Pimpinella peregrina* L. (Lanuza et al. 2025); **Asteraceae**: *Senecio vernalis* Waldst. & Kit.

### Andrena (Micrandrena) alutacea

Stöckhert, 1942

49CD4FCB-22EB-524B-882B-BB23D55472C4

#### Materials

- **Type status:** Other material. **Occurrence:** recordedBy: Fatih Dikmen; individualCount: 2; sex: female; occurrenceID: 2750D662-8609-55D3-A4CD-FC19ECCEAE92; **Location:** countryCode: TR; stateProvince: Mersin; county: Mut; locality: Güzlek Plateau; verbatimElevation: 1414 m; verbatimLatitude: 36.354; verbatimLongitude: 33.107; **Identification:** identifiedBy: Thomas J. Wood; **Event:** eventDate: 04-06-2009; **Record Level:** collectionCode: ZMUI

#### Distribution

Austria, Azerbaijan, Bulgaria, Crimea, Croatia, Georgia, Germany, Greece, Iran, Italy, Latvia, Montenegro, North Macedonia, Poland, Romania, Russia, Switzerland, Turkmenistan, Türkiye and Ukraine (Schmid-Egger 2005, Proshchalykin et al. 2017, Wood and Monfared 2022).

##### Distribution in Türkiye

**Figure 7.**
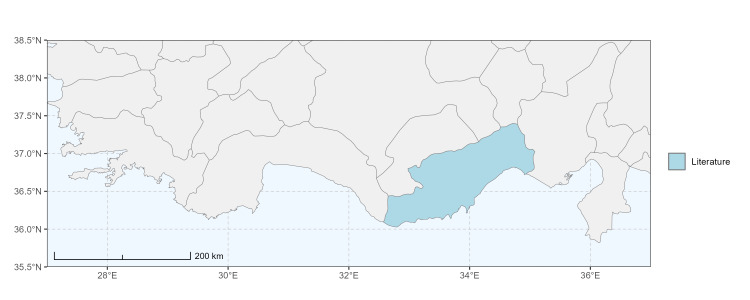
Distribution of *A. alutacea* in the Mediterranean Region of Türkiye.

Ağrı, Amasya, Erzurum, Hakkari, Kars, Konya, Mersin, Muş, Nevşehir, Sivas (Schmid-Egger 2005); Ankara, Aydın, Çankırı, Konya, Kütahya (Hazır et al. 2014); Mersin (this study) (Fig. 7).

#### Flower records

**Apiaceae**: *Prangos uechtritzii* Boiss. & Hausskn

### Andrena (Chlorandrena) astica

Warncke, 1967

02052E82-27C1-5169-AA88-D5A4F4EF11CE

#### Materials

- **Type status:** Other material. **Occurrence:** recordedBy: Fatih Dikmen; individualCount: 1; sex: female; occurrenceID: 4C57582C-1356-5330-9FBA-1621AEBCAB7B; **Location:** countryCode: TR; stateProvince: Adana; locality: Çukurova University; verbatimElevation: 83 m; verbatimLatitude: 37.049; verbatimLongitude: 35.357; **Identification:** identifiedBy: Thomas J. Wood; **Event:** eventDate: 20-03-2009; **Record Level:** collectionCode: ZMUI
- **Type status:** Other material. **Occurrence:** recordedBy: Fatih Dikmen; individualCount: 2; sex: male; occurrenceID: 30EC40D8-F5BC-5DBE-B1A2-C77C71CB53E4; **Location:** countryCode: TR; stateProvince: Hatay; locality: Belen Pass; verbatimElevation: 274 m; verbatimLatitude: 36.46; verbatimLongitude: 36.279; **Identification:** identifiedBy: Thomas J. Wood; **Event:** eventDate: 21-03-2009; **Record Level:** collectionCode: ZMUI

#### Distribution

Cyprus, Georgia, Greece, Iran, Iraq, Israel and West Bank, Lebanon and Türkiye (Schwenninger 2015, Wood 2021b, Wood and Monfared 2022, Wood et al. 2024).

##### Distribution in Türkiye

**Figure 8.**
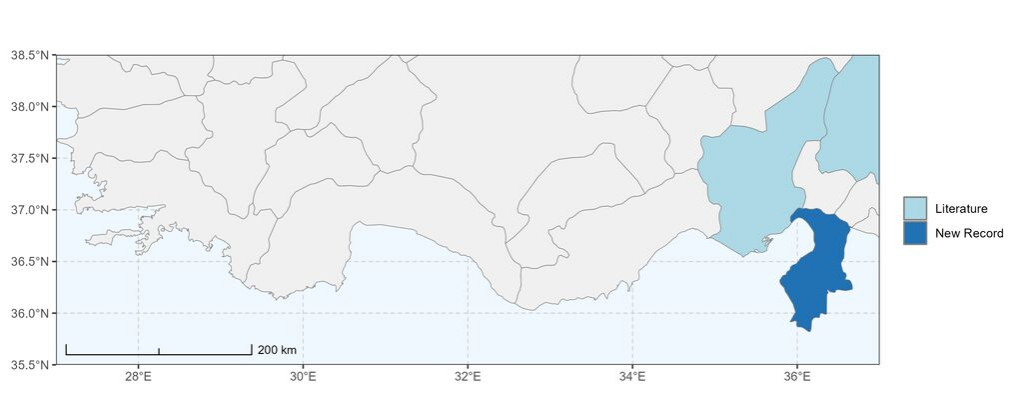
Distribution of *A. astica* in the Mediterranean Region of Türkiye.

Balıkesir, Adana (Warncke 1974); Maraş (Hazır 2010, Hazır et al. 2014); Adana, Hatay (this study) (Fig. 8).

#### Flower records

**Asteraceae**: *Crepis sancta* (L.) Bornm. **Brassicaceae**: *Sisymbrium* L.

### Andrena (Euandrena) bicolor

Fabricius, 1775

A30A27ED-922C-55F3-A095-5697FC8C564F

#### Materials

- **Type status:** Other material. **Occurrence:** recordedBy: Fatih Dikmen; individualCount: 1; sex: female; occurrenceID: 326EA524-F698-5679-A36F-377F663D0313; **Location:** countryCode: TR; stateProvince: Hatay; locality: Yayladağ; verbatimElevation: 423 m; verbatimLatitude: 35.908; verbatimLongitude: 36.078; **Identification:** identifiedBy: Thomas J. Wood; **Event:** eventDate: 22-03-2009; **Record Level:** collectionCode: ZMUI

#### Distribution

Algeria, Albania, Austria, Belgium, Bosnia and Herzegovina, Bulgaria, Croatia, Czechia, Denmark, Estonia, Finland, France, Germany, Great Britain, Greece, Hungary, Iran, Ireland, Italy, Latvia, Lithuania, Luxembourg, Morocco, Netherlands, Norway, Poland, Portugal, Romania, Russia, Serbia, Slovakia, Slovenia, Spain, Sweden, Tunisia, Türkiye and Ukraine (Gusenleitner and Schwarz 2002, Osytshnjuk et al. 2008, Wood and Monfared 2022).

##### Distribution in Türkiye

**Figure 9.**
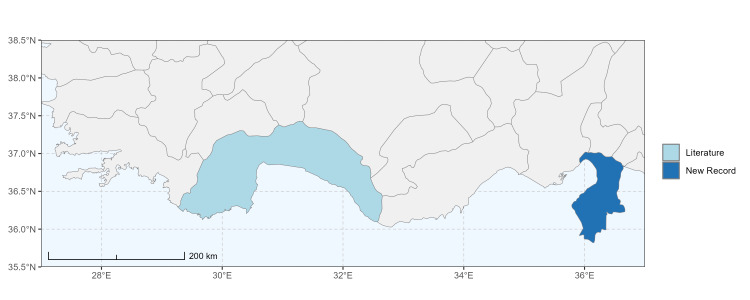
Distribution of *A. bicolor* in the Mediterranean Region of Türkiye.

Amasya, Ankara, Antalya, Ardahan, Balıkesir, Bursa, Diyarbakır, Erzurum, Kayseri, Konya, Nevşehir (Ürgüp), Tekirdağ (Warncke 1974); Ankara, Aydın, Bursa, Kırklareli, Sivas (Hazır 2010, Hazır et al. 2014); Hatay (this study) (Fig. 9).

#### Flower records

**Amaryllidaceae**: *Narcissus* L. **Apiaceae**: *Anthriscus sylvestris* (L.) Hoffm., *Daucus carota* L., *Daucus* L., *Erodium* L’Hér., *Heracleum sphondylium* L., *Oenanthe crocata* L. **Araliaceae**: *Hedera* L. **Asparagaceae**: *Hyacinthoides non-scripta* (L.) Chouard ex Rothm. **Asteraceae**: *Anthemis* L., *Bellis perennis* L., *Centaurea nigra* L., *Cirsium arvense* (L.) Scop., *Cirsium* Mill., *Crepis capillaris* (L.) Wallr., *Crepis* L., *Hieracium* L., *Hypochaeris radicata* L., *Lapsana communis* L., *Leontodon* L., *Leucanthemum vulgare* Lam., *Matricaria chamomilla* L., *Picris hieracioides* L., *Picris* L., *Scorzoneroides autumnalis* (L.) Moench, *Senecio jacobaea* L., *Sonchus arvensis* L., *Sonchus* L., *Taraxacum* agg. F.H.Wigg., *Taraxacum officinale* agg. F.H.Wigg., *Taraxacum* F.H.Wigg., *Tripleurospermum inodorum* (L.) Sch.Bip. **Brassicaceae**: *Arabis* L., *Brassica napus* L., *Brassica* L., *Cakile maritima* Scop., *Capsella bursa-pastoris* (L.) Medik., *Sinapis alba* L., *Sinapis arvensis* L., *Sisymbrium officinale* (L.) Scop. **Campanulaceae**: *Campanula portenschlagiana* Schult., *Campanula rotundifolia* L., *Campanula* L., *Campanula trachelium* L. **Caprifoliaceae**: *Symphoricarpos albus* (L.) S.F.Blake, *Valerianella* Mill. **Caryophyllaceae**: *Stellaria* L., **Convolvulaceae**: *Convolvulus arvensis* L. **Cornaceae**: *Cornus alba* L. **Cucurbitaceae**: *Bryonia dioica* Bojer **Fabaceae**: *Lotus corniculatus* L., *Medicago lupulina* L., *Trifolium campestre* Schreb., *Trifolium hybridum* L., *Trifolium pratense* L., *Trifolium repens* L., *Trifolium* Tourn. ex L., *Ulex* L. **Geraniaceae**: *Geranium pyrenaicum* Burm.f., *Geranium robertianum* L., *Geranium* Tourn. ex L. **Lamiaceae**: *Lamium album* L., *Mentha aquatica* L. **Malvaceae**: *Tilia cordata* Mill., *Tilia* L. **Plantaginaceae**: *Veronica chamaedrys* L., *Veronica persica* Poir., *Veronica* L. **Polemoniaceae**: *Phlox* L. **Primulaceae**: *Primula elatior* (L.) Hill, *Primula* L., *Primula vulgaris* Huds. **Ranunculaceae**: *Clematis vitalba* L., *Ranunculus ficaria* L., *Ranunculus repens* L., *Ranunculus* L. **Rosaceae**: *Crataegus monogyna* Jacq., *Filipendula ulmaria* (L.) Maxim., *Fragaria* L., *Potentilla erecta* (L.) Raeusch., *Potentilla reptans* L., *Potentilla* L., *Potentilla sterilis* (L.) Garcke, *Rosa arvensis* Huds., *Rosa* L., *Rubus fruticosus* agg. L., *Rubus* L. **Rubiaceae**: *Sherardia arvensis* L. **Salicaceae**: *Salix* L. **Sapindaceae**: *Acer* L. **Viburnaceae**: *Viburnum* L. (Balfour et al. 2022); **Apiaceae**: *Falcaria vulgaris* Bernh., *Heracleum sphondylium* L., *Pimpinella saxifraga* L. **Asteraceae**: *Hieracium* L., *Leontodon hispidus* L., *Leontodon saxatilis* Lam., *Taraxacum* F.H.Wigg., *Taraxacum officinale* F.H.Wigg. **Boraginaceae**: *Pentaglottis sempervirens* (L.) Tausch ex L.H.Bailey **Brassicaceae**: *Brassica napus* L. **Campanulaceae**: *Campanula bononiensis* L., *Campanula patula* L., *Campanula portenschlagiana* Schult., *Campanula rapunculoides* L., *Campanula rotundifolia* L. **Convolvulaceae**: *Convolvulus arvensis* L. **Cucurbitaceae**: *Bryonia cretica* L. **Fabaceae**: *Medicago lupulina* L., *Melilotus officinalis* (L.) Lam., *Vicia sepium* L. **Geraniaceae**: *Geranium* Tourn. ex L., *Geranium dissectum* L., *Geranium pyrenaicum* Burm.f., *Geranium sanguineum* L. **Lamiaceae**: *Thymus pulegioides* L. **Linaceae**: *Linum austriacum* L., *Linum tenuifolium* L. **Malvaceae**: *Malva sylvestris* L. **Orobanchaceae**: *Orobanche gracilis* Sm. **Primulaceae**: *Primula veris* L. **Rosaceae**: *Rubus* L. (Lanuza et al. 2025); **Asteraceae**: *Senecio vernalis* Waldst. & Kit.

### Andrena (Plastandrena) bimaculata

(Kirby, 1802)

D1B66970-1B31-5EA8-A982-E081A048C367

#### Materials

- **Type status:** Other material. **Occurrence:** recordedBy: Fatih Dikmen; individualCount: 2; sex: female; occurrenceID: 85EE365A-208A-5645-88F0-CD67130CB702; **Location:** countryCode: TR; stateProvince: Hatay; locality: between Yayladağ and Samandağ; verbatimElevation: 520 m; verbatimLatitude: 35.949; verbatimLongitude: 36.052; **Identification:** identifiedBy: Thomas J. Wood; **Event:** eventDate: 22-03-2009; **Record Level:** collectionCode: ZMUI

#### Distribution

Algeria, Armenia, Austria, Azerbaijan, Belarus, Belgium, Bosnia and Herzegovina, Bulgaria, Croatia, Czechia, Denmark, Estonia, Finland, France, Georgia, Germany, Great Britain, Greece, Hungary, Iran, Ireland, Israel and the West Bank, Italy, Jordan, Latvia, Lebanon, Lithuania, Luxembourg, Morocco, Netherlands, Norway, Poland, Portugal, Romania, Russia, Serbia, Slovakia, Slovenia, Spain, Sweden, Switzerland, Syria, Tunisia, Türkiye and Ukraine (Gusenleitner and Schwarz 2002, Wood et al. 2024, Ascher and Pickering 2025).

##### Distribution in Türkiye

**Figure 10.**
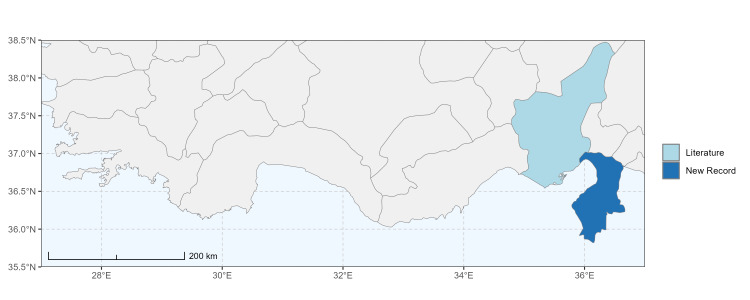
Distribution of *A. bimaculata* in the Mediterranean Region of Türkiye.

Adana, Balıkesir (Ayvalık), Malatya, Sakarya (Adapazarı) (Warncke 1974); Ankara, Aydın, Kastamonu, Kayseri (Hazır 2010, Hazır et al. 2014); Hatay (this study) (Fig. 10).

#### Flower records

**Amaryllidaceae**: *Allium* L. **Apiaceae**: *Heracleum sphondylium* L. **Asteraceae**: *Cirsium* Mill., *Senecio jacobaea* L. **Brassicaceae**: *Cakile maritima* Scop., *Sisymbrium* L. **Ericaceae**: *Erica* Tourn. ex L. **Fagaceae**: *Castanea sativa* Mill. **Polygonaceae**: *Rumex* L. **Rosaceae**: *Crataegus monogyna* Jacq., *Filipendula ulmaria* (L.) Maxim., *Fragaria* L., *Prunus* L., *Rubus fruticosus* agg. L., *Rubus* L. **Salicaceae**: *Salix* L., *Salix triandra* L. (Balfour et al. 2022); **Fabaceae**: *Lotus corniculatus* L., *Trifolium repens* L. **Rosaceae**: *Rosa* L. (Lanuza et al. 2025); **Asteraceae**: *Crepis sancta* (L.) Bornm.

### Andrena (Aenandrena) bisulcata

Morawitz, 1877

5C86315B-BB9F-5923-A9DB-6E34B218B5D0

#### Materials

- **Type status:** Other material. **Occurrence:** recordedBy: Fatih Dikmen; individualCount: 1; sex: male; occurrenceID: CD58061B-25A6-5F07-9788-DA2980F5E3B0; **Location:** countryCode: TR; stateProvince: Mersin; locality: Mersin-Adana highway; verbatimElevation: 189 m; verbatimLatitude: 36.937; verbatimLongitude: 34.799; **Identification:** identifiedBy: Thomas J. Wood; **Event:** eventDate: 22-05-2009; **Record Level:** collectionCode: ZMUI
- **Type status:** Other material. **Occurrence:** recordedBy: Fatih Dikmen; individualCount: 1; sex: male; occurrenceID: B96ED88C-8101-53F6-8784-0FA5E104FDA8; **Location:** countryCode: TR; stateProvince: Mersin; county: Mut; locality: Güzlek Plateau; verbatimElevation: 1414 m; verbatimLatitude: 36.654; verbatimLongitude: 33.107; **Identification:** identifiedBy: Thomas J. Wood; **Event:** eventDate: 04-06-2009; **Record Level:** collectionCode: ZMUI

#### Distribution

Austria, Azerbaijan, Bulgaria, Greece, Hungary, Israel and the West Bank, Italy, Iran, Moldova, Poland, Romania, Russia, Slovakia, Türkiye and Ukraine (Gusenleitner and Schwarz 2002, Wood et al. 2020, Wood and Monfared 2022).

##### Distribution in Türkiye

**Figure 11.**
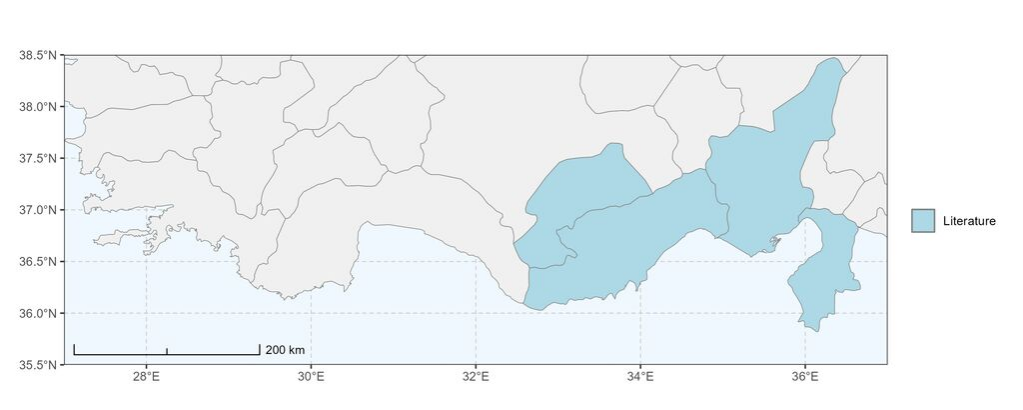
Distribution of *A. bisulcata* in the Mediterranean Region of Türkiye.

Adana (Ceyhan), Balıkesir (Ayvalık), Çanakkale, Diyarbakır, Edirne, Hatay (Antakya, İskenderun), Mersin (Tarsus), Samsun (Vezirköprü), Sakarya (Adapazarı), Tekirdağ, Urfa (Warncke 1974); Antep, Burdur, Çanakkale, Karaman, Manisa (Hazır 2010, Hazır et al. 2014); Mersin (this study) (Fig. 11).

#### Flower records

**Brassicaceae**: *Hirschfeldia incana* (L.) Lagr.-Foss.

### Andrena (Cryptandrena) brumanensis

Friese, 1899

AEC36BC0-60A5-5E05-994C-D61981D80E1F

#### Materials

- **Type status:** Other material. **Occurrence:** recordedBy: Fatih Dikmen; individualCount: 1; sex: male; occurrenceID: 4C269114-53C0-5E4C-A100-73EEC5D7F020; **Location:** countryCode: TR; stateProvince: Muğla; county: Marmaris; locality: Bördübet road; verbatimElevation: 195 m; verbatimLatitude: 36.857; verbatimLongitude: 28.126; **Identification:** identifiedBy: Thomas J. Wood; **Event:** eventDate: 04-04-2009; **Record Level:** collectionCode: ZMUI
- **Type status:** Other material. **Occurrence:** recordedBy: Fatih Dikmen; individualCount: 3; sex: female; occurrenceID: 39748768-1E27-5945-BC6B-DD9E16C9F19B; **Location:** countryCode: TR; stateProvince: Antalya; locality: Korkuteli-Finike road; verbatimElevation: 1346 m; verbatimLatitude: 36.949; verbatimLongitude: 30.131; **Identification:** identifiedBy: Thomas J. Wood; **Event:** eventDate: 10-06-2009; **Record Level:** collectionCode: ZMUI

#### Distribution

Albania, Bosnia and Herzegovina, Bulgaria, Croatia, Cyprus, France, Greece, Iran, Israel and the West Bank, Italy, Jordan, Lebanon, Monaco, Montenegro, Palestine, Slovenia, Syria and Türkiye (Gusenleitner and Schwarz 2002, Wood and Monfared 2022, Wood et al. 2024, Wood 2025).

##### Distribution in Türkiye

**Figure 12.**
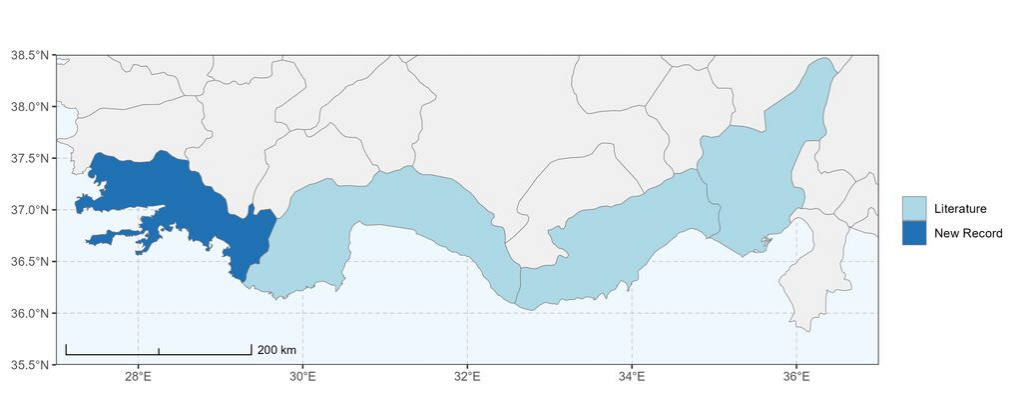
Distribution of *A. brumanensis* in the Mediterranean Region of Türkiye.

Adana, Ankara, Balıkesir (Ayvalık), Bursa, Çanakkale (Bigadiç), İstanbul (Kilyos), Konya, Mersin (Mut), Muş, Şanlıurfa (Birecik) (Warncke 1974); Erzurum (Oltu), Muş (Özbek 1976); Diyarbakır (Hazır 2010); Ankara, Antalya, Diyarbakır, Konya, Mersin, Urfa (Hazır et al. 2014); Antalya, Muğla (this study) (Fig. 12).

#### Flower records

**Caryophyllaceae**: *Silene colorata* Poir. **Plantaginaceae**: *Plantago lagopus* L., *Veronica barrelieri* H.Schott ex Roem. & Schult. (Lanuza et al. 2025); **Asteraceae**: *Crepis* L.

### Andrena (Micrandrena) cedricola

Wood, 2020

91D2A24F-2094-5446-97E9-6D31E7E4B90B

#### Materials

- **Type status:** Other material. **Occurrence:** recordedBy: Fatih Dikmen; individualCount: 2; sex: female; occurrenceID: F65FE683-2AAC-5258-977A-D555127C05AB; **Location:** countryCode: TR; stateProvince: Niğde; locality: Alihoca village; verbatimElevation: 1173 m; verbatimLatitude: 37.483; verbatimLongitude: 34.705; **Identification:** identifiedBy: Thomas J. Wood; **Event:** eventDate: 22-05-2008; **Record Level:** collectionCode: ZMUI
- **Type status:** Other material. **Occurrence:** recordedBy: Fatih Dikmen; individualCount: 1; sex: male; occurrenceID: D696C389-DFE5-579A-B4C5-2C9140B0BBF1; **Location:** countryCode: TR; stateProvince: Niğde; locality: Alihoca village; verbatimElevation: 1173 m; verbatimLatitude: 37.483; verbatimLongitude: 34.705; **Identification:** identifiedBy: Thomas J. Wood; **Event:** eventDate: 22-05-2008; **Record Level:** collectionCode: ZMUI

#### Distribution

Iraq, Israel and the West Bank, Lebanon, Syria and Türkiye (Pisanty et al. 2022, Wood et al. 2024).

##### Distribution in Türkiye

**Figure 13.**
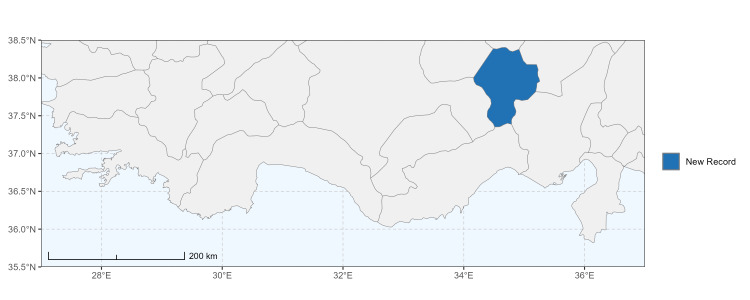
Distribution of *A. cedricola* in the Mediterranean Region of Türkiye.

**Figure 14.**
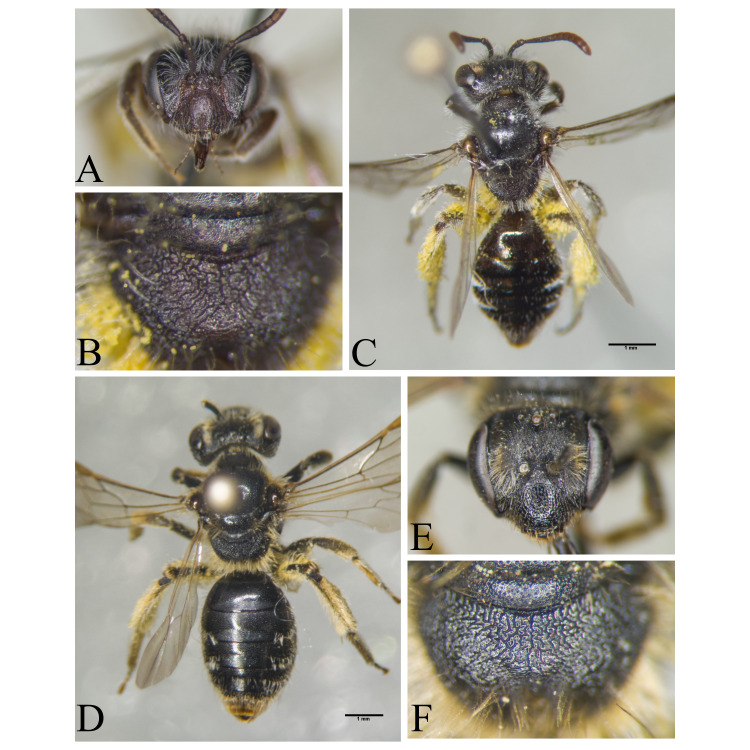
*Andrena cedricola*, female. Head, frontal view (A); propodeum, dorsal view (B); Habitus, dorsal view (C). *Andrena enslinella*, female. Habitus, dorsal view (D), head, frontal view (E), propodeum, dorsal view (F).

Hakkari (Pisanty et al. 2022); Niğde (this study) (Fig. 13 and Fig. 14A-C).

#### Flower records

**Brassicaceae**: *Brassica* L., *Peltaria angustifolia* DC. (Pisanty et al. 2022).

### Andrena (Chlorandrena) cinereophila

Warncke, 1965

FE84CA6A-86FF-593D-A205-72399867164F

#### Materials

- **Type status:** Other material. **Occurrence:** recordedBy: Fatih Dikmen; individualCount: 4; sex: female; occurrenceID: 8CEA5C11-BD49-5E57-9CF4-EE0513BB9D1A; **Location:** countryCode: TR; stateProvince: Adana; locality: Çukurova University, Balcalı Campus; verbatimElevation: 83 m; verbatimLatitude: 37.049; verbatimLongitude: 35.357; **Identification:** identifiedBy: Thomas J. Wood; **Event:** eventDate: 20-03-2009; **Record Level:** collectionCode: ZMUI
- **Type status:** Other material. **Occurrence:** recordedBy: Fatih Dikmen; individualCount: 4; sex: male; occurrenceID: A86228B4-3267-5B30-8D5C-37FBA84F2A94; **Location:** countryCode: TR; stateProvince: Adana; locality: Çukurova University, Balcalı Campus; verbatimElevation: 83 m; verbatimLatitude: 37.049; verbatimLongitude: 35.357; **Identification:** identifiedBy: Thomas J. Wood; **Event:** eventDate: 20-03-2009; **Record Level:** collectionCode: ZMUI
- **Type status:** Other material. **Occurrence:** recordedBy: Fatih Dikmen; individualCount: 1; sex: female; occurrenceID: 475CF6B2-FE80-52A5-A41E-121FE2149296; **Location:** countryCode: TR; stateProvince: Hatay; locality: Belen Pass; verbatimElevation: 274 m; verbatimLatitude: 36.46; verbatimLongitude: 36.279; **Identification:** identifiedBy: Thomas J. Wood; **Event:** eventDate: 21-03-2009; **Record Level:** collectionCode: ZMUI
- **Type status:** Other material. **Occurrence:** recordedBy: Fatih Dikmen; individualCount: 5; sex: male; occurrenceID: 5AA93C3E-36EC-5E69-A6D5-CD71158A2270; **Location:** countryCode: TR; stateProvince: Hatay; locality: Belen Pass; verbatimElevation: 274 m; verbatimLatitude: 36.46; verbatimLongitude: 36.279; **Identification:** identifiedBy: Thomas J. Wood; **Event:** eventDate: 21-03-2009; **Record Level:** collectionCode: ZMUI
- **Type status:** Other material. **Occurrence:** recordedBy: Fatih Dikmen; individualCount: 1; sex: male; occurrenceID: AAD51886-409E-5706-AEF4-39F512BBAF59; **Location:** countryCode: TR; stateProvince: Mersin; locality: Mut-Silifke road; verbatimElevation: 124 m; verbatimLatitude: 36.569; verbatimLongitude: 33.449; **Identification:** identifiedBy: Thomas J. Wood; **Event:** eventDate: 22-03-2009; **Record Level:** collectionCode: ZMUI
- **Type status:** Other material. **Occurrence:** recordedBy: Fatih Dikmen; individualCount: 1; sex: male; occurrenceID: F0C16409-0124-5201-B8B3-3AB78A5C7DF4; **Location:** countryCode: TR; stateProvince: Muğla; locality: Marmaris; verbatimElevation: 9 m; verbatimLatitude: 36.985; verbatimLongitude: 28.216; **Identification:** identifiedBy: Thomas J. Wood; **Event:** eventDate: 02-04-2009; **Record Level:** collectionCode: ZMUI

#### Distribution

Afghanistan, Bulgaria, Cyprus, Greece, Iran, Iraq, Israel and the West Bank, Jordan, Lebanon, North Macedonia, Romania, Russia and Türkiye (Gusenleitner and Schwarz 2002, Osytshnjuk et al. 2005, Wood and Monfared 2022, Wood et al. 2024).

##### Distribution in Türkiye

**Figure 15.**
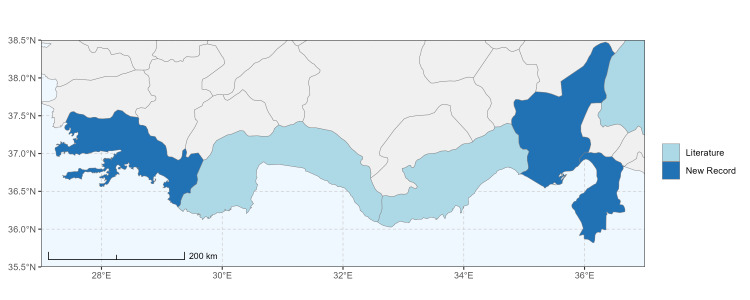
Distribution of *A. cinereophila* in the Mediterranean Region of Türkiye.

Balıkesir (Ayvalık), Mardin, Sakarya (Adapazarı) (Warncke 1966); Antalya (Paulus et al. 2001); Antep, Aydın, Diyarbakır, Kırklareli, Maraş, Mersin, Urfa (Hazır 2010, Hazır et al. 2014); Adana, Hatay, Mersin, Muğla (this study) (Fig. 15).

#### Flower records

**Asteraceae**: *Anthemis chia* L., *Crepis commutata* (Spreng.) Greuter, *Helichrysum stoechas* (L.) Moench (Lanuza et al. 2025); **Asteraceae**: *Anthemis chia* L., *Calendula arvensis* L., *Crepis sancta* (L.) Bornm. *Crepis foetida* L., **Brassicaceae**: *Sisymbrium* L.

### Andrena (Simandrena) cinnamonea

Warncke, 1975

69F24253-9E38-50CA-B86A-4B01E42D4DD5

#### Materials

- **Type status:** Other material. **Occurrence:** recordedBy: Fatih Dikmen; individualCount: 1; sex: female; occurrenceID: CD6D518F-F204-59FC-A1F5-47297AD6AB7E; **Location:** countryCode: TR; stateProvince: Hatay; locality: between Yayladağ and Samandağ; verbatimElevation: 520 m; verbatimLatitude: 35.949; verbatimLongitude: 36.052; **Identification:** identifiedBy: Thomas J. Wood; **Event:** eventDate: 22-03-2009; **Record Level:** collectionCode: ZMUI

#### Distribution

Türkiye (Gusenleitner and Schwarz 2002).

##### Distribution in Türkiye

**Figure 16.**
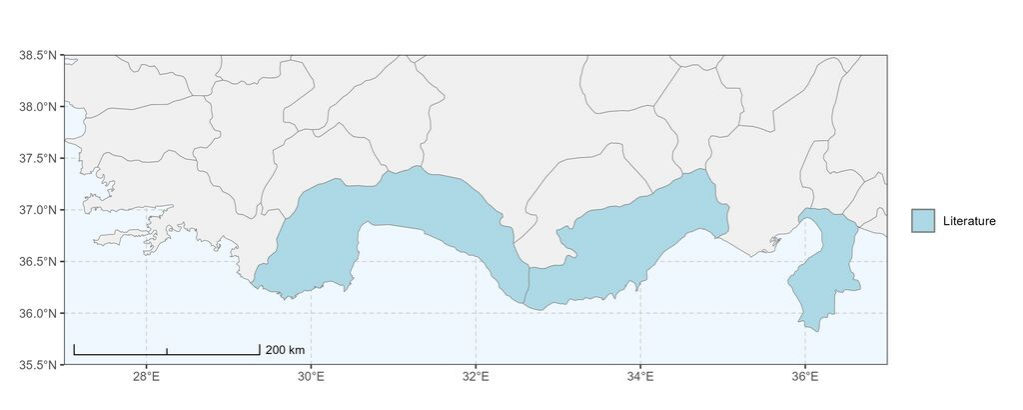
Distribution of *A. cinnamonea* in the Mediterranean Region of Türkiye.

Antalya (Akseki), Hatay (Antakya, Mersin (Mut) (Warncke 1974); Hatay (this study) (Fig. 16).

#### Flower records

**Asteraceae**: *Crepis sancta* (L.) Bornm.

### Andrena (Brachyandrena) colletiformis

Morawitz, 1873

8399AFB1-F000-5008-9CB0-733443C5F1C6

#### Materials

- **Type status:** Other material. **Occurrence:** recordedBy: Fatih Dikmen; individualCount: 1; sex: female; occurrenceID: FCF57C78-D4DD-522D-930F-419919B54530; **Location:** countryCode: TR; stateProvince: Mersin; county: Mut; locality: Güzlek Plateau; verbatimElevation: 1414 m; verbatimLatitude: 36.654; verbatimLongitude: 33.107; **Identification:** identifiedBy: Thomas J. Wood; **Event:** eventDate: 04-06-2009; **Record Level:** collectionCode: ZMUI
- **Type status:** Other material. **Occurrence:** recordedBy: Fatih Dikmen; individualCount: 1; sex: female; occurrenceID: DD6C5F87-0C82-5548-B9CE-E179C5A5B4CA; **Location:** countryCode: TR; stateProvince: Afyon; county: Dazkırı; municipality: Örtülü; verbatimElevation: 1192 m; verbatimLatitude: 37.899; verbatimLongitude: 29.753; **Identification:** identifiedBy: Thomas J. Wood; **Event:** eventDate: 12-06-2009; **Record Level:** collectionCode: ZMUI

#### Distribution

Albania, Bosnia and Herzegovina, Bulgaria, Corsica, Croatia, Cyprus, France, Greece, Iran, Israel and the West Bank, Italy, Kazakhstan, Kafkaslar, Morocco, North Macedonia, Portugal, Romania, Russia, Slovenia, Spain, Tunisia, Turkmenistan, Türkiye and Ukraine (Gusenleitner and Schwarz 2002, Osytshnjuk et al. 2005, Wood and Monfared 2022, Ascher and Pickering 2025).

##### Distribution in Türkiye

**Figure 17.**
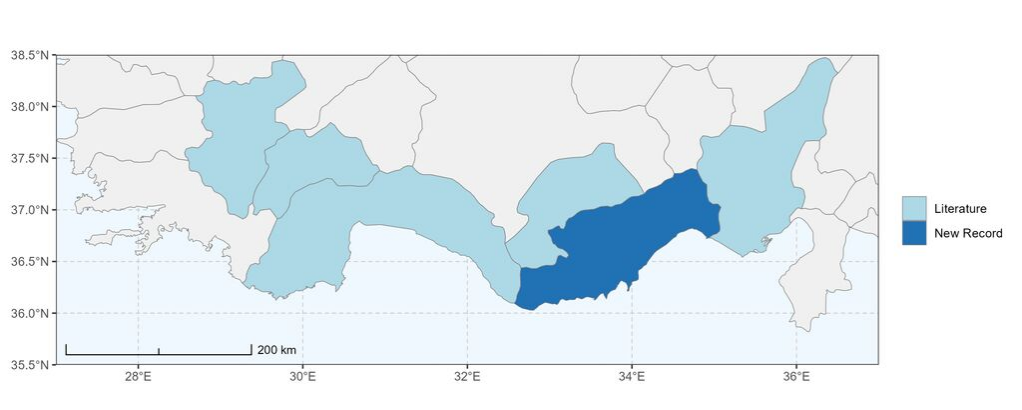
Distribution of *A. colletiformis* in the Mediterranean Region of Türkiye.

Adana, Amanos Mountains, Ankara, Toroslar (Warncke 1966); Adapazarı, Bilecik (Osmaneli), Bursa (Gemlik, Kurlu), Çanakkale, Denizli (Pamukkale), Edirne (İpsala), İstanbul (Selimpaşa), İzmir (Torbalı), Tekirdağ (Warncke 1969); Erzurum (Oltu, Tortum, Horasan, İspir) (Özbek 1976); Aydın (Kuşadası) (Ariana et al. 2009); Antalya, Burdur, Karaman, Manisa (Hazır 2010, Hazır et al. 2014); Afyon, Mersin (this study) (Fig. 17).

#### Flower records

**Apiaceae**: *Daucus carota* L. **Asteraceae**: *Pimpinella cretica* Poir. **Asteraceae**: *Helichrysum stoechas* (L.) Moench, *Sonchus tenerrimus* L., **Rosaceae**: *Spiraea japonica* L.f. (Lanuza et al. 2025); *Prangos uechtritzii* Boiss. & Hausskn., Cota coelopoda var. coelopoda (Boiss.) Boiss.

### Andrena (Truncandrena) combusta

Morawitz, 1876

94E52D26-F517-5C23-B552-5B2D741B56EC

#### Materials

- **Type status:** Other material. **Occurrence:** recordedBy: Fatih Dikmen; individualCount: 2; sex: female; occurrenceID: ADB344B9-1F2A-5596-BE1F-0A7BCB2EA22E; **Location:** countryCode: TR; stateProvince: Mersin; county: Mut; locality: Sarıkavak road; verbatimElevation: 665 m; verbatimLatitude: 36.565; verbatimLongitude: 33.63; **Identification:** identifiedBy: Thomas J. Wood; **Event:** eventDate: 25-04-2008; **Record Level:** collectionCode: ZMUI

#### Distribution

Afghanistan, Armenia, Azerbaijan, Iran, Kazakhstan, Pakistan, Syria, Tajikistan, Turkmenistan, Türkiye and Uzbekistan (Schuberth et al. 2001, Wood 2021b, Astafurova et al. 2022, Wood and Monfared 2022, Wood 2025).

##### Distribution in Türkiye

**Figure 18.**
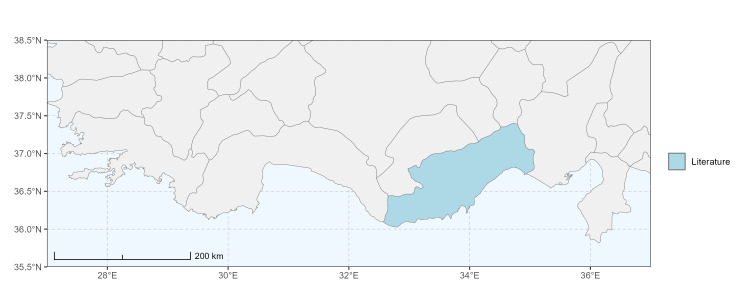
Distribution of *A. combusta* in the Mediterranean Region of Türkiye.

Ağrı (Ararat), Ankara (Gölbaşı), Nevşehir (Warncke 1974), Erzurum (Horasan), Iğdır (Özbek 1976); Mersin (Hazır 2010); Mersin (this study) (Fig. 18).

#### Flower records

**Brassicaceae**: *Diplotaxis tenuifolia* (L.) DC.

### Andrena (Cordandrena) cordialis

(Morawitz, 1877)

BBCA786D-4011-5E7B-B6BC-6110A7A862CF

#### Materials

- **Type status:** Other material. **Occurrence:** recordedBy: Fatih Dikmen; individualCount: 2; sex: female; occurrenceID: A231E502-8914-5B1F-8964-00E36677D3E3; **Location:** countryCode: TR; stateProvince: Burdur; county: Ağlasun; locality: 2-3 km after Köroğlu Pass; verbatimElevation: 1016 m; verbatimLatitude: 37,563 m; verbatimLongitude: 30,707 m; **Identification:** identifiedBy: Thomas J. Wood; **Event:** eventDate: 29-05-2009; **Record Level:** collectionCode: ZMUI

#### Distribution

Armenia, Azerbaijan, Bulgaria, Georgia, Greece, Hungary, Iran, North Macedonia, Romania, Russia, Serbia, Turkmenistan, Türkiye and Ukraine (Gusenleitner and Schwarz 2002, Osytshnjuk et al. 2005, Khodarahmi Ghahnavieh and Monfared 2019, Wood and Monfared 2022, Wood et al. 2024).

##### Distribution in Türkiye

**Figure 19.**
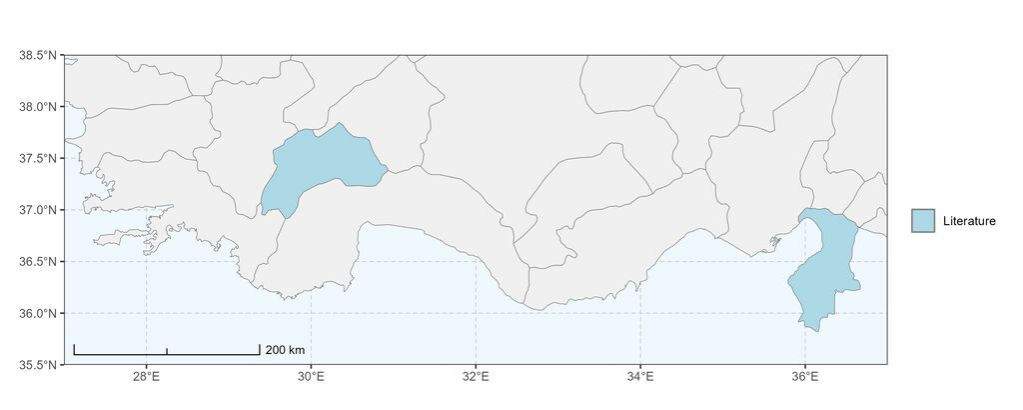
Distribution of *A. cordialis* in the Mediterranean Region of Türkiye.

Ankara (Şereflikoçhisar), Bursa (Mustafakemalpaşa), Erzurum, Iğdır, Kayseri (Yeşilhisar), Kırıkkale, Konya (Kulu, Sarayönü), Kütahya, Muş, Samsun, Sakarya (Adapazarı), Tokat (Niksar) (Warncke 1974); Aksaray, Ankara, Aydın, Burdur, Çorum, Hatay, Kastamonu, Konya, Kütahya, Manisa, Sivas (Hazır 2010, Hazır et al. 2014); Burdur (this study) (Fig. 19).

#### Flower records

**Brassicaceae**: *Brassica napus* L., *Lepidium draba* L. (Khodarahmi Ghahnavieh and Monfared 2019); **Asteraceae**: *Crepis foetida* L.

### Andrena (Poecilandrena) crassana

Warncke, 1965

7BDE05F1-0A87-56F2-B2EE-543280A1F769

#### Materials

- **Type status:** Other material. **Occurrence:** recordedBy: Fatih Dikmen; individualCount: 1; sex: female; occurrenceID: 93616C76-075B-5C9F-9407-ACE18EF50C5E; **Location:** countryCode: TR; stateProvince: Burdur; county: Ağlasun; locality: <10 km after Köroğlu Pass; verbatimElevation: 948 m; verbatimLatitude: 37.636; verbatimLongitude: 30.684; **Identification:** identifiedBy: Thomas J. Wood; **Event:** eventDate: 29-05-2009; **Record Level:** collectionCode: ZMUI

#### Distribution

Cyprus, Greece, Iran, Iraq, Israel and West Bank, Jordan, Lebanon, North Macedonia, Syria and Türkiye (Gusenleitner and Schwarz 2002, Pisanty et al. 2018, Wood and Monfared 2022, Wood et al. 2024).

##### Distribution in Türkiye

**Figure 20.**
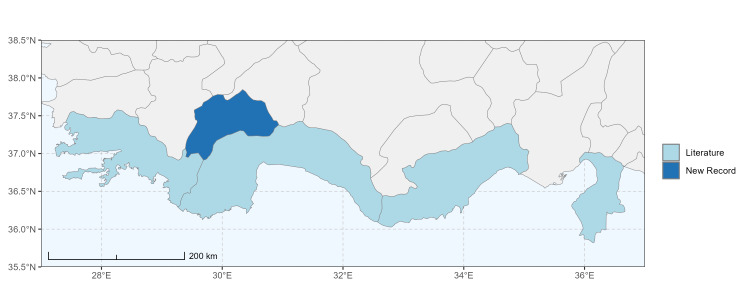
Distribution of *A. crassana* in the Mediterranean Region of Türkiye.

Antalya (Finike), Bursa (Karacabey), Mersin (Anamur), Muğla (Marmaris) (Warncke 1974); Antalya, Hatay, Kütahya, Muğla (Hazır 2010, Hazır et al. 2014); Burdur (this study) (Fig. 20).

#### Flower records

**Asteraceae**: *Crepis foetida* L.

### Andrena (Cordandrena) cypria

Pittioni, 1950

AA125979-BBA4-50C8-BBA3-87942D30A8BC

#### Materials

- **Type status:** Other material. **Occurrence:** recordedBy: Fatih Dikmen; individualCount: 1; sex: female; occurrenceID: 994D2A27-ED8F-5EB3-8740-DBC5F9E511D5; **Location:** countryCode: TR; stateProvince: Muğla; county: Marmaris; municipality: Turunç; locality: Datça road; verbatimElevation: 347 m; verbatimLatitude: 36.775; verbatimLongitude: 28.221; **Identification:** identifiedBy: Thomas J. Wood; **Event:** eventDate: 03-04-2009; **Record Level:** collectionCode: ZMUI

#### Distribution

Cyprus, Iran, Israel and the West Bank, Jordan, Syria and Türkiye (Gusenleitner and Schwarz 2002, Wood and Monfared 2022).

##### Distribution in Türkiye

**Figure 21.**
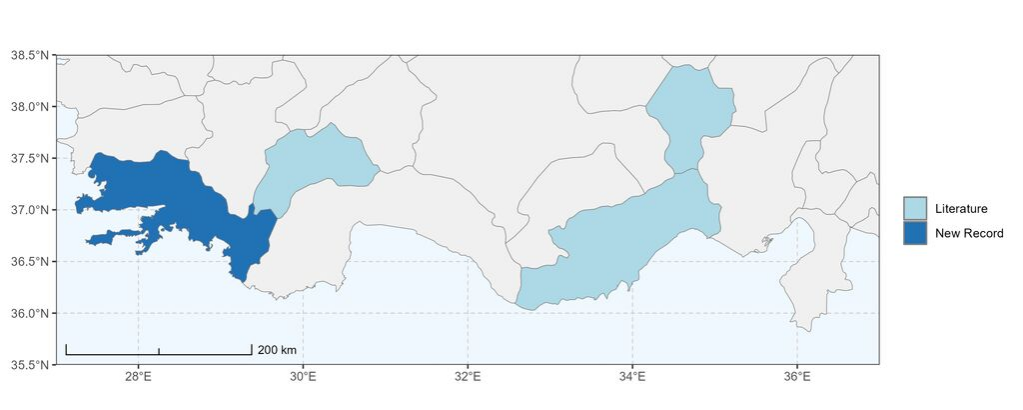
Distribution of *A. cypria* in the Mediterranean Region of Türkiye.

Konya (Sarayönü) (Warncke 1966); Ankara (Gölbaşı, Şereflikoçhisar), Erzurum, Kayseri (Yeşilhisar), Kırşehir (Kaman), Mersin (Mut), Muş, Nevşehir (Ürgüp), Niğde (Ulukışla), Sivas (Gürün) (Warncke 1974, Özbek 1976); Aksaray, Ankara, Aydın, Burdur, Kırşehir, Konya, Manisa, Sivas, Yozgat (Hazır 2010, Hazır et al. 2014); Muğla (this study) (Fig. 21).

#### Flower records

**Brassicaceae**: *Alyssum* L., *Lepidium draba* L. **Berberidaceae**: *Bongardia chrysogonum* (L.) Spach **Papaveraceae**: *Papaver* L. (Khodarahmi Ghahnavieh and Monfared 2019); **Asteraceae**: *Anthemis chia* L.

### Andrena discordia

Wood, 2023

75979BC5-DDB1-5103-A878-9EDAD79CD768

#### Materials

- **Type status:** Other material. **Occurrence:** recordedBy: Fatih Dikmen; individualCount: 1; sex: female; occurrenceID: 6AC52A2D-2D56-5C79-821B-32ECD984DACD; **Location:** countryCode: TR; stateProvince: Mersin; county: Mut; locality: Güzlek Plateau; verbatimElevation: 1414 m; verbatimLatitude: 36.654; verbatimLongitude: 33.107; **Identification:** identifiedBy: Thomas J. Wood; **Event:** eventDate: 04-06-2009; **Record Level:** collectionCode: ZMUI

#### Distribution

Iran, Iraq and Türkiye (Wood 2023a, Wood et al. 2024).

##### Distribution in Türkiye

**Figure 22.**
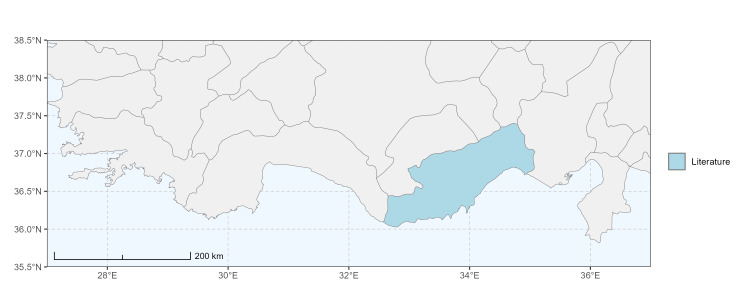
Distribution of *A. discordia* in the Mediterranean Region of Türkiye.

Hakkari, Mersin (Wood 2023a); Mersin (this study) (Fig. 22).

#### Flower records

**Apiaceae**: *Prangos uechtritzii* Boiss. & Hausskn.

### Andrena (Simandrena) dorsata

(Kirby, 1802)

78575449-B704-5325-B8E6-6E5EA229A558

#### Materials

- **Type status:** Other material. **Occurrence:** recordedBy: Fatih Dikmen; individualCount: 6; sex: female; occurrenceID: CA5182FB-17BA-51AB-AECE-C6123241F290; **Location:** countryCode: TR; stateProvince: Niğde; county: Çamardı; verbatimElevation: 1570 m; verbatimLatitude: 37.887; verbatimLongitude: 35.112; **Identification:** identifiedBy: Thomas J. Wood; **Event:** eventDate: 23-05-2008; **Record Level:** collectionCode: ZMUI

#### Distribution

Albania, Austria, Belgium, Bulgaria, Corsica, Croatia, Estonia, France, Germany, Great Britain, Greece, Hungary, Iran, Italy, Kazakhstan, Latvia, Lithuania, Luxembourg, Morocco, Netherlands, Poland, Portugal, Romania, Russia, Slovakia, Slovenia, Spain, Sweden, Switzerland, Türkiye and Ukraine (Wood and Monfared 2022, Ascher and Pickering 2025).

##### Distribution in Türkiye

**Figure 23.**
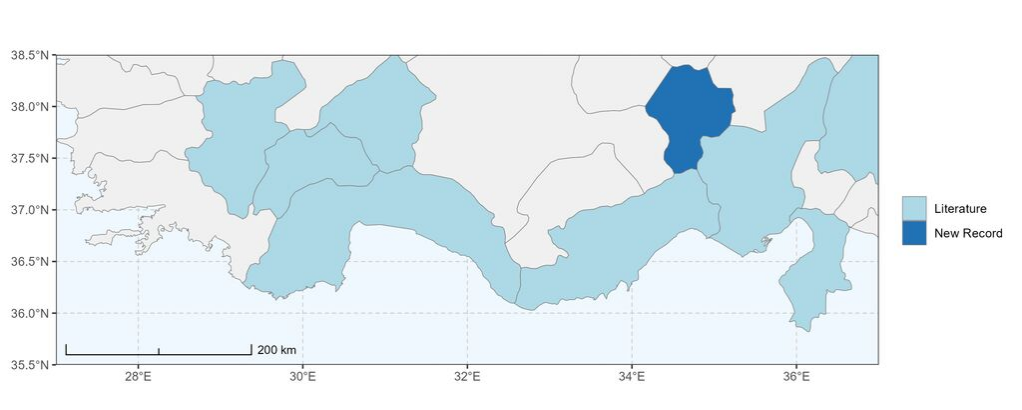
Distribution of *A. dorsata* in the Mediterranean Region of Türkiye.

Adana, Adapazarı, Eskişehir (Sivrihisar), İstanbul, Konya, Tekirdağ, Toroslar (Gülek) (Warncke 1966); Afyon (Çay), Ankara (Delice), Hatay (Samandağ) (Warncke 1969); Bingöl, Erzincan, Erzurum (Oltu), Kars (Kağızman), Muş (Özbek 1976); Adana, Afyon, Amasya, Ankara, Antalya, Aydın, Burdur, Çorum, Denizli, Hatay, Isparta, Maraş, Karabük, Kastamonu, Kırıkkale, Kırşehir, Konya, Mersin, Samsun (Hazır 2010); Niğde (this study) (Fig. 23).

#### Flower records

**Convolvulaceae**: *Convolvulus* L. **Fabaceae**: *Glycyrrhiza glabra* L., *Medicago sativa* L. (Khodarahmi Ghahnavieh and Monfared 2019); **Alismataceae**: *Alisma plantago-aquatica* L. **Apiaceae**: *Anthriscus sylvestris* (L.) Hoffm., *Chaerophyllum temulum* L., *Conopodium majus* (Gouan) Loret, *Daucus carota* L., *Daucus* L., *Heracleum sphondylium* L., *Oenanthe crocata* L., *Torilis japonica* (Houtt.) DC. **Asteraceae**: *Bellis perennis* L., *Centaurea nigra* L., *Cirsium arvense* (L.) Scop., *Cirsium palustre* (L.) Scop., *Cirsium* Mill., *Crepis capillaris* (L.) Wallr., *Crepis* L., *Hypochaeris radicata* L., *Leontodon hispidus* L., *Leucanthemum vulgare* Lam., *Picris echioides* L., *Picris hieracioides* L., *Pulicaria dysenterica* (L.) Bernh., *Senecio jacobaea* L., *Taraxacum* agg. F.H.Wigg., *Taraxacum officinale* agg. F.H.Wigg., *Tripleurospermum inodorum* (L.) Sch.Bip. **Brassicaceae**: *Alliaria petiolata* (M.Bieb.) Cavara & Grande, *Barbarea vulgaris* W.T.Aiton, *Brassica napus* L., *Brassica* L., *Pimpinella saxifraga* L., *Raphanus raphanistrum* L., *Sinapis alba* L., *Sinapis arvensis* L., *Sisymbrium officinale* (L.) Scop. **Convolvulaceae**: *Convolvulus arvensis* L. **Cornaceae**: *Cornus alba* L. **Cucurbitaceae**: *Bryonia dioica* Bojer **Ericaceae**: *Calluna vulgaris* (L.) Hull, *Erica cinerea* L., *Erica* Tourn. ex L., *Erica tetralix* L. **Fabaceae**: *Medicago lupulina* L., *Trifolium repens* L., *Ulex europaeus* L., *Ulex minor* Roth **Fagaceae**: *Castanea sativa* Mill. **Geraniaceae**: *Geranium* Tourn. ex L. **Hypericaceae**: *Hypericum perforatum* L. **Lamiaceae**: *Clinopodium vulgare* L., *Mentha aquatica* L. **Orobanchaceae**: *Odontites vernus* (Bellardi) Dumort., *Vicia sativa* L. **Papaveraceae**: *Papaver rhoeas* L. **Plantaginaceae**: *Veronica chamaedrys* L. **Ranunculaceae**: *Ranunculus repens* L. **Rosaceae**: *Crataegus monogyna* Jacq., *Filipendula ulmaria* (L.) Maxim., *Fragaria* L., *Potentilla erecta* (L.) Raeusch., *Potentilla* L., *Prunus* L., *Prunus spinosa* L., *Rosa* L., *Rubus fruticosus* agg. L., *Rubus idaeus* L., *Rubus* L. **Salicaceae**: *Salix* L. **Sapindaceae**: *Acer campestre* L., *Acer* L. **Viburnaceae**: *Viburnum opulus* L., *Viburnum* L. (Balfour et al. 2022); **Apiaceae**: *Anthriscus sylvestris* (L.) Hoffm., *Daucus carota* L., *Heracleum sphondylium* L., *Torilis japonica* (Houtt.) DC. **Asteraceae**: *Bellis perennis* L., *Centaurea nigra* L., *Cirsium arvense* (L.) Scop., *Cirsium vulgare* (Savi) Ten., *Crepis capillaris* (L.) Wallr., *Matricaria chamomilla* L., *Taraxacum* F.H.Wigg., *Tanacetum vulgare* L. **Brassicaceae**: *Brassica juncea* (L.) Czern., *Brassica napus* L., *Lepidium draba* L., *Rorippa sylvestris* (L.) Besser, *Sisymbrium loeselii* L. **Caprifoliaceae**: *Valeriana officinalis* L. **Caryophyllaceae**: *Arenaria serpyllifolia* L. **Cornaceae**: *Cornus alba* L. **Euphorbiaceae**: *Euphorbia esula* L. **Fabaceae**: *Anthyllis montana* L., *Lotus corniculatus* L., *Melilotus albus* Medik., *Melilotus officinalis* (L.) Lam., *Trifolium repens* L. **Hypericaceae**: *Hypericum perforatum* L. **Lamiaceae**: *Lamium amplexicaule* L. **Onagraceae**: *Oenothera biennis* L. **Orchidaceae**: *Orchis mascula* (L.) L. **Ranunculaceae**: *Ranunculus acris* L. **Rosaceae**: *Filipendula ulmaria* (L.) Maxim., *Potentilla erecta* (L.) Raeusch., *Potentilla reptans* L., *Pyrus communis* L., *Rubus* L., *Rubus fruticosus* L., *Sanguisorba minor* Scop. **Scrophulariaceae**: *Verbascum densiflorum* Bertol. **Solanaceae**: *Solanum tuberosum* L. (Lanuza et al. 2025); **Brassicaceae**: *Brassica* L.

### Andrena (Micrandrena) enslinella

Stöckhert, 1924

486B74DB-D966-5BF3-AC0D-7B4061E15D13

#### Materials

- **Type status:** Other material. **Occurrence:** recordedBy: Fatih Dikmen; individualCount: 2; sex: female; occurrenceID: 6FDC8C99-7382-573D-9939-32B3B0BA6919; **Location:** countryCode: TR; stateProvince: Niğde; county: Çamardı; verbatimElevation: 1570 m; verbatimLatitude: 37.886; verbatimLongitude: 35.111; **Identification:** identifiedBy: Thomas J. Wood; **Event:** eventDate: 23-05-2008; **Record Level:** collectionCode: ZMUI

#### Distribution

Austria, Bulgaria, Croatia, Czechia, Greece, Hungary, Iran, Italy, North Macedonia, Poland, Russia, Serbia, Slovakia, Slovenia, Türkiye and Ukraine (Gusenleitner and Schwarz 2002, Wood and Monfared 2022).

##### Distribution in Türkiye

**Figure 24.**
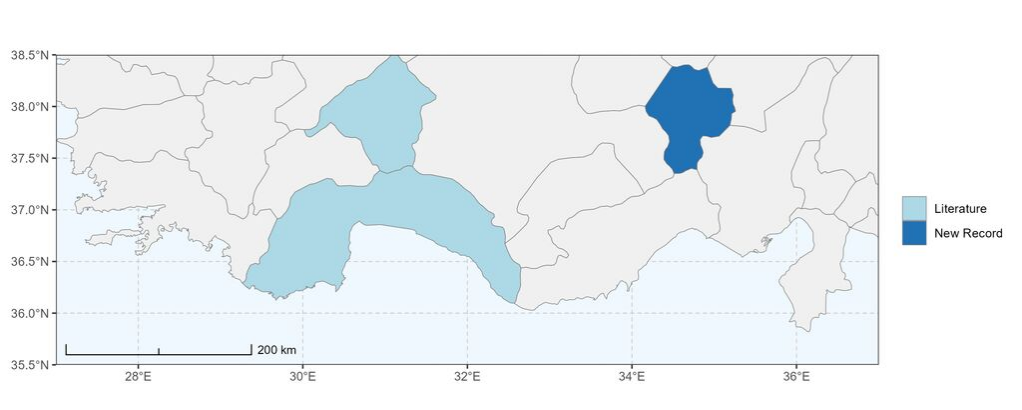
Distribution of *A. enslinella* in the Mediterranean Region of Türkiye.

Ankara, Çorum, İstanbul, Konya (Akşehir), Sivas (Gürün) (Warncke 1974); Antalya (Güzelsu) (Ebmer 2005); Ankara, Isparta (Gelendost) (Hazır et al. 2014); Niğde (this study) (Fig. 24 and Fig. 14D-F).

#### Flower records

**Rosaceae**: *Potentilla reptans* L. (Lanuza et al. 2025); **Brassicaceae**: *Brassica* L.

### Andrena (Campylogaster) erberi

Morawitz, 1871

1B74023D-D78A-5E2C-8B3C-23775AFCBC9A

#### Materials

- **Type status:** Other material. **Occurrence:** recordedBy: Fatih Dikmen; individualCount: 1; sex: female; occurrenceID: C6132760-7237-5316-8DEC-6403731EAE9B; **Location:** countryCode: TR; stateProvince: Mersin; county: Mut; locality: Sarıkavak road; verbatimElevation: 665 m; verbatimLatitude: 36.565; verbatimLongitude: 33.63; **Identification:** identifiedBy: Thomas J. Wood; **Event:** eventDate: 25-04-2008; **Record Level:** collectionCode: ZMUI

#### Distribution

Bulgaria, Egypt, Greece, Iran, North Macedonia, Romania, Türkiye and the Caucasus (Gusenleitner and Schwarz 2002, Wood and Monfared 2022).

##### Distribution in Türkiye

**Figure 25.**
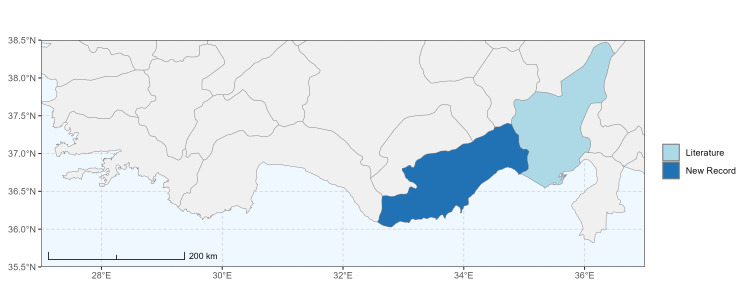
Distribution of *A. erberi* in the Mediterranean Region of Türkiye.

Adana (Ceyhan), Ankara, Nevşehir (Ürgüp) (Warncke 1966); Konya (Hazır 2010); Mersin (this study) (Fig. 25).

#### Flower records

**Resedaceae**: Reseda lutea var. lutea L.

### Andrena (Chlorandrena) exquisita

Warncke, 1975

B2ECB191-77EB-514D-9C6A-34313722A3AA

#### Materials

- **Type status:** Other material. **Occurrence:** recordedBy: Fatih Dikmen; individualCount: 1; sex: female; occurrenceID: 853BF550-A6AF-5545-BF70-3ACE55B4C38A; **Location:** countryCode: TR; stateProvince: Hatay; locality: between Yayladağ and Samandağ; verbatimElevation: 440 m; verbatimLatitude: 35.923; verbatimLongitude: 36.054; **Identification:** identifiedBy: Thomas J. Wood; **Event:** eventDate: 22-03-2009; **Record Level:** collectionCode: ZMUI
- **Type status:** Other material. **Occurrence:** recordedBy: Fatih Dikmen; individualCount: 6; sex: male; occurrenceID: 77F462A5-E1DD-5634-A5C1-CB80E16BC759; **Location:** countryCode: TR; stateProvince: Hatay; locality: between Yayladağ and Samandağ; verbatimElevation: 440 m; verbatimLatitude: 35.923; verbatimLongitude: 36.054; **Identification:** identifiedBy: Thomas J. Wood; **Event:** eventDate: 22-03-2009; **Record Level:** collectionCode: ZMUI

#### Distribution

Bulgaria, Cyprus, Georgia, Iran, Israel and the West Bank, Jordan, Lebanon, Russia, Syria and Türkiye (Gusenleitner and Schwarz 2002, Pisanty et al. 2018, Wood et al. 2020).

##### Distribution in Türkiye

**Figure 26.**
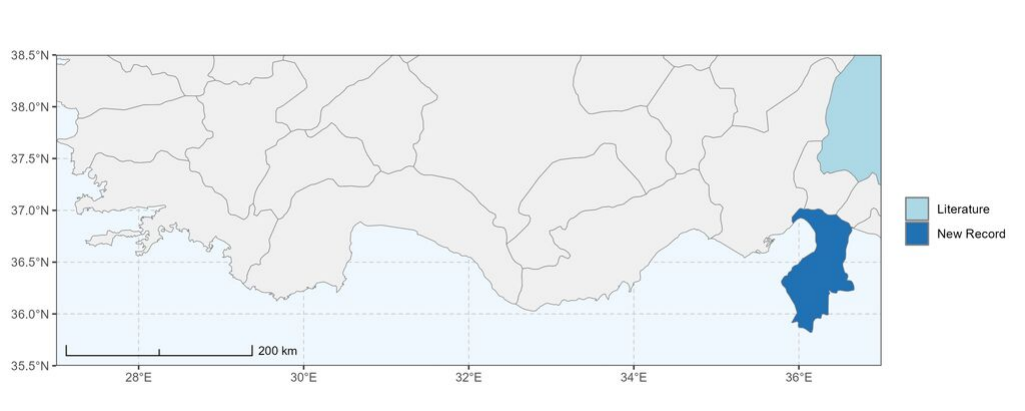
Distribution of *A. exquisita* in the Mediterranean Region of Türkiye.

Çanakkale (Bigadiç), İstanbul (Büyükdere) (Warncke 1974, Blank and Kraus 1994); Aydın, Bursa, Çanakkale, Maraş, Kırklareli, Manisa (Hazır 2010, Hazır et al. 2014); Hatay (this study) (Fig. 26).

#### Flower records

**Asteraceae**: *Crepis sancta* (L.) Bornm.

### Andrena (Leimelissa) fallax

Eversmann, 1852

7F6701A8-863B-548F-9FAD-B9278EE9697D

#### Materials

- **Type status:** Other material. **Occurrence:** recordedBy: Fatih Dikmen; individualCount: 1; sex: female; occurrenceID: E4730366-05C0-5172-BD7A-E645E106A41E; **Location:** countryCode: TR; stateProvince: Antalya; locality: Saklıkent road; verbatimElevation: 1759 m; verbatimLatitude: 36.87; verbatimLongitude: 30.343; **Identification:** identifiedBy: Thomas J. Wood; **Event:** eventDate: 11-06-2009; **Record Level:** collectionCode: ZMUI
- **Type status:** Other material. **Occurrence:** recordedBy: Fatih Dikmen; individualCount: 1; sex: male; occurrenceID: E7C0FA88-55C9-51B1-90DB-A2DBCC36BA0A; **Location:** countryCode: TR; stateProvince: Antalya; locality: Saklıkent road; verbatimElevation: 1759 m; verbatimLatitude: 36.87; verbatimLongitude: 30.343; **Identification:** identifiedBy: Thomas J. Wood; **Event:** eventDate: 11-06-2009; **Record Level:** collectionCode: ZMUI
- **Type status:** Other material. **Occurrence:** recordedBy: Fatih Dikmen; individualCount: 1; sex: female; occurrenceID: 2C455CF4-A8A2-57F1-A320-4BD1F56044DC; **Location:** countryCode: TR; stateProvince: Mersin; county: Mut; locality: Güzlek Plateau; verbatimElevation: 1414 m; verbatimLatitude: 36.654; verbatimLongitude: 33.108; **Identification:** identifiedBy: Thomas J. Wood; **Event:** eventDate: 04-06-2009; **Record Level:** collectionCode: ZMUI

#### Distribution

Albania, Armenia, Azerbaijan, Bulgaria, Georgia, Greece, Hungary, Iran, Romania, Syria and Türkiye (Gusenleitner and Schwarz 2002, Osytshnjuk et al. 2008, Wood and Monfared 2022, Wood 2023a).

##### Distribution in Türkiye

**Figure 27.**
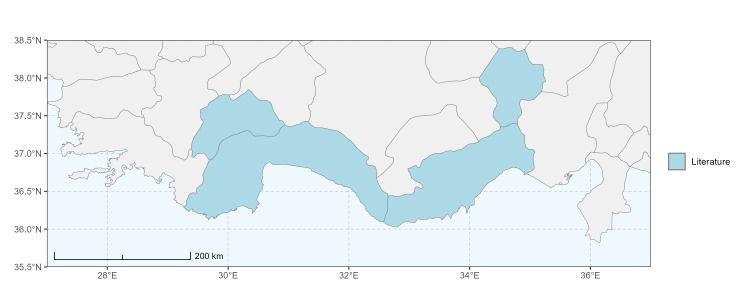
Distribution of *A. fallax* in the Mediterranean Region of Türkiye.

Konya (Beyşehir) (Warncke 1966); Çanakkale (Eceabat), Kırşehir, Nevşehir (Ürgüp), Niğde (Çiftehan), Mersin (Mut), Samsun (Bafra), Muş (Warncke 1974); Erzurum (Oltu, İspir, Tortum) (Özbek 1976); Antalya, Burdur (Hazır 2010); Antalya, Mersin (this study) (Fig. 27).

#### Flower records

**Brassicaceae**: *Lepidium chalepense* L.

### Andrena (Holandrena) flavilabris

Schenck, 1874

42C37259-1D96-58A1-BA47-033FB78AB37C

#### Materials

- **Type status:** Other material. **Occurrence:** recordedBy: Fatih Dikmen; individualCount: 1; sex: female; occurrenceID: 8E062BBF-0562-542C-B8EB-DDB86EB41DAE; **Location:** countryCode: TR; stateProvince: Burdur; locality: Antalya road, Korkuteli turn; verbatimElevation: 783 m; verbatimLatitude: 37.313; verbatimLongitude: 30.49; **Identification:** identifiedBy: Thomas J. Wood; **Event:** eventDate: 29-05-2009; **Record Level:** collectionCode: ZMUI

#### Distribution

Croatia, Cyprus, France, Germany, Greece, Italy, Spain, Switzerland, Türkiye and Ukraine (Ascher and Pickering 2025).

##### Distribution in Türkiye

**Figure 28.**
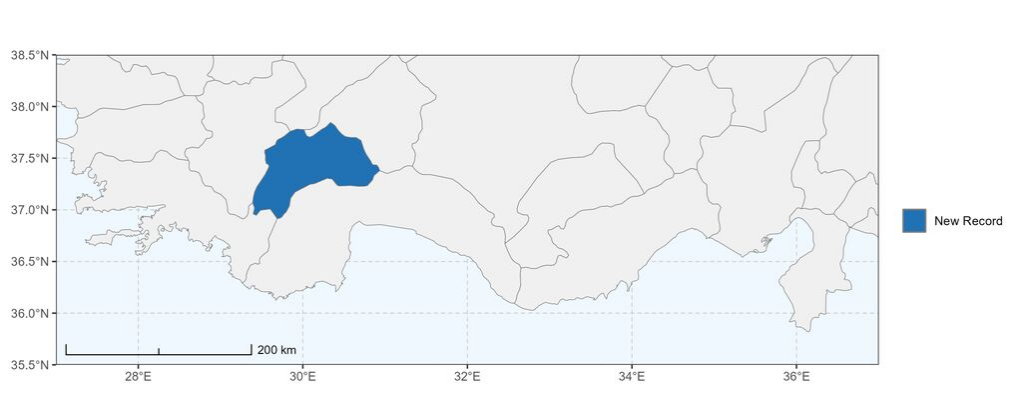
Distribution of *A. flavilabris* in the Mediterranean Region of Türkiye.

**Figure 29.**
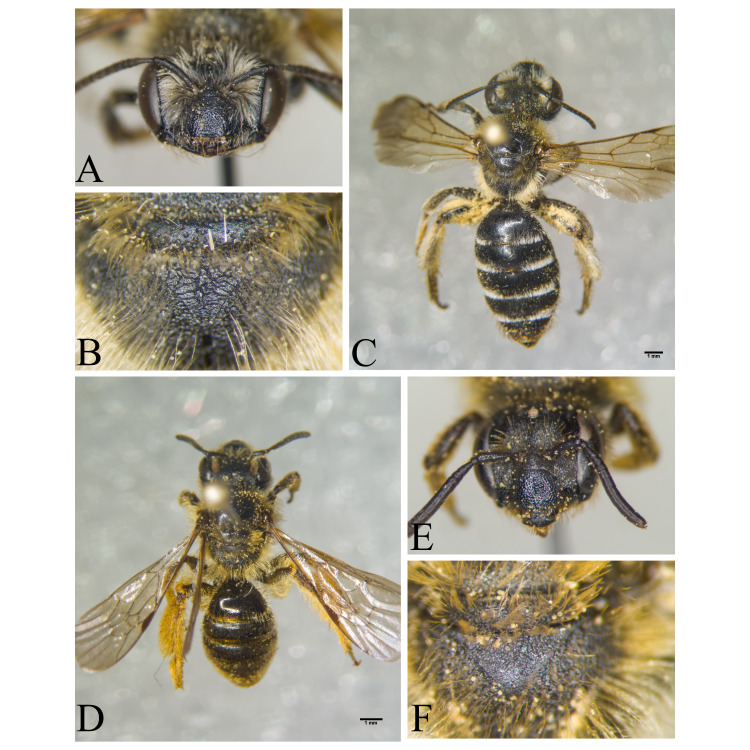
*Andrena flavilabris*, female. Head, frontal view (A); propodeum, dorsal view (B); habitus, dorsal view (C). *Andrena fulvago*, female. Habitus, dorsal view (D), head, frontal view (E), propodeum, dorsal view (F).

Ankara (Şereflikoçhisar) (Warncke 1966); Konya (Kulu), Samsun (Bafra) (Warncke 1974); Erzurum, Muş (Özbek 1976); Burdur (this study) (Fig. 28 and Fig. 29A-C).

#### Flower records

**Brassicaceae**: *Hirschfeldia incana* (L.) Lagr.-Foss.

### Andrena (Melandrena) flavipes

Panzer, 1799

91325AD7-B137-53BB-BE56-36344E67180E

#### Materials

- **Type status:** Other material. **Occurrence:** recordedBy: Fatih Dikmen; individualCount: 8; sex: female; occurrenceID: 7433D05D-0E9E-5ACF-B018-07C3C239A266; **Location:** countryCode: TR; stateProvince: Antalya; county: Side; locality: Side hotels; verbatimElevation: 13 m; verbatimLatitude: 36.79; verbatimLongitude: 31.399; **Identification:** identifiedBy: Thomas J. Wood; **Event:** eventDate: 13-04-2009; **Record Level:** collectionCode: ZMUI
- **Type status:** Other material. **Occurrence:** recordedBy: Fatih Dikmen; individualCount: 1; sex: female; occurrenceID: 85E3A9F3-B5C0-5CF2-AFDE-C3CB5B292355; **Location:** countryCode: TR; stateProvince: Antalya; locality: between Side-Belek; verbatimElevation: 16 m; verbatimLatitude: 36.843; verbatimLongitude: 31.292; **Identification:** identifiedBy: Thomas J. Wood; **Event:** eventDate: 13-04-2009; **Record Level:** collectionCode: ZMUI
- **Type status:** Other material. **Occurrence:** recordedBy: Fatih Dikmen; individualCount: 1; sex: female; occurrenceID: FC87C27B-517B-569C-B195-9CC31B0D0446; **Location:** countryCode: TR; stateProvince: Kahramanmaraş; locality: Andırlı-Kadirli road, Torun village; verbatimElevation: 659 m; verbatimLatitude: 37.503; verbatimLongitude: 36.345; **Identification:** identifiedBy: Thomas J. Wood; **Event:** eventDate: 19-07-2009; **Record Level:** collectionCode: ZMUI
- **Type status:** Other material. **Occurrence:** recordedBy: Fatih Dikmen; individualCount: 2; sex: female; occurrenceID: ADFEFDAC-819D-52DD-84C1-6CD48DD39519; **Location:** countryCode: TR; stateProvince: Mersin; locality: Gülnar-Ermenek road; verbatimElevation: 1241 m; verbatimLatitude: 36.366; verbatimLongitude: 33.234; **Identification:** identifiedBy: Thomas J. Wood; **Event:** eventDate: 07-06-2009; **Record Level:** collectionCode: ZMUI
- **Type status:** Other material. **Occurrence:** recordedBy: Fatih Dikmen; individualCount: 1; sex: female; occurrenceID: 58195365-8019-53EB-91A0-90A21215ACCD; **Location:** countryCode: TR; stateProvince: Antalya; county: Side; verbatimElevation: 1 m; verbatimLatitude: 36.776; verbatimLongitude: 31.414; **Identification:** identifiedBy: Thomas J. Wood; **Event:** eventDate: 13-04-2009; **Record Level:** collectionCode: ZMUI
- **Type status:** Other material. **Occurrence:** recordedBy: Fatih Dikmen; individualCount: 1; sex: female; occurrenceID: 3AE8B396-A39A-58B3-B1A6-769E6713F07C; **Location:** countryCode: TR; stateProvince: Afyonkarahisar; county: Dazkırı; locality: Örtülü village exit; verbatimElevation: 891 m; verbatimLatitude: 37.899; verbatimLongitude: 29.753; **Identification:** identifiedBy: Thomas J. Wood; **Event:** eventDate: 12-06-2009; **Record Level:** collectionCode: ZMUI
- **Type status:** Other material. **Occurrence:** recordedBy: Fatih Dikmen; individualCount: 1; sex: female; occurrenceID: 88083B6E-D494-52D3-A53D-0D715E21D6DF; **Location:** countryCode: TR; stateProvince: Antalya; locality: Korkuteli-Finike road, Yeşilbayır Plateau; verbatimElevation: 1438 m; verbatimLatitude: 36.956; verbatimLongitude: 30.058; **Identification:** identifiedBy: Thomas J. Wood; **Event:** eventDate: 10-06-2009; **Record Level:** collectionCode: ZMUI
- **Type status:** Other material. **Occurrence:** recordedBy: Fatih Dikmen; individualCount: 2; sex: female; occurrenceID: 54A5EBAF-582C-5D44-B00E-7154CBAB9504; **Location:** countryCode: TR; stateProvince: Hatay; locality: Belen-Radar road; verbatimElevation: 1124 m; verbatimLatitude: 36.518; verbatimLongitude: 36.234; **Identification:** identifiedBy: Thomas J. Wood; **Event:** eventDate: 30-06-2009; **Record Level:** collectionCode: ZMUI
- **Type status:** Other material. **Occurrence:** recordedBy: Fatih Dikmen; individualCount: 1; sex: female; occurrenceID: 9242A9FC-58A4-5BE1-A04A-84E7183C1186; **Location:** countryCode: TR; stateProvince: Antalya; locality: Akseki-Konya road, 2 km before Cevizli turn; verbatimElevation: 1262 m; verbatimLatitude: 37.117; verbatimLongitude: 31.777; **Identification:** identifiedBy: Thomas J. Wood; **Event:** eventDate: 09-06-2009; **Record Level:** collectionCode: ZMUI
- **Type status:** Other material. **Occurrence:** recordedBy: Fatih Dikmen; individualCount: 1; sex: male; occurrenceID: 03E8DFCF-4C66-5F93-900D-92029FC65105; **Location:** countryCode: TR; stateProvince: Kahramanmaraş; locality: Göksun road; verbatimElevation: 901 m; verbatimLatitude: 37.727; verbatimLongitude: 36.693; **Identification:** identifiedBy: Thomas J. Wood; **Event:** eventDate: 02-07-2009; **Record Level:** collectionCode: ZMUI
- **Type status:** Other material. **Occurrence:** recordedBy: Fatih Dikmen; individualCount: 6; sex: female; occurrenceID: D24761B2-1A84-5D40-8877-D7F3A37AE1AB; **Location:** countryCode: TR; stateProvince: Niğde; county: Çamardı; verbatimElevation: 1570 m; verbatimLatitude: 37.886; verbatimLongitude: 35.111; **Identification:** identifiedBy: Thomas J. Wood; **Event:** eventDate: 23-05-2008; **Record Level:** collectionCode: ZMUI
- **Type status:** Other material. **Occurrence:** recordedBy: Fatih Dikmen; individualCount: 3; sex: female; occurrenceID: 87F5FB3D-39FD-5472-A48B-D376D11BF9A5; **Location:** countryCode: TR; stateProvince: Niğde; locality: Karagöl road, Madenköy exit; verbatimElevation: 1756 m; verbatimLatitude: 37.455; verbatimLongitude: 34.623; **Identification:** identifiedBy: Thomas J. Wood; **Event:** eventDate: 22-05-2008; **Record Level:** collectionCode: ZMUI

#### Distribution

**Distribution.** Albania, Algeria, Armenia, Austria, Afghanistan, Belgium, Bosnia and Herzegovina, Bulgaria, China, Croatia, Cyprus, Denmark, Egypt, France, Georgia, Germany, Great Britain, Greece, Hungary, Iran, Israel and the West Bank, Italy, India, Kazakhstan, Latvia, Lebanon, Lithuania, Luxembourg, Malta, Moldova, Morocco, Nepal, Netherlands, North Macedonia, Pakistan, Poland, Portugal, Romania, Russia, Slovakia, Slovenia, Sweden, Syria, Tunisia, Turkmenistan, Türkiye, Ukraine and Uzbekistan (Gusenleitner and Schwarz 2002, Wood and Monfared 2022, Wood 2024, Wood et al. 2024, Ascher and Pickering 2025).

##### Distribution in Türkiye

**Figure 30.**
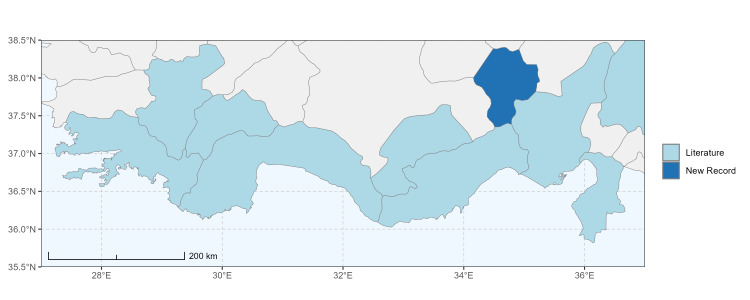
Distribution of *A. flavipes* in the Mediterranean Region of Türkiye.

Balıkesir (Edremit), Bilecik, Bolu (Abant), Burdur, Çanakkale (Truva, Gelibolu), Denizli (Pamukkale), İstanbul (Selimpaşa), İzmir (Ensetepe), Karaman, Samsun (Dikbıyık) (Warncke 1969); Erzurum (Oltu, Tortum, İspir, Horasan, Hınıs, Aşkale), Iğdır, Tunceli, Elazığ (Özbek 1976); Adana, Afyon, Aksaray, Amasya, Ankara, Antalya, Aydın, Bayburt, Bolu, Burdur, Çanakkale, Çankırı, Çorum, Denizli, Düzce, Hatay, Kahramanmaraş, Karabük, Kastamonu, Kırıkkale, Kırşehir, Konya, Kütahya, Manisa, Mersin, Muğla, Nevşehir, Samsun, Sivas, Yozgat (Hazır 2010, Güler 2011, Hazır et al. 2014); Afyon, Antalya, Hatay, Kahramanmaraş, Mersin, Niğde (this study) (Fig. 30).

#### Flower records

**Alliaceae**: *Allium cepa* L., *Tragopogon* L. **Asteraceae**: *Centaurea* L., *Cirsium* Mill. **Berberidaceae**: *Bongardia chrysogonum* (L.) Spach **Brassicaceae**: *Alyssum* L., *Brassica napus* L., *Lepidium draba* L., *Descurainia sophia* (L.) Webb ex Prantl, *Lepidium sativum* L. **Convolvulaceae**: *Convolvulus* L. **Fabaceae**: *Medicago sativa* L., *Melilotus officinalis* (L.) Lam. **Papaveraceae**: *Papaver* L. (Khodarahmi Ghahnavieh and Monfared 2019); **Amaryllidaceae**: *Allium* L., *Narcissus* L. **Apiaceae**: *Aegopodium podagraria* L., *Anthriscus* Pers., *Anthriscus sylvestris* (L.) Hoffm., *Daucus carota* L., *Daucus* L., *Dorycnium* Mill., *Heracleum sphondylium* L., *Oenanthe crocata* L., *Pimpinella saxifraga* L., *Smyrnium olusatrum* L., *Torilis japonica* (Houtt.) DC. **Asteraceae**: *Achillea millefolium* L., *Anthemis* L., *Arctium minus* (Hill) Bernh., *Bellis perennis* L., *Centaurea cyanus* L., *Centaurea nigra* L., *Centaurea scabiosa* L., *Cirsium arvense* (L.) Scop., *Cirsium* Mill., *Cirsium vulgare* (Savi) Ten., *Crepis capillaris* (L.) Wallr., *Crepis* L., *Eupatorium cannabinum* L., *Erigeron annuus* (L.) Desf., *Hypochaeris radicata* L., *Leontodon hispidus* L., *Leontodon* L., *Leucanthemum vulgare* Lam., *Picris echioides* L., *Picris hieracioides* L., *Pulicaria dysenterica* (L.) Bernh., *Senecio erucifolius* L., *Senecio jacobaea* L., *Sonchus* L., *Taraxacum* agg. F.H.Wigg., *Taraxacum officinale* agg. F.H.Wigg., *Taraxacum* F.H.Wigg., *Tripleurospermum inodorum* (L.) Sch.Bip. **Brassicaceae**: *Alliaria petiolata* (M.Bieb.) Cavara & Grande, *Arabidopsis thaliana* (L.) Heynh., *Barbarea vulgaris* W.T.Aiton, *Brassica napus* L., *Brassica* L., *Cakile maritima* Scop., *Raphanus raphanistrum* L., *Sinapis alba* L., *Sinapis arvensis* L., *Sinapis* L., *Sisymbrium officinale* (L.) Scop. **Campanulaceae**: *Campanula* L. **Caprifoliaceae**: *Knautia arvensis* (L.) Coult. **Caryophyllaceae**: *Stellaria holostea* L., *Stellaria* L. **Convolvulaceae**: *Convolvulus arvensis* L. **Cornaceae**: *Cornus sanguinea* L. **Cucurbitaceae**: *Bryonia dioica* Bojer **Ericaceae**: *Erica* Tourn. ex L. **Fabaceae**: *Cytisus* Desf., *Laburnum* Fabr., *Lathyrus pratensis* L., *Medicago lupulina* L., *Medicago* L., *Trifolium hybridum* L., *Trifolium pratense* L., *Trifolium repens* L., *Trifolium* Tourn. ex L., *Ulex* L. **Fagaceae**: *Fagus sylvatica* L., *Quercus* L. **Gentianaceae**: *Centaurium erythraea* Rafn **Geraniaceae**: *Geranium* Tourn. ex L. **Lamiaceae**: *Glechoma hederacea* L., *Lamium* L., *Lamium purpureum* L., *Origanum vulgare* L. **Oleaceae**: *Ligustrum vulgare* L. **Onagraceae**: *Chamaenerion angustifolium* (L.) Scop. **Orchidaceae**: *Herminium* L. **Papaveraceae**: *Papaver rhoeas* L. **Plantaginaceae**: *Veronica chamaedrys* L., *Veronica* L. **Polygonaceae**: *Persicaria lapathifolia* (L.) Delarbre **Ranunculaceae**: *Ranunculus bulbosus* L., *Ranunculus ficaria* L., *Ranunculus repens* L., *Ranunculus* L. **Rhamnaceae**: *Rhamnus cathartica* L. **Rosaceae**: *Crataegus monogyna* Jacq., *Filipendula ulmaria* (L.) Maxim., *Fragaria* L., *Potentilla erecta* (L.) Raeusch., *Potentilla* L., *Prunus* L., *Prunus spinosa* L., *Pyrus* L., *Rosa arvensis* Huds., *Rosa* L., *Rubus fruticosus* agg. L., *Rubus idaeus* L., *Rubus* L. **Salicaceae**: *Salix caprea* L., *Salix cinerea* L., *Salix* L. **Sapindaceae**: *Acer campestre* L., *Acer pseudoplatanus* L., *Acer* L., *Aesculus hippocastanum* L. **Viburnaceae**: *Viburnum opulus* L. (Balfour et al. 2022); **Apiaceae**: *Daucus carota* L., *Elaeoselinum* W.D.J.Koch ex DC., *Falcaria vulgaris* Bernh., *Pimpinella cretica* Poir., *Pimpinella major* (L.) Huds., *Pimpinella peregrina* L., *Torilis arvensis* (Huds.) Link **Asphodelaceae**: *Asphodelus aestivus* Brot. **Asteraceae**: *Achillea millefolium* L., *Andryala ragusina* L., *Anthemis arvensis* L., *Anthemis chia* L., *Bellis perennis* L., *Buphthalmum salicifolium* L., *Carduus acanthoides* L., *Calendula arvensis* L., *Centaurea cyanus* L., *Centaurea jacea* L., *Centaurea nigra* L., *Centaurea scabiosa* L., *Centaurea stoebe* L., *Centaurea spinosa* L., *Chamaemelum fuscatum* (Brot.) Vasc., *Chamaemelum nobile* (L.) All., *Cichorium intybus* L., *Cirsium arvense* (L.) Scop., *Cirsium vulgare* (Savi) Ten., *Crepis* L., *Crepis biennis* L., *Crepis capillaris* (L.) Wallr., *Crepis commutata* (Spreng.) Greuter, *Crepis setosa* Haller f., *Echinops ritro* L., *Erigeron annuus* (L.) Desf., *Eupatorium cannabinum* L., *Glebionis coronaria* (L.) Cass. ex Spach, *Hedypnois cretica* (L.) Dum.Cours., *Helichrysum italicum* (Roth) G.Don, *Helichrysum stoechas* (L.) Moench, *Helminthotheca echioides* (L.) Holub, *Hypochaeris achyrophorus* L., *Hypochaeris radicata* L., *Jacobaea vulgaris* Gaertn., *Jurinea humilis* (Desf.) DC., *Leontodon* L., *Leontodon hispidus* L., *Leontodon saxatilis* Lam., *Leontodon tuberosus* L., *Leucanthemopsis alpina* (L.) Heywood, *Leucanthemum vulgare* Lam., *Pentanema ensifolium* (L.) D.Gut.Larr., Santos-Vicente, Anderb., E.Rico & M.M.Mart.Ort., *Pentanema salicinum* (L.) D.Gut.Larr., Santos-Vicente, Anderb., E.Rico & M.M.Mart.Ort., *Phagnalon saxatile* (L.) Cass., *Picris hieracioides* L., *Senecio* L., *Senecio inaequidens* DC., *Sonchus arvensis* L., *Taraxacum* F.H.Wigg., *Taraxacum campylodes* G.E.Haglund, *Taraxacum officinale* F.H.Wigg., *Xeranthemum cylindraceum* Sm. **Boraginaceae**: *Echium plantagineum* L., *Echium vulgare* L. **Brassicaceae**: *Brassica barrelieri* (L.) Janka, *Brassica juncea* (L.) Czern., *Brassica napus* L., *Bunias orientalis* L., *Erysimum pusillum* Bory & Chaub., *Hirschfeldia incana* (L.) Lagr.-Foss., *Raphanus raphanistrum* L., *Rorippa sylvestris* (L.) Besser, *Sisymbrium loeselii* L. **Campanulaceae**: *Jasione crispa* (Pourr.) Samp. **Caprifoliaceae**: *Valeriana officinalis* L. **Cistaceae**: *Cistus albidus* L., *Cistus crispus* L., *Cistus halimifolius* L., *Cistus ladanifer* L., *Cistus monspeliensis* L., *Cistus parviflorus* Lam., *Cistus salviifolius* L., *Helianthemum oelandicum* (L.) Dum.Cours. **Convolvulaceae**: *Convolvulus arvensis* L. **Cupressaceae**: *Juniperus communis* L. **Euphorbiaceae**: *Euphorbia esula* L. **Fabaceae**: *Adenocarpus hispanicus* (Lam.) DC., *Coronilla varia* L., *Cytisus oromediterraneus* Rivas Mart. & al., *Genista acanthoclada* DC., *Genista hirsuta* Vahl, *Lotus corniculatus* L., *Lotus dorycnium* L., *Medicago falcata* L., *Medicago lupulina* L., *Medicago sativa* L., *Melilotus albus* Medik., *Melilotus officinalis* (L.) Lam., *Onobrychis viciifolia* Scop., *Ononis spinosa* L., *Trifolium arvense* L., *Trifolium campestre* Schreb., *Trifolium fragiferum* L., *Trifolium montanum* L., *Trifolium pratense* L., *Trifolium repens* L. **Geraniaceae**: *Geranium* Tourn. ex L., *Geranium pratense* L., *Geranium pyrenaicum* Burm.f. **Hydrangeaceae**: *Philadelphus coronarius* L. **Hypericaceae**: *Hypericum perforatum* L. **Lamiaceae**: *Origanum vulgare* L., *Satureja thymbra* L., *Teucrium capitatum* L., *Thymbra capitata* (L.) Cav., *Thymus praecox* Opiz, *Thymus pulegioides* L., **Linaceae**: *Linum tenuifolium* L. **Malvaceae**: *Malva alcea* L. **Papaveraceae**: *Papaver rhoeas* L. **Plantaginaceae**: *Plantago* L., *Plantago lanceolata* L., *Plantago media* L., *Veronica persica* Poir. **Plumbaginaceae**: *Armeria caespitosa* (Ortega) Boiss. **Primulaceae**: *Primula veris* L. **Ranunculaceae**: *Ranunculus acris* L., *Ranunculus polyanthemos* L. **Resedaceae**: *Reseda lutea* L. **Rosaceae**: *Fragaria viridis* Weston, *Potentilla erecta* (L.) Raeusch., *Potentilla reptans* L., *Rosa canina* L., *Rubus* L. (Lanuza et al. 2025); **Asteraceae**: *Centaurea iberica* Trev. ex Spreng., *Cichorium intybus* L., *Echinops orientalis* Trautv. **Brassicaceae**: *Brassica* L., *Sinapis arvensis* L., *Sisymbrium altissimum* L. **Fabaceae**: *Melilotus officinalis* (L.) Lam., *Trifolium repens* L., *Trigonella spicata* Sibth. & Sm. **Papaveraceae**: *Glaucium leiocarpum* Boiss. **Ranunculaceae**: *Ranunculus marginatus* d’Urv. **Rosaceae**: Rubus canescens var. glabratus (Godr.) Davis & Meikle.

### Andrena (Micrandrena) floricola

Eversmann, 1852

97D12EB4-5971-51E0-8612-8F6E83214AFE

#### Materials

- **Type status:** Other material. **Occurrence:** recordedBy: Fatih Dikmen; individualCount: 1; sex: female; occurrenceID: 05D9BE50-AB4A-5FDD-8542-F4DD9E0487D5; **Location:** countryCode: TR; stateProvince: Mersin; county: Mut; locality: Güzlek Plateau; verbatimElevation: 1414 m; verbatimLatitude: 36.654; verbatimLongitude: 33.107; **Identification:** identifiedBy: Thomas J. Wood; **Event:** eventDate: 04-06-2009; **Record Level:** collectionCode: ZMUI

#### Distribution

Austria, Belarus, Belgium, Bosnia and Herzegovina, Czechia, France, Georgia, Germany, Great Britain, Iran, Lithuania, Netherlands, Poland, Russia, Spain, Switzerland and Türkiye (Gusenleitner and Schwarz 2002, Wood and Monfared 2022, Ascher and Pickering 2025).

##### Distribution in Türkiye

**Figure 31.**
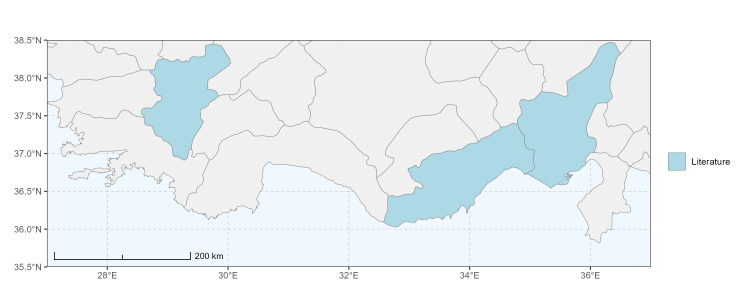
Distribution of *A. floricola* in the Mediterranean Region of Türkiye.

Adana, Amasya, Ardahan, Balıkesir (Ayvalık), Diyarbakır, Erzurum (Tortum), Eskişehir, Konya (Sarayönü), Mersin (Gülek) (Warncke 1974); Hakkari, Denizli (Karakurt) (Dardón et al. 2014); Mersin (this study) (Fig. 31).

#### Flower records

**Apiaceae**: *Foeniculum vulgare* Mill., *Turgenia* Hoffm. **Fabaceae**: *Melilotus officinalis* (L.) Lam. **Ranunculaceae**: *Delphinium* Tourn. ex L. (Khodarahmi Ghahnavieh and Monfared 2019); **Asteraceae**: *Achillea millefolium* L., *Lactuca serriola* L., *Senecio vernalis* Waldst. & Kit., *Tripleurospermum maritimum* (L.) W.D.J.Koch **Brassicaceae**: *Sisymbrium loeselii* L. (Lanuza et al. 2025); **Brassicaceae**: *Lepidium draba* L.

### Andrena (Holandrena) forsterella

Osytshnjuk, 1978

2EC8BD1D-7979-5291-8A3F-92784854B5B3

#### Materials

- **Type status:** Other material. **Occurrence:** recordedBy: Fatih Dikmen; individualCount: 1; sex: female; occurrenceID: 25855E71-9352-5E89-95B9-D1CA6CC84155; **Location:** countryCode: TR; stateProvince: Adana; locality: Kadirli-Andırın road; verbatimElevation: 224 m; verbatimLatitude: 37.422; verbatimLongitude: 36.281; **Identification:** identifiedBy: Thomas J. Wood; **Event:** eventDate: 10-07-2008; **Record Level:** collectionCode: ZMUI
- **Type status:** Other material. **Occurrence:** recordedBy: Fatih Dikmen; individualCount: 1; sex: female; occurrenceID: 33433EBD-3354-534B-9321-6D31ED969E08; **Location:** countryCode: TR; stateProvince: Kahramanmaraş; locality: Göksun road; verbatimElevation: 901 m; verbatimLatitude: 37.727; verbatimLongitude: 36.693; **Identification:** identifiedBy: Thomas J. Wood; **Event:** eventDate: 02-07-2009; **Record Level:** collectionCode: ZMUI
- **Type status:** Other material. **Occurrence:** recordedBy: Fatih Dikmen; individualCount: 1; sex: male; occurrenceID: 19A62CB3-680B-5816-BB39-41EAB9B21DCA; **Location:** countryCode: TR; stateProvince: Muğla; county: Datça; locality: Ballık hill; verbatimElevation: 0 m; verbatimLatitude: 36.808; verbatimLongitude: 27.901; **Identification:** identifiedBy: Thomas J. Wood; **Event:** eventDate: 04-04-2009; **Record Level:** collectionCode: ZMUI
- **Type status:** Other material. **Occurrence:** recordedBy: Fatih Dikmen; individualCount: 1; sex: female; occurrenceID: 58477EDB-1C91-53D4-9596-30E846A40B22; **Location:** countryCode: TR; stateProvince: Hatay; locality: between Yayladağ and Samandağ; verbatimElevation: 450 m; verbatimLatitude: 35.923; verbatimLongitude: 36.054; **Identification:** identifiedBy: Thomas J. Wood; **Event:** eventDate: 27-04-2008; **Record Level:** collectionCode: ZMUI

#### Distribution

Azerbaijan, Bulgaria, Croatia, Cyprus, Greece, Iran, Israel and the West Bank, Italy, Lebanon, Liechtenstein, Macedonia and Türkiye (Schönitzer et al. 1995, Wood et al. 2020, Wood 2021b, Pisanty et al. 2022, Ascher and Pickering 2025).

##### Distribution in Türkiye

**Figure 32.**
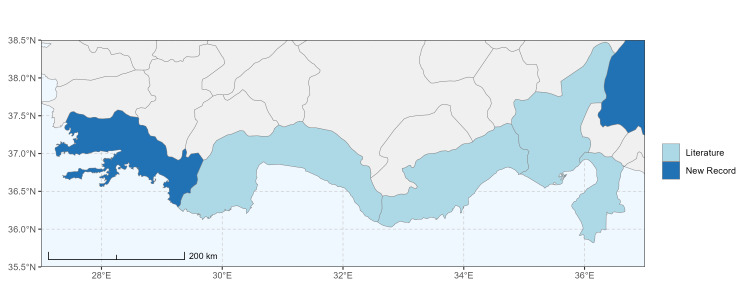
Distribution of *A. forsterella* in the Mediterranean Region of Türkiye.

Erzurum, Muş, Tunceli (Özbek 1976); Antalya (Akseki), Diyarbakır, Konya (Seydişehir), Samsun, Şanlıurfa, Tokat (Niksar) (Schönitzer et al. 1995); Adana (Ceyhan), Ankara (Şereflikoçhisar), Balıkesir (Ayvalık), Bursa (Karacabey), Hatay (Topboğazı), Iğdır, İstanbul (Florya), Konya (Beyşehir), Mersin (Mut, Namrum) (Schuberth 1995, Hazır 2010); Antalya, Antep, Diyarbakır, Konya, Mersin (Hazır et al. 2014); Adana, Hatay, Kahramanmaraş, Muğla (this study) (Fig. 32).

#### Taxon discussion

In this study, *Andrena wilhelmi* and *Andrena forsterella* are treated as conspecific. We follow the taxonomic classification proposed by Wood (2026), wherein *A. wilhelmi* is considered a synonym of *A. forsterella*.

#### Flower records

**Asteraceae**: Centaurea solstitialis subsp. carneola (Boiss.) Wagenitz, *Echinops orientalis* Trautv., *Glebionis segetum* (L.) Fourr.

### Andrena (Chrysandrena) fulvago

(Christ, 1791)

7B3DCE5E-C004-5E63-9299-007BEDF41DB2

#### Materials

- **Type status:** Other material. **Occurrence:** recordedBy: Fatih Dikmen; individualCount: 3; sex: female; occurrenceID: 89EAE04E-5AB1-540C-99B2-D217C393D871; **Location:** countryCode: TR; stateProvince: Kahramanmaraş; county: Andırın; locality: Çokak village exit; verbatimElevation: 1362 m; verbatimLatitude: 37.76; verbatimLongitude: 36.35; **Identification:** identifiedBy: Thomas J. Wood; **Event:** eventDate: 18-07-2009; **Record Level:** collectionCode: ZMUI

#### Distribution

Afghanistan, Albania, Armania, Austria, Belarus, Belgium, Bosnia and Herzegovina, Bulgaria, Croatia, Czechia, Denmark, Finland, France, Georgia, Germany, Great Britain, Greece, Iran, Italy, Kazakhstan, Latvia, Lithuania, Luxembourg, Montenegro, Netherlands, Norway, Poland, Portugal, Romania, Russia, Slovakia, Spain, Sweden, Switzerland, Türkiye and Ukraine (Wood and Monfared 2022, Wood 2024, Ascher and Pickering 2025).

##### Distribution in Türkiye

**Figure 33.**
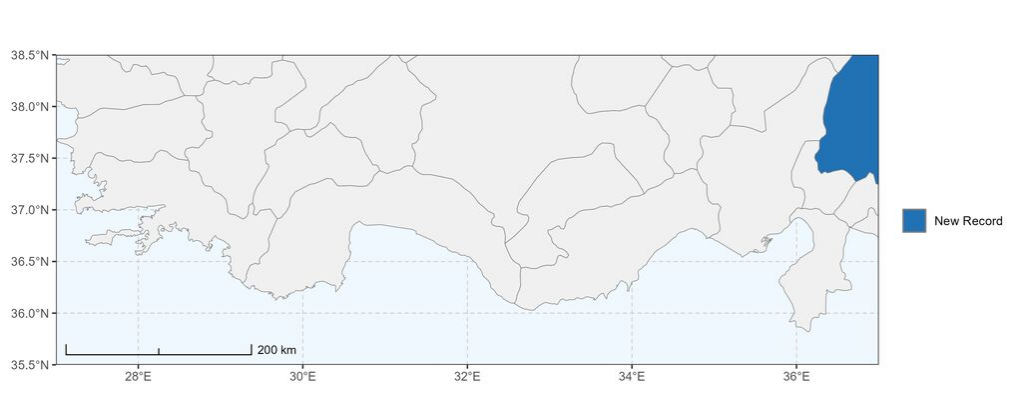
Distribution of *A. fulvago* in the Mediterranean Region of Türkiye.

Bolu (Abant) (Warncke 1969); İstanbul (Belgrad) (Warncke 1974); Ankara, Kırklareli (Hazır et al. 2014); Kahramanmaraş (this study) (Fig. 29D-F and Fig. 33).

#### Flower records

**Asteraceae**: *Taraxacum* agg. F.H.Wigg. (Balfour et al. 2022); **Asteraceae**: *Crepis* L., *Crepis capillaris* (L.) Wallr., *Crepis biennis* L., *Hypochaeris radicata* L., *Lapsana communis* L., *Leontodon hispidus* L., *Picris hieracioides* L., *Pilosella* Hill, *Pilosella officinarum* F.W.Schultz & Sch.Bip., *Sonchus oleraceus* L. **Geraniaceae**: *Geranium* Tourn. ex L., *Geranium pyrenaicum* Burm.f., **Ranunculaceae**: *Ranunculus acris* L. (Lanuza et al. 2025); **Asteraceae**: *Cichorium intybus* L.

### Andrena (Ulandrena) fulvitarsis

Brullé, 1833

73E3755F-8906-5D48-BA6A-AADC517341DA

#### Materials

- **Type status:** Other material. **Occurrence:** recordedBy: Fatih Dikmen; individualCount: 1; sex: female; occurrenceID: 1FAA3984-EF9D-5A45-8F4D-E2578C3356CC; **Location:** countryCode: TR; stateProvince: Burdur; locality: Ağlasun-Antakya road; verbatimElevation: 1048 m; verbatimLatitude: 37.617; verbatimLongitude: 30.522; **Identification:** identifiedBy: Thomas J. Wood; **Event:** eventDate: 29-05-2009; **Record Level:** collectionCode: ZMUI

#### Distribution

Albania, Bulgaria, Croatia, Greece, Iran, Israel and the West Bank, Italy, Jordan, Lebanon, North Macedonia, Slovenia, Türkiye and Ukraine (Gusenleitner and Schwarz 2002, Wood et al. 2020, Wood and Monfared 2022, Wood et al. 2024).

##### Distribution in Türkiye

**Figure 34.**
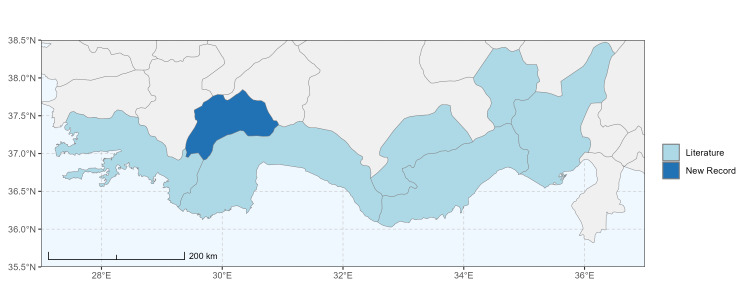
Distribution of *A. fulvitarsis* in the Mediterranean Region of Türkiye.

Adana, Ankara, Antalya (Side), Çanakkale (Truva), Diyarbakır, Edirne, İstanbul (Belgrad Ormanı, Büyükdere), Karaman (Madenşehri), Kayseri (Yeşilhisar), Mersin (Anamur, Gilindire, Gülek), Muğla (Marmaris), Niğde (Ulukışla), Urfa (Birecik) (Warncke 1974); Adana, Ankara, Antalya, Antep, Aydın, İzmir, Karabük, Karaman, Kırşehir, Konya, Kütahya, Manisa (Hazır et al. 2014); Burdur (this study) (Fig. 34).

#### Flower records

**Asteraceae**: *Crepis sancta* (L.) Bornm., *Tripleurospermum* Sch.Bip. (Lanuza et al. 2025); **Asteraceae**: *Anthemis pseudocotula* Boiss.

### Andrena (Melanapis) fuscosa

Erichson, 1835

957A9921-FC68-5822-8347-17A583553098

#### Materials

- **Type status:** Other material. **Occurrence:** recordedBy: Fatih Dikmen; individualCount: 1; sex: female; occurrenceID: 8819D90A-38CC-5550-841D-DCFDF1D47BCD; **Location:** countryCode: TR; stateProvince: Antalya; county: Gazipaşa; verbatimElevation: 149 m; verbatimLatitude: 36.192; verbatimLongitude: 32.435; **Identification:** identifiedBy: Thomas J. Wood; **Event:** eventDate: 08-06-2009; **Record Level:** collectionCode: ZMUI

#### Distribution

Afghanistan, Algeria, Austria, Croatia, Cyprus, Dağıstan, Egypt, France, Greece, Hungary, India, Iran, Israel and the West Bank, Italy, Kazakhstan, Libya, Morocco, Moritanya, Pakistan, Poland, Romania, Slovakia, Spain, Switzerland, Tajikistan, Tunisia, Türkiye, Ukraine and Uzbekistan (Gusenleitner and Schwarz 2002, Osytshnjuk et al. 2008, Wood and Monfared 2022, Wood 2023b, Wood 2024, Wood 2025, Ascher and Pickering 2025).

##### Distribution in Türkiye

**Figure 35.**
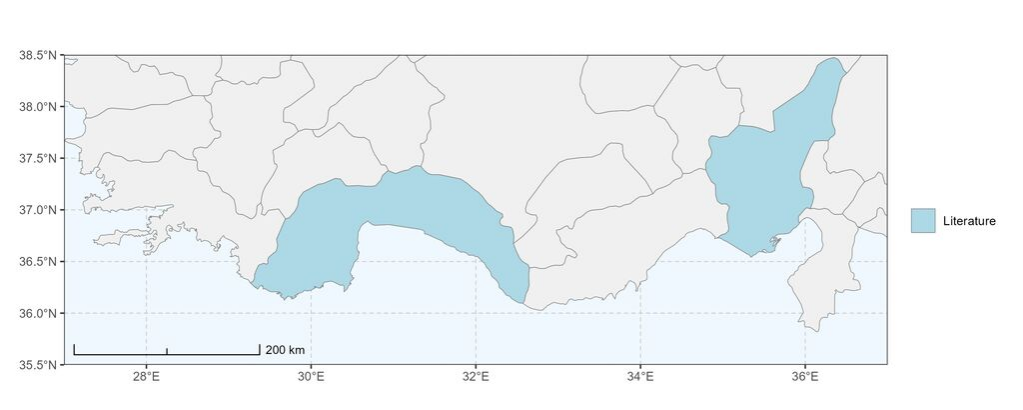
Distribution of *A. fuscosa* in the Mediterranean Region of Türkiye.

Adana (Ceyhan, Karataş), Amanos Mountains, Ankara, Balıkesir (Ayvalık), Sakarya (Adapazarı) (Warncke 1966); Aydın (Kuşadası), Bilecik (Warncke 1969); Erzurum (İspir, Horasan) (Özbek 1976); Antep, Konya (Hazır 2010); Antalya, Antep, Aydın, Erzurum, Konya (Hazır et al. 2014); Antalya (this study) (Fig. 35).

#### Flower records

**Alliaceae**: *Allium cepa* L. **Amaranthaceae**: *Amaranthus* L. **Asteraceae**: *Centaurea virgata* Lam. **Apiaceae**: *Petroselinum* Hill. **Brassicaceae**: *Brassica napus* L., *Descurainia sophia* (L.) Webb ex Prantl **Boraginaceae**: Anchusa azurea var. azurea Mill. **Chenopodiaceae**: *Chenopodium* L. **Papaveraceae**: *Papaver* L. **Primulaceae**: *Lysimachia arvensis* (L.) U.Manns & Anderb. (Khodarahmi Ghahnavieh and Monfared 2019); **Apiaceae**: *Pimpinella peregrina* L. **Brassicaceae**: *Hirschfeldia incana* (L.) Lagr.-Foss. **Lamiaceae**: *Thymbra capitata* (L.) Cav. (Lanuza et al. 2025); **Brassicaceae**: *Hirschfeldia incana* (L.) Lagr.-Foss.

### Andrena (Simandrena) gasparella

Patiny, 1998

650FEE66-BA2D-594C-B574-2D3D6ACD3B78

#### Materials

- **Type status:** Other material. **Occurrence:** recordedBy: Fatih Dikmen; individualCount: 3; sex: female; occurrenceID: 1DF6EDF3-6960-53BB-BD92-CA54A4AA6149; **Location:** countryCode: TR; stateProvince: Antalya; locality: Akseki-Konya road; verbatimElevation: 1496 m; verbatimLatitude: 37.14; verbatimLongitude: 31.875; **Identification:** identifiedBy: Thomas J. Wood; **Event:** eventDate: 09-06-2009; **Record Level:** collectionCode: ZMUI

#### Distribution

Iran and Türkiye (Gusenleitner and Schwarz 2002, Khodarahmi Ghahnavieh and Monfared 2019, Wood and Monfared 2022).

##### Distribution in Türkiye

**Figure 36.**
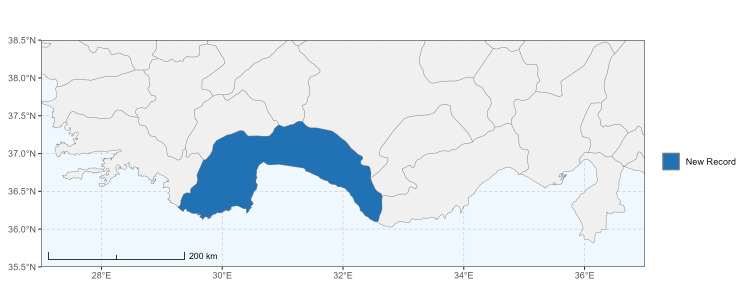
Distribution of *A. gasparella* in the Mediterranean Region of Türkiye.

**Figure 37.**
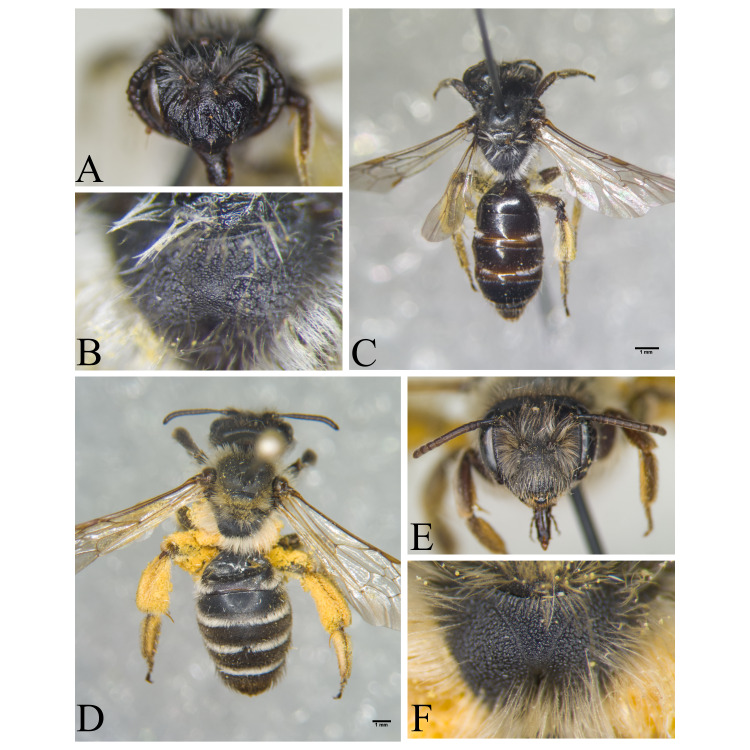
*Andrena gasparella*, female. Head, frontal view (A); propodeum, dorsal view (B); habitus, dorsal view (C). Andrena hungarica ssp. macroura, female. Habitus, dorsal view (D), head, frontal view (E), propodeum, dorsal view (F).

Konya (Akşehir) (Patiny 1998); Antalya (this study) (Fig. 36 and Fig. 37A-C).

#### Flower records

**Apiaceae**: *Foeniculum vulgare* Mill., *Turgenia* Hoffm. **Brassicaceae**: *Brassica napus* L., *Lepidium draba* L., **Fabaceae**: *Papaver* L. **Ranunculaceae**: *Delphinium* Tourn. ex L. (Khodarahmi Ghahnavieh and Monfared 2019); **Brassicaceae**: *Isatis* L.

### Andrena (Chrysandrena) glandaria

Warncke, 1975

B0B782E5-2D30-5B07-A018-8E0724DFF012

#### Materials

- **Type status:** Other material. **Occurrence:** recordedBy: Fatih Dikmen; individualCount: 9; sex: female; occurrenceID: 7750ECFB-E3AD-5A1B-81C5-1FC0DD122C2D; **Location:** countryCode: TR; stateProvince: Adana; county: Seyhan; locality: DSI facilities; verbatimElevation: 91 m; verbatimLatitude: 37; verbatimLongitude: 35.33; **Identification:** identifiedBy: Thomas J. Wood; **Event:** eventDate: 23-05-2009; **Record Level:** collectionCode: ZMUI
- **Type status:** Other material. **Occurrence:** recordedBy: Fatih Dikmen; individualCount: 1; sex: male; occurrenceID: B3067328-4352-58CE-970B-1607C62E3B68; **Location:** countryCode: TR; stateProvince: Adana; county: Seyhan; locality: DSI facilities; verbatimElevation: 91 m; verbatimLatitude: 37; verbatimLongitude: 35.33; **Identification:** identifiedBy: Thomas J. Wood; **Event:** eventDate: 23-05-2009; **Record Level:** collectionCode: ZMUI
- **Type status:** Other material. **Occurrence:** recordedBy: Fatih Dikmen; individualCount: 19; sex: female; occurrenceID: 0255721B-3973-557D-9E26-31EFE8949FDB; **Location:** countryCode: TR; stateProvince: Adana; locality: Çukurova University, Balcalı Campus; verbatimElevation: 83 m; verbatimLatitude: 37.049; verbatimLongitude: 35.357; **Identification:** identifiedBy: Thomas J. Wood; **Event:** eventDate: 23-05-2009; **Record Level:** collectionCode: ZMUI
- **Type status:** Other material. **Occurrence:** recordedBy: Fatih Dikmen; individualCount: 5; sex: male; occurrenceID: 935AD164-A264-538F-922F-2B4F0B3C04C4; **Location:** countryCode: TR; stateProvince: Adana; locality: Çukurova University, Balcalı Campus; verbatimElevation: 83 m; verbatimLatitude: 37.049; verbatimLongitude: 35.357; **Identification:** identifiedBy: Thomas J. Wood; **Event:** eventDate: 23-05-2009; **Record Level:** collectionCode: ZMUI
- **Type status:** Other material. **Occurrence:** recordedBy: Fatih Dikmen; individualCount: 1; sex: female; occurrenceID: 1B3C8523-A6E4-5B5E-ADB0-1EC5A977CA48; **Location:** countryCode: TR; stateProvince: Mersin; locality: Mut-Silifke road, Sarıkavak turn; verbatimElevation: 223 m; verbatimLatitude: 36.503; verbatimLongitude: 33.585; **Identification:** identifiedBy: Thomas J. Wood; **Event:** eventDate: 22-05-2009; **Record Level:** collectionCode: ZMUI
- **Type status:** Other material. **Occurrence:** recordedBy: Fatih Dikmen; individualCount: 1; sex: female; occurrenceID: F19378B5-B089-5309-A53A-38379F90CBD3; **Location:** countryCode: TR; stateProvince: Hatay; locality: Yayladağ; verbatimElevation: 423 m; verbatimLatitude: 35.908; verbatimLongitude: 36.078; **Identification:** identifiedBy: Thomas J. Wood; **Event:** eventDate: 22-03-2009; **Record Level:** collectionCode: ZMUI
- **Type status:** Other material. **Occurrence:** recordedBy: Fatih Dikmen; individualCount: 1; sex: male; occurrenceID: AD6EF303-72F8-575D-9C34-179E76ED6081; **Location:** countryCode: TR; stateProvince: Muğla; county: Marmaris; locality: Bördübet road; verbatimElevation: 195 m; verbatimLatitude: 36.857; verbatimLongitude: 28.126; **Identification:** identifiedBy: Thomas J. Wood; **Event:** eventDate: 04-04-2009; **Record Level:** collectionCode: ZMUI
- **Type status:** Other material. **Occurrence:** recordedBy: Fatih Dikmen; individualCount: 1; sex: female; occurrenceID: 8D500D41-BC16-5E13-8327-D8B7126E7F5D; **Location:** countryCode: TR; stateProvince: Adana; locality: Çukurova University, Balcalı Campus; verbatimElevation: 145 m; verbatimLatitude: 37.065; verbatimLongitude: 35.366; **Identification:** identifiedBy: Thomas J. Wood; **Event:** eventDate: 20-03-2009; **Record Level:** collectionCode: ZMUI
- **Type status:** Other material. **Occurrence:** recordedBy: Fatih Dikmen; individualCount: 2; sex: female; occurrenceID: 8E2C97E4-E4FA-523C-BF38-7AED29027374; **Location:** countryCode: TR; stateProvince: Adana; locality: Çukurova University, Balcalı Campus; verbatimElevation: 83 m; verbatimLatitude: 37.049; verbatimLongitude: 35.357; **Identification:** identifiedBy: Thomas J. Wood; **Event:** eventDate: 20-03-2009; **Record Level:** collectionCode: ZMUI

#### Diagnosis

Greece, Iraq, Israel and the West Bank, Syria and Türkiye (Gusenleitner and Schwarz 2002).

##### Distribution in Türkiye

**Figure 38.**
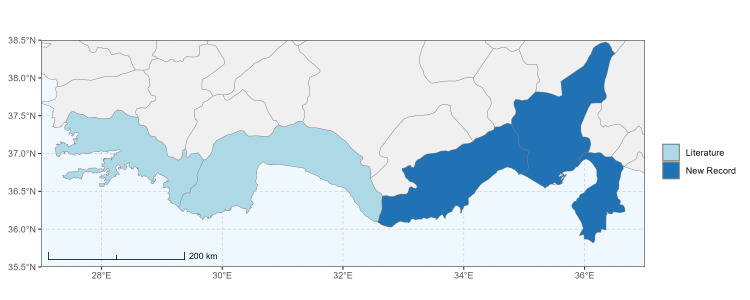
Distribution of *A. glandaria* in the Mediterranean Region of Türkiye.

Antalya (Warncke 1974); Urfa (Blank and Kraus 1994); Aydın, Isparta (Gelendost), Muğla (Hazır 2010, Hazır et al. 2014); Adana, Hatay, Mersin, Muğla (this study) (Fig. 38).

#### Flower records

**Asteraceae**: *Calendula arvensis* L., *Crepis* L., *Crepis foetida* L., Crepis foetida subsp. rhoeadifolia (M.Bieb.) Čelak., *Senecio vernalis* Waldst. & Kit. **Brassicaceae**: *Hirschfeldia incana* (L.) Lagr.-Foss.

### Andrena (Chrysandrena) hesperia

Smith, 1853

1DAE567E-AA56-568F-ABC5-5EC640683DFC

#### Materials

- **Type status:** Other material. **Occurrence:** recordedBy: Fatih Dikmen; individualCount: 2; sex: male; occurrenceID: 9E7F9490-5173-5AD1-BC1C-0745A58D96E5; **Location:** countryCode: TR; stateProvince: Adana; locality: Çukurova University, Balcalı Campus; verbatimElevation: 83 m; verbatimLatitude: 37.049; verbatimLongitude: 35.357; **Identification:** identifiedBy: Thomas J. Wood; **Event:** eventDate: 20-03-2009; **Record Level:** collectionCode: ZMUI
- **Type status:** Other material. **Occurrence:** recordedBy: Fatih Dikmen; individualCount: 1; sex: male; occurrenceID: 2E5A1FD8-B36E-5BAD-A008-F89054DCB34C; **Location:** countryCode: TR; stateProvince: Hatay; locality: Belen Pass; verbatimElevation: 274 m; verbatimLatitude: 36.46; verbatimLongitude: 36.279; **Identification:** identifiedBy: Thomas J. Wood; **Event:** eventDate: 21-03-2009; **Record Level:** collectionCode: ZMUI

#### Distribution

Algeria, Austria, Cyprus, Croatia, Egypt, France, Greece, Israel and the West Bank, Italy, Morocco, Portugal, Slovenia, Spain, Switzerland, Tajikistan, Tunisia, Turkmenistan, Türkiye and Ukraine (Gusenleitner and Schwarz 2002, Osytshnjuk et al. 2005, Wood and Monfared 2022, Ascher and Pickering 2025).

##### Distribution in Türkiye

**Figure 39.**
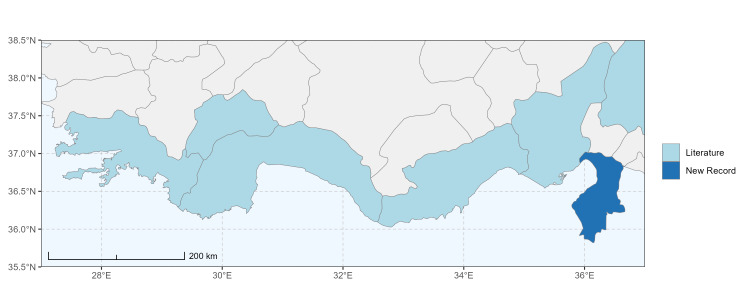
Distribution of *A. hesperia* in the Mediterranean Region of Türkiye.

Adana, Balıkesir (Ayvalık), Konya (Warncke 1966); Ankara, Antalya (Akseki, Side), Bursa, Denizli (Acıgöl, Pamukkale), Diyarbakır, İstanbul (Büyükçekmece, Büyükdere), Manisa, Mersin (Tarsus) (Warncke 1974); Erzurum (İspir) (Özbek 1976); Adana, Ankara, Antalya, Aydın, Çanakkale, Karaman, Manisa, Maraş, Muğla (Hazır 2010, Hazır et al. 2014); Adana, Hatay (this study) (Fig. 39).

#### Flower records

**Asteraceae**: *Centaurea* L., *Tragopogon* L. **Fabaceae**: *Medicago sativa* L., *Melilotus officinalis* (L.) Lam. (Khodarahmi Ghahnavieh and Monfared 2019); **Asteraceae**: *Bellis annua* L., *Calendula arvensis* L., *Hypochaeris achyrophorus* L., *Leontodon tuberosus* L. (Lanuza et al. 2025); **Asteraceae**: *Calendula arvensis* L., *Crepis sancta* (L.) Bornm.

### Andrena (Chlorandrena) humabilis

Warncke, 1965

D969A364-B01C-56FD-9CA3-484620170331

#### Materials

- **Type status:** Other material. **Occurrence:** recordedBy: Fatih Dikmen; individualCount: 1; sex: male; occurrenceID: 0093D167-4000-5872-83ED-5878661139DA; **Location:** countryCode: TR; stateProvince: Osmaniye; locality: Karatepe National Park; verbatimElevation: 325 m; verbatimLatitude: 37.26; verbatimLongitude: 36.219; **Identification:** identifiedBy: Thomas J. Wood; **Event:** eventDate: 20-03-2009; **Record Level:** collectionCode: ZMUI
- **Type status:** Other material. **Occurrence:** recordedBy: Fatih Dikmen; individualCount: 2; sex: female; occurrenceID: C6D1FC06-2B03-5846-89A9-CB958DA5B558; **Location:** countryCode: TR; stateProvince: Hatay; locality: Yayladağ; verbatimElevation: 423 m; verbatimLatitude: 35.908; verbatimLongitude: 36.078; **Identification:** identifiedBy: Thomas J. Wood; **Event:** eventDate: 22-03-2009; **Record Level:** collectionCode: ZMUI
- **Type status:** Other material. **Occurrence:** recordedBy: Fatih Dikmen; individualCount: 1; sex: female; occurrenceID: EB510070-246E-5C5F-BC48-406A56EBF6A2; **Location:** countryCode: TR; stateProvince: Muğla; county: Marmaris; verbatimElevation: 156 m; verbatimLatitude: 36.87; verbatimLongitude: 28.19; **Identification:** identifiedBy: Thomas J. Wood; **Event:** eventDate: 02-04-2009; **Record Level:** collectionCode: ZMUI
- **Type status:** Other material. **Occurrence:** recordedBy: Fatih Dikmen; individualCount: 2; sex: female; occurrenceID: AF30DB1B-FE16-5531-BBBA-810B2D50DB37; **Location:** countryCode: TR; stateProvince: Adana; locality: Çukurova University, Balcalı Campus; verbatimElevation: 83 m; verbatimLatitude: 37.049; verbatimLongitude: 35.357; **Identification:** identifiedBy: Thomas J. Wood; **Event:** eventDate: 20-03-2009; **Record Level:** collectionCode: ZMUI

#### Distribution

Bulgaria, Greece and Türkiye (Gusenleitner and Schwarz 2002).

##### Distribution in Türkiye

**Figure 40.**
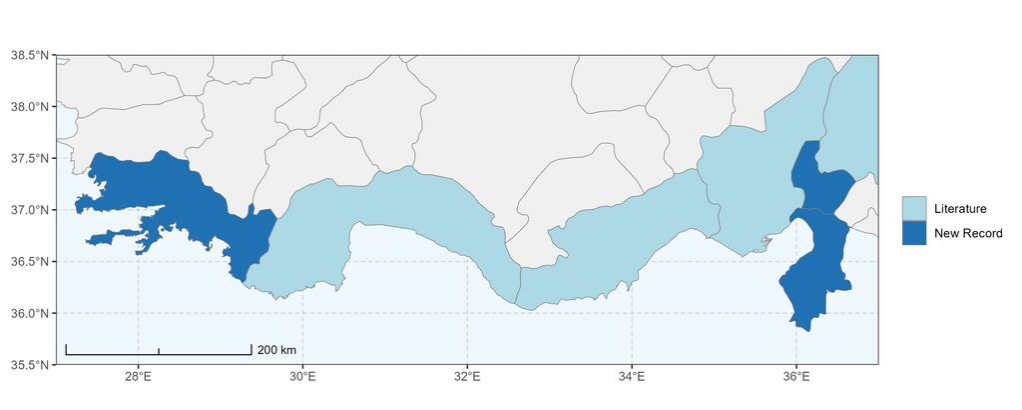
Distribution of *A. humabilis* in the Mediterranean Region of Türkiye.

Adana, Balıkesir (Ayvalık, Bigadiç), Bursa (Mustafakemalpaşa, Karacabey), Çanakkale (Truba), Diyarbakır, İstanbul (Büyükdere), Mersin (Gülek) (Warncke 1974); Adana, Antalya, Aydın, Maraş (Hazır 2010, Hazır et al. 2014); Adana, Hatay, Muğla, Osmaniye (this study) (Fig. 40).

#### Flower records

**Asteraceae**: *Bellis perennis* L., *Senecio vernalis* Waldst. & Kit., *Crepis sancta* (L.) Bornm., *Calendula arvensis* L.

### Andrena (Melandrena) hungarica macroura

Warncke, 1975

BF835D3A-5455-53E5-A183-8E9C6D98CC04

#### Materials

- **Type status:** Other material. **Occurrence:** recordedBy: Fatih Dikmen; individualCount: 1; sex: male; occurrenceID: 41035E65-36C1-59EE-AFED-553DEEDAEB67; **Location:** countryCode: TR; stateProvince: Muğla; county: Datça; verbatimElevation: 0 m; verbatimLatitude: 36.802; verbatimLongitude: 27.866; **Identification:** identifiedBy: Thomas J. Wood; **Event:** eventDate: 04-04-2009; **Record Level:** collectionCode: ZMUI

#### Distribution

The taxonomic situation is complex and confused. True *A. hungarica* Friese, 1887 is probably restricted to the Pannonian Basin (Austria, Slovakia, Hungary, Serbia), with A. hungarica ssp. macroura seemingly restricted to Türkiye and A. hungarica ssp. khursiensis Osytshnjuk, 1994 is a junior synonym of *Andrena quadimaculata* Friese, 1921 (TJW, in press). The presence of A. hungarica ssp. macroura in the coastal region of Muğla is perplexing, as it has previously only been recorded in central Türkiye; further study of this taxon is required.

##### Distribution in Türkiye

**Figure 41.**
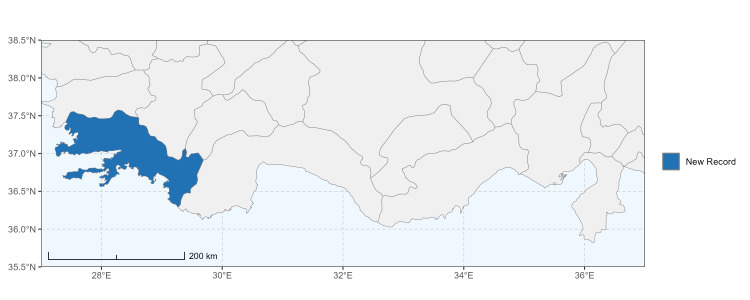
Distribution of A. hungarica ssp. macroura in the Mediterranean Region of Türkiye.

Ankara, Erzurum, Sivas (Gürün) (Warncke 1974); Muğla (this study) (Fig. 37D-F and Fig. 41).

#### Flower records

**Cistaceae**: *Cistus* L.

### Andrena (Fuscandrena) iliaca

Warncke, 1969

8DC17170-D7E0-5569-86CA-865586D0F778

#### Materials

- **Type status:** Other material. **Occurrence:** recordedBy: Fatih Dikmen; individualCount: 1; sex: female; occurrenceID: C1C9384E-D7F3-50CC-921A-CA5C0CDF2CFD; **Location:** countryCode: TR; stateProvince: Hatay; locality: Belen Pass; verbatimElevation: 274 m; verbatimLatitude: 36.46; verbatimLongitude: 36.279; **Identification:** identifiedBy: Thomas J. Wood; **Event:** eventDate: 21-03-2009; **Record Level:** collectionCode: ZMUI
- **Type status:** Other material. **Occurrence:** recordedBy: Fatih Dikmen; individualCount: 5; sex: male; occurrenceID: 9F1AD1D6-A7D3-5E39-98C9-D9AFF5311EDD; **Location:** countryCode: TR; stateProvince: Hatay; locality: Belen Pass; verbatimElevation: 274 m; verbatimLatitude: 36.46; verbatimLongitude: 36.279; **Identification:** identifiedBy: Thomas J. Wood; **Event:** eventDate: 21-03-2009; **Record Level:** collectionCode: ZMUI

#### Distribution

Iran, Israel and the West Bank, Jordan and Türkiye (Gusenleitner and Schwarz 2002, Ascher and Pickering 2025).

##### Distribution in Türkiye

**Figure 42.**
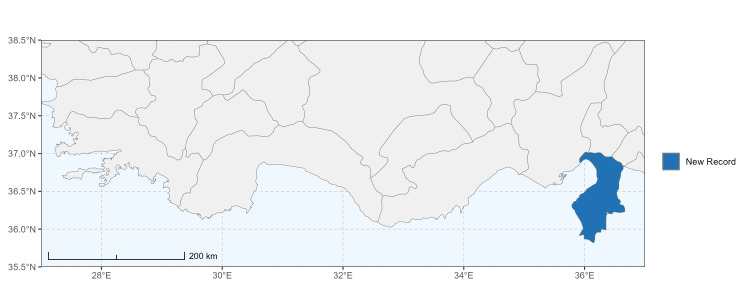
Distribution of *A. iliaca* in the Mediterranean Region of Türkiye.

**Figure 43.**
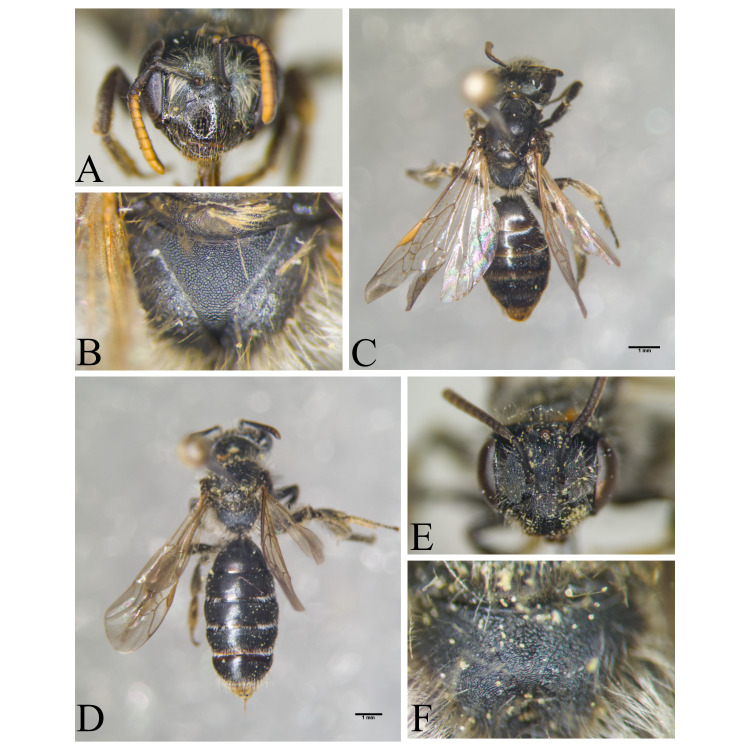
*Andrena iliaca*, female. Head, frontal view (A); propodeum, dorsal view (B); habitus, dorsal view (C). *Andrena immaculata*, female. Habitus, dorsal view (D), head, frontal view (E), propodeum, dorsal view (F).

Diyarbakır (Hazır et al. 2012, Hazır et al. 2014); Hatay (this study) (Fig. 42 and Fig. 43A-C).

#### Flower records

**Brassicaceae**: *Sisymbrium* L.

### Andrena (Micrandrena) immaculata

Warncke, 1975

A641263C-E5FA-5FB6-B60E-50A9192C992C

#### Materials

- **Type status:** Other material. **Occurrence:** recordedBy: Fatih Dikmen; individualCount: 1; sex: female; occurrenceID: E7714C29-77BA-5A62-8A8E-FF0E16779B1C; **Location:** countryCode: TR; stateProvince: Antalya; locality: Burdur road, Korkuteli turn; verbatimElevation: 783 m; verbatimLatitude: 37.313; verbatimLongitude: 30.49; **Identification:** identifiedBy: Thomas J. Wood; **Event:** eventDate: 29-05-2009; **Record Level:** collectionCode: ZMUI

#### Distribution

Cyprus, Israel and the West Bank, Syria and Türkiye (Gusenleitner and Schwarz 2002, Ascher and Pickering 2025, TJW unpublished data).

##### Distribution in Türkiye

**Figure 44.**
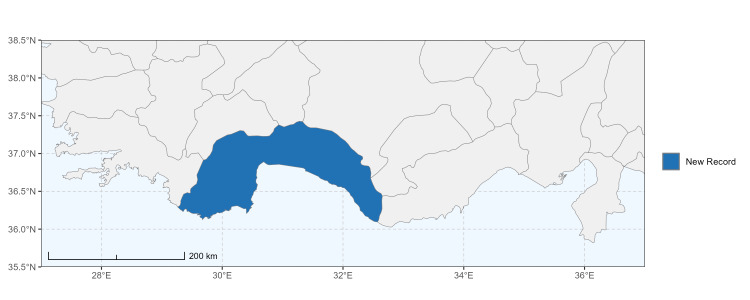
Distribution of *A. immaculata* in the Mediterranean Region of Türkiye.

Ankara (Şereflikoçhisar), Muş (Warncke 1974); Antalya (this study) (Fig. 44).

#### Flower records

**Brassicaceae**: *Hirschfeldia incana* (L.) Lagr.-Foss.

### Andrena (Ulandrena) isabellina

Warncke, 1969

C0D1CBC3-5789-58D1-8DFD-95B12E8D00D4

#### Materials

- **Type status:** Other material. **Occurrence:** recordedBy: Fatih Dikmen; individualCount: 8; sex: male; occurrenceID: 267D525D-18FB-5DCA-9D6C-C73BEB6ABBD4; **Location:** countryCode: TR; stateProvince: Hatay; locality: Belen Pass; verbatimElevation: 274 m; verbatimLatitude: 36.46; verbatimLongitude: 36.279; **Identification:** identifiedBy: Thomas J. Wood; **Event:** eventDate: 21-03-2009; **Record Level:** collectionCode: ZMUI

#### Distribution

Iran, Israel and the West Bank, Jordan, Lebanon and Türkiye (Gusenleitner and Schwarz 2002, Ascher and Pickering 2025).

##### Distribution in Türkiye

**Figure 45.**
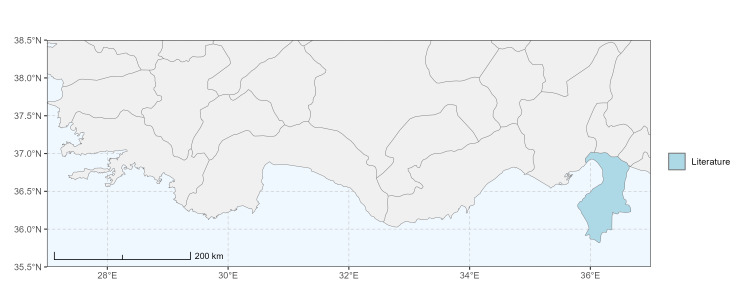
Distribution of *A. isabellina* in the Mediterranean Region of Türkiye.

Hatay (Antakya) (Warncke 1974); Şırnak (Uludere), Hakkari, Gaziantep (Gusenleitner and Schwarz 2000); Hatay (this study) (Fig. 45).

#### Flower records

**Asteraceae**: *Crepis sancta* (L.) Bornm.

### Andrena (Ulandrena) kriechbaumeri

Schmiedeknecht, 1883

8EADCF4F-6DD4-5292-AAB8-AB58CECED205

#### Materials

- **Type status:** Other material. **Occurrence:** recordedBy: Fatih Dikmen; individualCount: 5; sex: male; occurrenceID: 2215AACF-79EF-5228-889B-7B8EC0DECDEC; **Location:** countryCode: TR; stateProvince: Mersin; county: Mut; locality: Silifke road; verbatimElevation: 124 m; verbatimLatitude: 36.569; verbatimLongitude: 33.449; **Identification:** identifiedBy: Thomas J. Wood; **Event:** eventDate: 22-05-2009; **Record Level:** collectionCode: ZMUI
- **Type status:** Other material. **Occurrence:** recordedBy: Fatih Dikmen; individualCount: 2; sex: female; occurrenceID: 0E892810-E3DF-506B-94BC-1FFFA2F8E489; **Location:** countryCode: TR; stateProvince: Muğla; county: Marmaris; municipality: Karacasöğüt; verbatimElevation: 0 m; verbatimLatitude: 36.941; verbatimLongitude: 28.185; **Identification:** identifiedBy: Thomas J. Wood; **Event:** eventDate: 21-06-2009; **Record Level:** collectionCode: ZMUI

#### Distribution

Bulgaria, Croatia, Greece, Iraq, Italy, North Macedonia, Syria and Türkiye (Gusenleitner and Schwarz 2002, Wood et al. 2024).

##### Distribution in Türkiye

**Figure 46.**
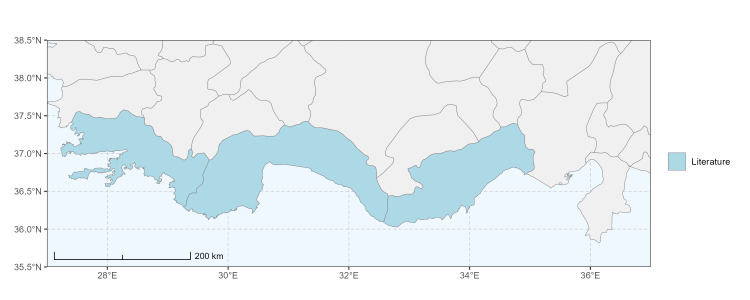
Distribution of *A. kriechbaumeri* in the Mediterranean Region of Türkiye.

Ankara, Amasya (Warncke 1974); Ankara, Antalya, Balıkesir, Kırklareli, Mersin, Muğla (Hazır 2010); Mersin, Muğla (this study) (Fig. 46).

#### Flower records

**Asteraceae**: *Crepis foetida* L. (Lanuza et al. 2025); **Asteraceae**: *Crepis foetida* L., Crepis foetida subsp. rhoeadifolia (M.Bieb.) Čelak.

### Andrena (Holandrena) labialis

(Kirby, 1802)

82D9B6EE-82C3-56A1-902D-77F77493A92F

#### Materials

- **Type status:** Other material. **Occurrence:** recordedBy: Fatih Dikmen; individualCount: 1; sex: female; occurrenceID: F5C1A4E1-F5D0-5425-96C6-6285262077C2; **Location:** countryCode: TR; stateProvince: Adana; county: Tufanbeyli; locality: Damlalık village; verbatimElevation: 1569 m; verbatimLatitude: 38.33; verbatimLongitude: 36.167; **Identification:** identifiedBy: Thomas J. Wood; **Event:** eventDate: 17-07-2009; **Record Level:** collectionCode: ZMUI
- **Type status:** Other material. **Occurrence:** recordedBy: Fatih Dikmen; individualCount: 2; sex: male; occurrenceID: 05FD7DD1-44BA-52C5-9B1D-881E315392F0; **Location:** countryCode: TR; stateProvince: Mersin; county: Mut; locality: Güzlek Plateau; verbatimElevation: 1414 m; verbatimLatitude: 36.654; verbatimLongitude: 33.107; **Identification:** identifiedBy: Thomas J. Wood; **Event:** eventDate: 04-06-2009; **Record Level:** collectionCode: ZMUI

#### Distribution

Albania, Algeria, Austria, Belgium, Bulgaria, Croatia, Denmark, Estonia, Finland, France, Germany, Great Britain, Greece, Hungary, Italy, Latvia, Luxembourg, Morocco, Netherlands, Poland, Portugal, Romania, Russia, Slovakia, Slovenia, Spain, Sweden, Switzerland, Tunisia, Türkiye and Ukraine (Gusenleitner and Schwarz 2002, Osytshnjuk et al. 2005, Wood and Monfared 2022, Ascher and Pickering 2025).

##### Distribution in Türkiye

**Figure 47.**
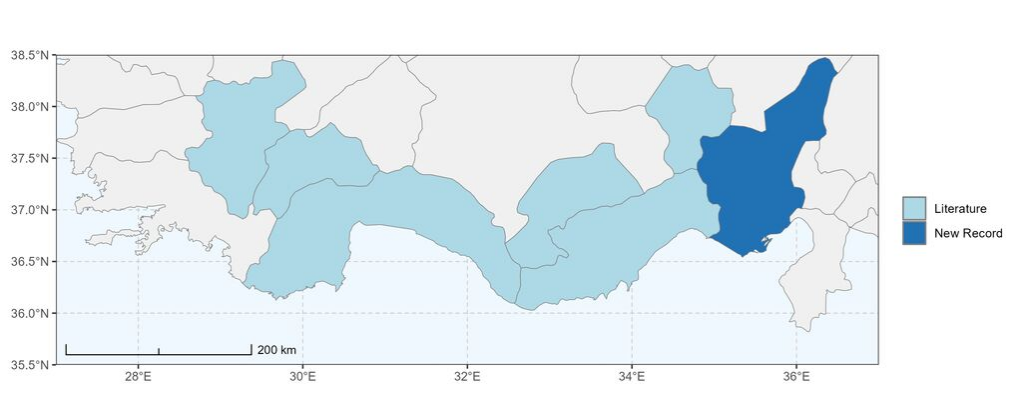
Distribution of *A. labialis* in the Mediterranean Region of Türkiye.

Ankara (Şereflikoçhisar), Antalya (Finike), Aydın (Bozdoğan), Bursa (Uludağ), Erzurum (İspir, Oltu), Gümüşhane (Zigana), Karaman (Madenşehri), Kars, Konya, Niğde (Ulukışla), Tatos Mountains, Tunceli (Warncke 1974); Bayburt, Elazığ, Erzurum (Hınıs, Horasan, İspir, Narman, Oltu, Tortum), Iğdır, Muş (Özbek 1976); Burdur, Çanakkale, Denizli, Erzincan, Karabük, Kastamonu, Kayseri, Konya, Mersin, Sivas (Hazır 2010, Hazır et al. 2014); Aydın (Kuşadası), Kütahya, Mardin, Mersin, Van (Wood 2024); Adana, Mersin (this study) (Fig. 47).

#### Flower records

**Euphorbiaceae**: *Euphorbia* L. **Fabaceae**: *Astragalus* L. **Lamiaceae**: *Mentha longifolia* (L.) L., *Phlomis olivieri* Benth. (Khodarahmi Ghahnavieh and Monfared 2019); **Apiaceae**: *Heracleum sphondylium* L. **Asteraceae**: *Bellis perennis* L., *Hieracium* L., *Leucanthemum vulgare* Lam., *Senecio jacobaea* L., *Taraxacum* agg. F.H.Wigg. **Brassicaceae**: *Raphanus raphanistrum* L., *Sinapis alba* L., *Sinapis* L. **Caryophyllaceae**: *Stellaria graminea* L. **Cistaceae**: *Helianthemum nummularium* (L.) Mill. **Ericaceae**: *Erica* Tourn. ex L., *Vaccinium myrtillus* L. **Fabaceae**: *Cytisus scoparius* (L.) Link, *Genista tinctoria* L., *Lathyrus* L., *Lotus corniculatus* L., *Lotus* L., *Medicago* L., *Onobrychis viciifolia* Scop., *Trifolium hybridum* L., *Trifolium pratense* L., *Trifolium repens* L., *Trifolium* Tourn. ex L., *Ulex europaeus* L., *Ulex* L. **Ranunculaceae**: *Ranunculus acris* L., *Ranunculus* L. **Rosaceae**: *Filipendula ulmaria* (L.) Maxim., *Rosa* L., *Rubus fruticosus* agg. L. (Balfour et al. 2022); **Apiaceae**: *Daucus carota* L., *Thapsia villosa* L., *Torilis arvensis* (Huds.) Link **Asteraceae**: *Carduus crispus* L. **Caprifoliaceae**: *Knautia arvensis* (L.) Coult. **Cistaceae**: *Cistus crispus* L. **Fabaceae**: *Genista hirsuta* Vahl, *Hippocrepis comosa* L., *Lotus corniculatus* L., *Lotus dorycnium* L., *Lotus germanicus* (Gremli) Peruzzi, *Medicago sativa* L., *Melilotus albus* Medik., *Onobrychis viciifolia* Scop., *Ononis spinosa* L., *Trifolium campestre* Schreb., *Trifolium hybridum* L., *Trifolium montanum* L., *Trifolium pratense* L., *Trifolium repens* L. **Lamiaceae**: *Salvia verticillata* L., *Thymus vulgaris* L. **Malvaceae**: *Malva sylvestris* L. **Plumbaginaceae**: *Armeria caespitosa* (Ortega) Boiss. (Lanuza et al. 2025); **Asteraceae**: *Scorzonera cana* (C.A.Mey.) Griseb., *Onopordum boissierianum* Raab-Straube & Greuter **Fabaceae**: *Astragalus* L., Vicia villosa subsp. eriocarpa (Hausskn.) P.W.Ball.

### Andrena (Aciandrena) lamiana

Warncke, 1965

4B0CF909-6D18-5C6B-9430-444758D36FC7

#### Materials

- **Type status:** Other material. **Occurrence:** recordedBy: Fatih Dikmen; individualCount: 1; sex: male; occurrenceID: 27726EE8-9AAA-5F58-815A-38398FA63E07; **Location:** countryCode: TR; stateProvince: Muğla; county: Marmaris; locality: Bördübet road; verbatimElevation: 195 m; verbatimLatitude: 36.857; verbatimLongitude: 28.126; **Identification:** identifiedBy: Thomas J. Wood; **Event:** eventDate: 04-04-2009; **Record Level:** collectionCode: ZMUI

#### Distribution

Cyprus, Greece, Iran, North Macedonia, Syria and Türkiye (Gusenleitner and Schwarz 2002, Wood and Monfared 2022).

##### Distribution in Türkiye

**Figure 48.**
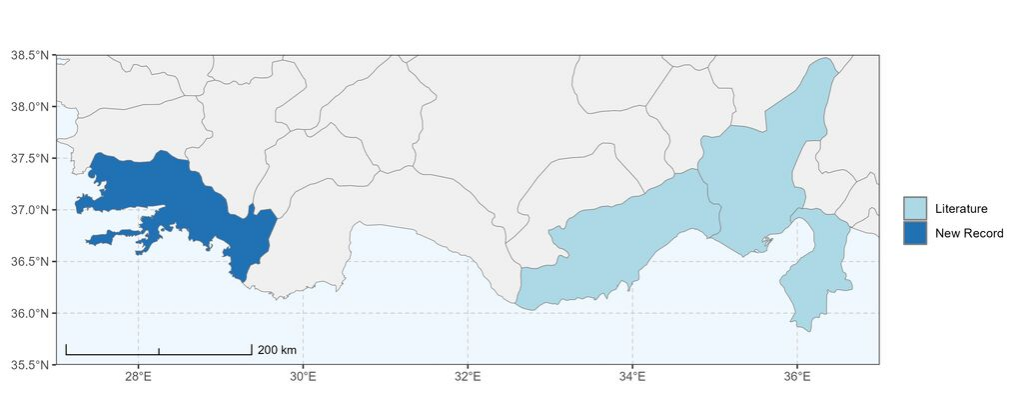
Distribution of *A. lamiana* in the Mediterranean Region of Türkiye.

Adana, Amasya, Balıkesir (Ayvalık), Eskişehir (Sivrihisar), Hatay, Konya (Sarayönü), Malatya, Sakarya (Adapazarı) (Warncke 1966); Ankara (Hazır et al. 2014); Antep, Kütahya, Mardin, Mersin, Van (Wood 2024); Muğla (this study) (Fig. 48).

#### Flower records

**Asteraceae**: *Crepis commutata* (Spreng.) Greuter, *Glebionis coronaria* (L.) Cass. ex Spach, *Senecio leucanthemifolius* Poir. **Brassicaceae**: *Hirschfeldia incana* (L.) Lagr.-Foss., *Sinapis alba* L. (Lanuza et al. 2025); **Brassicaceae**: *Isatis* L.

### Andrena (Poecilandrena) laticeps

Morawitz, 1877

C66A5EC7-41E9-5C04-B693-6E14E2A821D3

#### Materials

- **Type status:** Other material. **Occurrence:** recordedBy: Fatih Dikmen; individualCount: 2; sex: female; occurrenceID: 2FA7DDC0-06E9-5C51-BAF3-EC2F6A47944D; **Location:** countryCode: TR; stateProvince: Niğde; county: Çamardı; verbatimElevation: 1350 m; verbatimLatitude: 38.066; verbatimLongitude: 34.794; **Identification:** identifiedBy: Thomas J. Wood; **Event:** eventDate: 23-05-2008; **Record Level:** collectionCode: ZMUI

#### Distribution

**Distribution.** Armenia, Georgia, Iran, Iraq and Türkiye (Wood and Monfared 2022, Wood et al. 2024, Ascher and Pickering 2025).

##### Distribution in Türkiye

**Figure 49.**
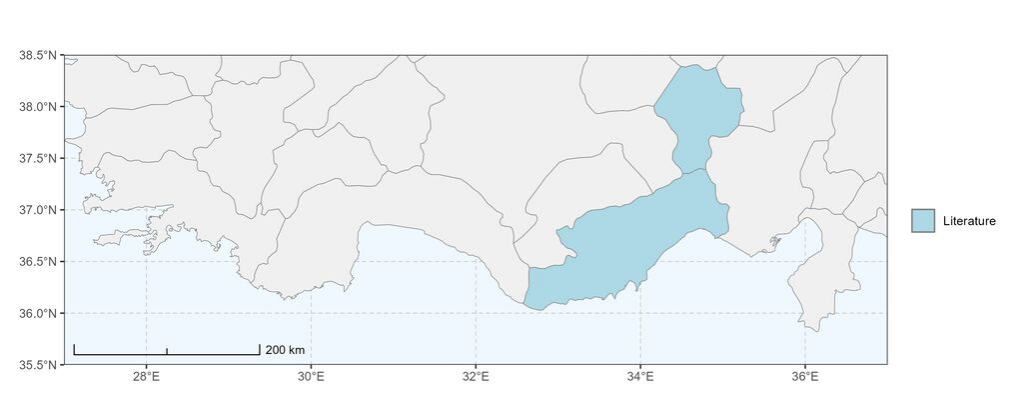
Distribution of *A. laticeps* in the Mediterranean Region of Türkiye.

Kayseri, Konya (Warncke 1966; Niğde (Ulukışla), Mersin (Sertavul), Sakarya (Adapazarı) (Warncke 1974); Erzincan (Refahiye) (Özbek 1976); Ankara, Karaman, Kütahya, Mersin, Niğde (Hazır 2010, Hazır et al. 2014); Hakkari, Van (Wood and Monfared 2022); Niğde (this study) (Fig. 49).

#### Flower records

**Plantaginaceae**: *Veronica* L. (Warncke 1974)

### Andrena (Simandrena) lepida

Schenck, 1861

728516D4-AE41-561F-9053-505874ECF361

#### Materials

- **Type status:** Other material. **Occurrence:** recordedBy: Fatih Dikmen; individualCount: 1; sex: female; occurrenceID: E69AEE1E-06E6-59F7-916D-2AC5A3CCFA71; **Location:** countryCode: TR; stateProvince: Hatay; county: Kırıkhan; locality: Reyhanlı; verbatimElevation: 85 m; verbatimLatitude: 36.508; verbatimLongitude: 36.418; **Identification:** identifiedBy: Thomas J. Wood; **Event:** eventDate: 21-03-2009; **Record Level:** collectionCode: ZMUI

#### Distribution

Algeria, Austria, Bulgaria, Corsica, Cyprus, France, Germany, Great Britain, Greece, Hungary, Italy, Lithuania, Morocco, Morovya, Poland, Portugal, Romania, Russia, Slovakia, Spain, Switzerland, Türkiye and Ukraine (Gusenleitner and Schwarz 2002, Ascher and Pickering 2025).

##### Distribution in Türkiye

**Figure 50.**
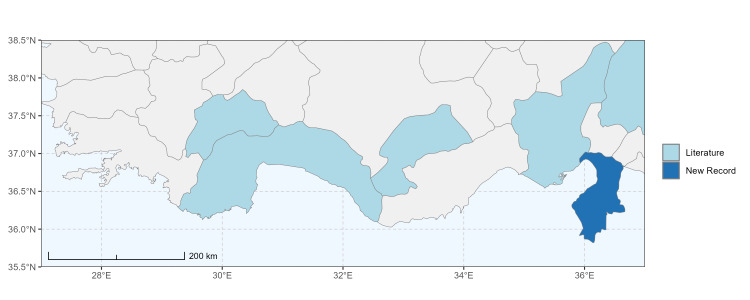
Distribution of *A. lepida* in the Mediterranean Region of Türkiye.

Ankara, Balıkesir (Ayvalık), Eskişehir, Konya (Warncke 1966); Erzurum (Özbek 1976); Adana, Ankara, Antalya, Aydın, Burdur, Çorum, Denizli, Karaman, Kastamonu, Kırıkkale, Kırşehir, Konya, Maraş, Yozgat (Hazır 2010); Hatay (this study) (Fig. 50).

#### Flower records

**Cistaceae**: *Cistus albidus* L., *Cistus monspeliensis* L., *Cistus salviifolius* L. **Fabaceae**: *Vicia lutea* L. (Lanuza et al. 2025); **Brassicaceae**: *Ochthodium aegyptiacum* (L.) DC.

### Andrena (Melandrena) limata

Smith, 1853

82C9ACCB-816F-5FD2-990D-D51299E68DC1

#### Materials

- **Type status:** Other material. **Occurrence:** recordedBy: Fatih Dikmen; individualCount: 1; sex: female; occurrenceID: 20CFE819-39F6-5302-88CC-C98DE7508CF8; **Location:** countryCode: TR; stateProvince: Antalya; locality: Side hotels; verbatimElevation: 13 m; verbatimLatitude: 36.79; verbatimLongitude: 31.399; **Identification:** identifiedBy: Thomas J. Wood; **Event:** eventDate: 13-04-2009; **Record Level:** collectionCode: ZMUI

#### Distribution

Algeria, Austria, Belgium, Bulgaria, Cyprus, Finland, France, Germany, Greece, Italy, Iraq, Iran, Latvia, Lithuania, Luxembourg, Morocco, Poland, Portugal, Romania, Russia, Slovenia, Spain, Switzerland, Türkiye and Ukraine (Gusenleitner and Schwarz 2002, Osytshnjuk et al. 2008, Wood and Monfared 2022, Wood et al. 2024, Ascher and Pickering 2025).

##### Distribution in Türkiye

**Figure 51.**
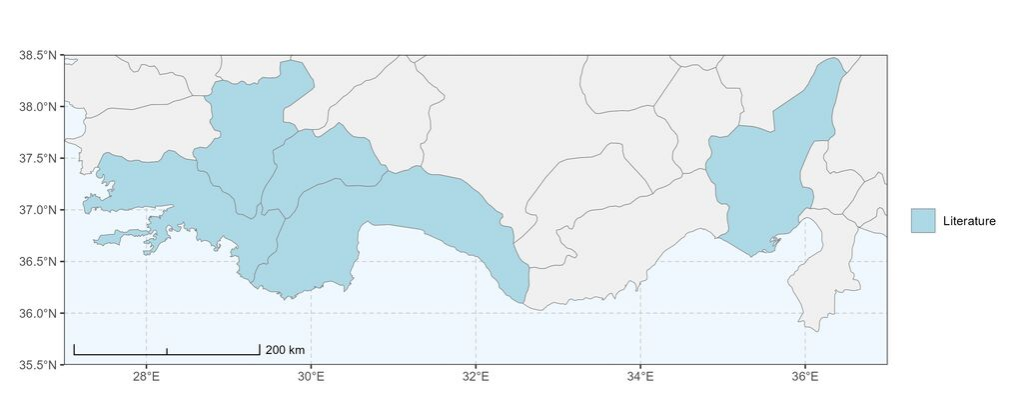
Distribution of *A. limata* in the Mediterranean Region of Türkiye.

Adana, Balıkesir (Ayvalık), Burdur, Nevşehir (Ürgüp), Sakarya (Adapazarı), Toros Mountains (Warncke 1966); Aydın (Germencik), İzmir (Ensetepe) (Warncke 1969); Erzurum (Tortum, Oltu) (Özbek 1976); Ankara, Antalya, Aydın, Bolu, Denizli, Erzurum, Manisa, Muğla, Uşak (Hazır 2010, Hazır et al. 2014); Antalya (this study) (Fig. 51).

#### Flower records

**Asteraceae**: *Chamaemelum fuscatum* (Brot.) Vasc. **Cistaceae**: *Cistus parviflorus* Lam. **Hydrangeaceae**: *Deutzia scabra* Thunb., *Philadelphus coronarius* L., *Philadelphus incanus* Koehne **Lamiaceae**: *Origanum onites* L. **Plantaginaceae**: *Plantago lanceolata* L., *Thymbra capitata* (L.) Cav. **Rosaceae**: *Crataegus azarolus* L., *Crataegus monogyna* Jacq., *Photinia* Lindl., *Spiraea* L., *Spiraea blumei* G.Don, *Spiraea japonica* L.f. **Solanaceae**: *Eriolarynx australis* (Griseb.) J.M.H.Shaw (Lanuza et al. 2025); **Brassicaceae**: *Sinapis arvensis* L.

### Andrena (Melandrena) magna

Warncke, 1965

9495761C-5CBC-5509-9BBA-0B6F6F1821FF

#### Materials

- **Type status:** Other material. **Occurrence:** recordedBy: Fatih Dikmen; individualCount: 2; sex: female; occurrenceID: B6A4A593-6443-551D-8E96-07F51410A79A; **Location:** countryCode: TR; stateProvince: Denizli; locality: Ankara road, 3 km Sanayi-Kocabaş; verbatimElevation: 439 m; verbatimLatitude: 37.823; verbatimLongitude: 29.293; **Identification:** identifiedBy: Thomas J. Wood; **Event:** eventDate: 12-06-2009; **Record Level:** collectionCode: ZMUI

#### Distribution

Greece, Romania, Türkiye and Ukraine (Gusenleitner and Schwarz 2002).

##### Distribution in Türkiye

**Figure 52.**
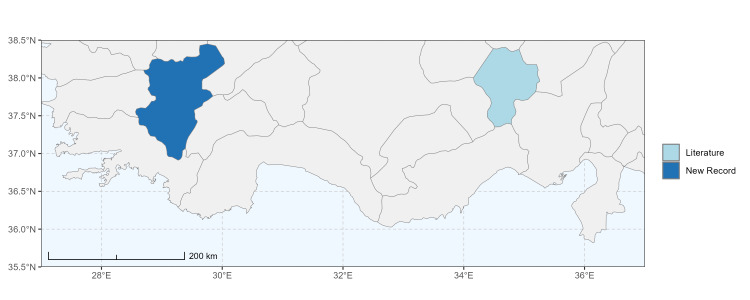
Distribution of *A. magna* in the Mediterranean Region of Türkiye.

Amasya (Warncke 1966); Ankara (Gölbaşı), Kayseri (Yeşilhisar), Konya (Akşehir, Kulu), Niğde (Ulukışla) (Warncke 1974); Denizli (this study) (Fig. 52).

#### Flower records

**Asteraceae**: *Onopordum acanthium* L.

### Andrena (Micrandrena) minutula

(Kirby, 1802)

9768EC44-55B0-5A0C-9374-10FB99D16011

#### Materials

- **Type status:** Other material. **Occurrence:** recordedBy: Fatih Dikmen; individualCount: 1; sex: female; occurrenceID: 04A5AD37-1A5E-5437-9912-71AB8AB9EF5D; **Location:** countryCode: TR; stateProvince: Osmaniye; locality: Karatepe National Park; verbatimElevation: 325 m; verbatimLatitude: 37.26; verbatimLongitude: 36.219; **Identification:** identifiedBy: Thomas J. Wood; **Event:** eventDate: 20-03-2009; **Record Level:** collectionCode: ZMUI
- **Type status:** Other material. **Occurrence:** recordedBy: Fatih Dikmen; individualCount: 1; sex: female; occurrenceID: D01C194A-D3B7-5275-8346-DFFB14D8E880; **Location:** countryCode: TR; stateProvince: Muğla; locality: Datça road, Turunç; verbatimElevation: 347 m; verbatimLatitude: 36.774; verbatimLongitude: 28.22; **Identification:** identifiedBy: Thomas J. Wood; **Event:** eventDate: 03-04-2009; **Record Level:** collectionCode: ZMUI
- **Type status:** Other material. **Occurrence:** recordedBy: Fatih Dikmen; individualCount: 1; sex: female; occurrenceID: C41EFA18-7851-537B-AEDE-DC813CDDC923; **Location:** countryCode: TR; stateProvince: Muğla; county: Marmaris; locality: Bördübet road; verbatimElevation: 195 m; verbatimLatitude: 36.857; verbatimLongitude: 28.126; **Identification:** identifiedBy: Thomas J. Wood; **Event:** eventDate: 04-04-2009; **Record Level:** collectionCode: ZMUI
- **Type status:** Other material. **Occurrence:** recordedBy: Fatih Dikmen; individualCount: 1; sex: female; occurrenceID: CE75B73B-DED1-5B21-BE5E-5366BB226A20; **Location:** countryCode: TR; stateProvince: Antalya; county: Korkuteli; municipality: Çomaklı; verbatimElevation: 838 m; verbatimLatitude: 37.267; verbatimLongitude: 30.313; **Identification:** identifiedBy: Thomas J. Wood; **Event:** eventDate: 29-05-2009; **Record Level:** collectionCode: ZMUI

#### Distribution

Algeria, Armenia, Australia, Austria, Azerbaijan, Bosnia and Herzegovina, Bulgaria, China, Croatia, Cyprus, Czechia, Denmark, France, Georgia, Germany, Great Britain, Iran, Japan, Lebanon, Libya, Morocco, Netherlands, New Caledonia, Norway, Poland, Portugal, Russia, Slovenia, Spain, Sweden, Switzerland, Türkiye and Ukraine (Gusenleitner and Schwarz 2002, Wood et al. 2020, Pisanty et al. 2022, Wood and Monfared 2022, Ascher and Pickering 2025).

##### Distribution in Türkiye

**Figure 53.**
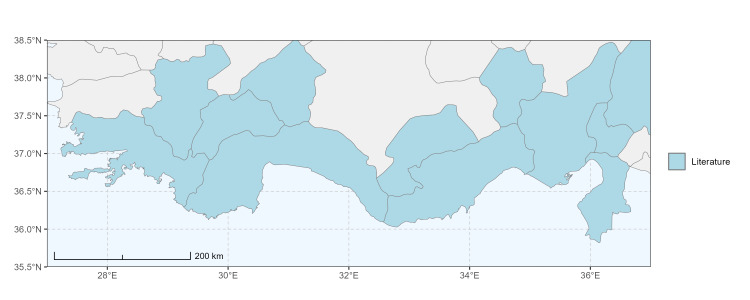
Distribution of *A. minutula* in the Mediterranean Region of Türkiye.

In all parts of the country (Warncke 1974); Antalya, Muğla, Osmaniye (this study) (Fig. 53).

#### Flower records

**Apiaceae**: *Anthriscus sylvestris* (L.) Hoffm., *Conopodium majus* (Gouan) Loret, *Daucus carota* L., *Heracleum sphondylium* L., *Pastinaca sativa* L., *Torilis japonica* (Houtt.) DC. **Asteraceae**: *Achillea millefolium* L., *Centaurea nigra* L., *Centaurea scabiosa* L., *Cirsium arvense* (L.) Scop., *Cirsium* Mill., *Crepis capillaris* (L.) Wallr., *Hieracium* agg. L., *Leucanthemum vulgare* Lam., *Matricaria chamomilla* L., *Pilosella officinarum* F.W.Schultz & Sch.Bip., *Sonchus arvensis* L., *Taraxacum* agg. F.H.Wigg., *Taraxacum officinale* agg. F.H.Wigg., *Tripleurospermum inodorum* (L.) Sch.Bip. **Brassicaceae**: *Brassica napus* L., *Sinapis arvensis* L. **Convolvulaceae**: *Convolvulus arvensis* L. **Fabaceae**: *Trifolium* Tourn. ex L. **Geraniaceae**: *Geranium molle* L. **Hypericaceae**: *Hypericum perforatum* L. **Lamiaceae**: *Mentha aquatica* L. **Ranunculaceae**: *Ranunculus bulbosus* L., *Ranunculus repens* L., **Rosaceae**: *Rubus fruticosus* agg. L. (Balfour et al. 2022); **Apiaceae**: *Anthriscus sylvestris* (L.) Hoffm., *Apium graveolens* L., *Astrantia major* L., *Chaerophyllum bulbosum* L., *Chaerophyllum temulum* L., *Daucus carota* L., *Falcaria vulgaris* Bernh., *Heracleum sphondylium* L., *Oenanthe pimpinelloides* L., *Pimpinella major* (L.) Huds., *Pimpinella saxifraga* L., *Pimpinella peregrina* L., *Torilis arvensis* (Huds.) Link, *Torilis japonica* (Houtt.) DC. **Asteraceae**: *Achillea millefolium* L., *Bellis annua* L., *Bellis perennis* L., *Centaurea jacea* L., *Centaurea nigra* L., *Cichorium intybus* L., *Cirsium arvense* (L.) Scop., *Crepis* L., *Crepis capillaris* (L.) Wallr., *Leontodon hispidus* L., *Matricaria chamomilla* L., *Picris hieracioides* L., *Scorzonera humilis* L., *Senecio* L., *Senecio inaequidens* DC., *Senecio leucanthemifolius* Poir., *Senecio vernalis* Waldst. & Kit., *Tanacetum vulgare* L., *Taraxacum campylodes* G.E.Haglund, *Taraxacum officinale* F.H.Wigg., *Pilosella officinarum* F.W.Schultz & Sch.Bip. **Brassicaceae**: *Barbarea vulgaris* W.T.Aiton, *Biscutella laevigata* L., *Brassica arvensis* L., *Brassica napus* L., *Bunias orientalis* L., *Cakile maritima* Scop., *Erysimum crepidifolium* Rchb., *Hirschfeldia incana* (L.) Lagr.-Foss., *Lepidium draba* L., *Rorippa sylvestris* (L.) Besser, *Sisymbrium loeselii* L. **Caryophyllaceae**: *Dianthus hyssopifolius* L., *Stellaria media* (L.) Vill. **Cistaceae**: *Helianthemum nummularium* (L.) Mill. **Convolvulaceae**: *Calystegia sepium* (L.) R.Br., *Convolvulus arvensis* L. **Crassulaceae**: *Sedum acre* L., *Sedum album* L., *Sedum sexangulare* L. **Ericaceae**: *Daboecia cantabrica* (Huds.) K.Koch **Fabaceae**: *Astragalus monspessulanus* L., *Hippocrepis comosa* L., *Lotus corniculatus* L., *Lotus dorycnium* L., *Medicago lupulina* L., *Medicago sativa* L., *Melilotus albus* Medik., *Melilotus officinalis* (L.) Lam., *Onobrychis viciifolia* Scop., *Ononis spinosa* L., *Trifolium campestre* Schreb., *Trifolium pratense* L., *Trifolium repens* L., *Trifolium montanum* L. **Geraniaceae**: *Geranium pratense* L., *Geranium pyrenaicum* Burm.f. **Hypericaceae**: *Hypericum perforatum* L. **Lamiaceae**: *Teucrium capitatum* L., *Thymus praecox* Opiz, *Thymus pulegioides* L. **Linaceae**: *Linum catharticum* L., *Linum suffruticosum* L. **Orchidaceae**: *Himantoglossum hircinum* (L.) Spreng. **Oxalidaceae**: *Oxalis corniculata* L. **Plantaginaceae**: *Plantago lanceolata* L., *Veronica chamaedrys* L., *Veronica persica* Poir. **Ranunculaceae**: *Ranunculus* L., *Ranunculus bulbosus* L. **Rosaceae**: *Fragaria viridis* Weston, *Geum urbanum* L., *Potentilla erecta* (L.) Raeusch., *Potentilla reptans* L., *Potentilla verna* L., *Prunus mahaleb* L., *Rubus* L., *Rubus caesius* L. (Lanuza et al. 2025); **Asteraceae**: *Anthemis chia* L., *Senecio vernalis* Waldst. & Kit. **Brassicaceae**: *Isatis* L. **Convolvulaceae**: *Convolvulus* L.

### Andrena monacha

Warncke, 1965

7CF0B44D-C61D-51BA-AEA3-CA7E76107B5C

#### Materials

- **Type status:** Other material. **Occurrence:** recordedBy: Fatih Dikmen; individualCount: 1; sex: female; occurrenceID: 50A96BCC-BC5A-5DE0-A37A-05BE2ED3AA38; **Location:** countryCode: TR; stateProvince: Mersin; county: Mut; locality: Güzlek Plateau; verbatimElevation: 1414 m; verbatimLatitude: 36.654; verbatimLongitude: 33.107; **Identification:** identifiedBy: Thomas J. Wood; **Event:** eventDate: 04-06-2009; **Record Level:** collectionCode: ZMUI

#### Distribution

Cyprus, Greece, Iran, Iraq, Lebanon, Syria and Türkiye (Gusenleitner and Schwarz 2002, Wood et al. 2020, Wood and Monfared 2022, Wood et al. 2024).

##### Distribution in Türkiye

**Figure 54.**
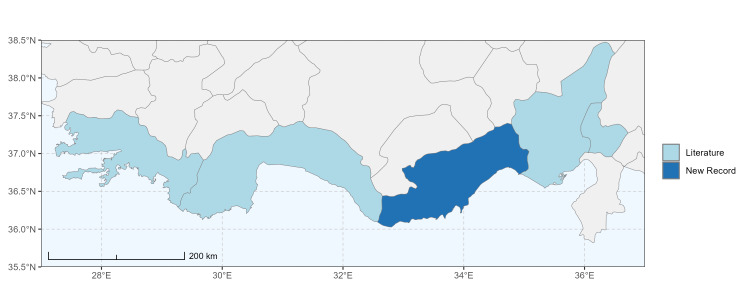
Distribution of *A. monacha* in the Mediterranean Region of Türkiye.

Sakarya (Adapazarı), Balıkesir (Ayvalık), Toroslar (Warncke 1966, Blank and Kraus 1994); Adana (Karataş), Antalya (Aspendos, Finike, Side), Çanakkale (Bigadiç), Muğla, Osmaniye (Warncke 1974); Antalya, Aydın (Hazır 2010, Hazır et al. 2014); Mersin (this study) (Fig. 54).

#### Flower records

**Asteraceae**: *Crepis alpina* L.

### Andrena (Hamandrena) nasuta

Giraud, 1863

DC45A81E-D59C-5335-8667-3A32BC16ABB1

#### Materials

- **Type status:** Other material. **Occurrence:** recordedBy: Fatih Dikmen; individualCount: 1; sex: female; occurrenceID: 7FD0DCF4-D4F0-5D44-8FD1-1DF8203F4F1F; **Location:** countryCode: TR; stateProvince: Mersin; county: Mut; locality: Güzlek Plateau; verbatimElevation: 1414 m; verbatimLatitude: 36.654; verbatimLongitude: 33.107; **Identification:** identifiedBy: Thomas J. Wood; **Event:** eventDate: 04-06-2009; **Record Level:** collectionCode: ZMUI

#### Distribution

Armenia, Austria, Bulgaria, Czech Republic, Germany, Greece, Hungary, Iran, Lithuania, Poland, Romania, Slovakia, Switzerland and Türkiye (Gusenleitner and Schwarz 2002).

##### Distribution in Türkiye

**Figure 55.**
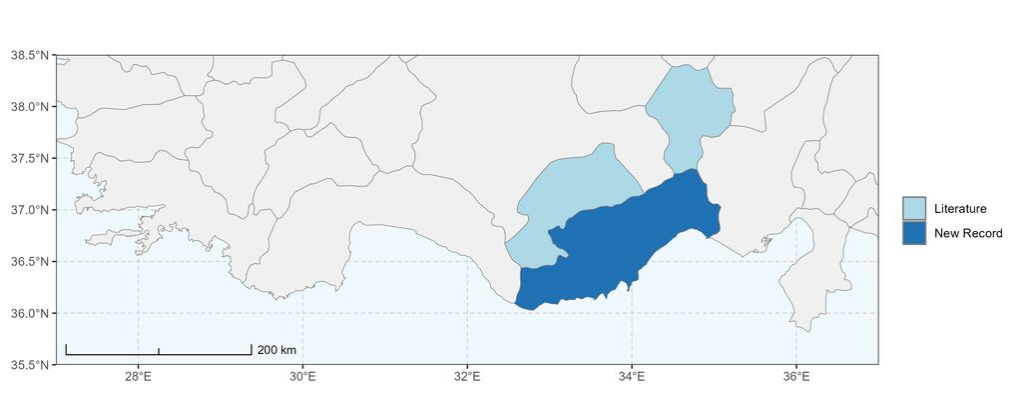
Distribution of *A. nasuta* in the Mediterranean Region of Türkiye.

Ankara (Warncke 1966); Erzurum, Karaman, Konya, Niğde (Warncke 1974); Ankara, Aydın, Karaman, Kayseri, Kütahya (Hazır 2010, Hazır et al. 2014); Mersin (this study) (Fig. 55).

#### Flower records

**Boraginaceae**: Anchusa leptophylla subsp. leptophylla Roem. & Schult.

### Andrena (Ulandrena) neocypriaca

Mavromoustakis, 1956

3516C497-E63F-50C9-ADB2-CCEC8C13C030

#### Materials

- **Type status:** Other material. **Occurrence:** recordedBy: Fatih Dikmen; individualCount: 6; sex: female; occurrenceID: 11A2663E-532D-5E07-B9F3-286EA1C14EFF; **Location:** countryCode: TR; stateProvince: Mersin; county: Mut; locality: Silifke road, Sarıkavak turn; verbatimElevation: 223 m; verbatimLatitude: 36.503; verbatimLongitude: 33.585; **Identification:** identifiedBy: Thomas J. Wood; **Event:** eventDate: 22-05-2009; **Record Level:** collectionCode: ZMUI

#### Distribution

Cyprus, Greece and Türkiye (Gusenleitner and Schwarz 2002, Wood 2021b).

##### Distribution in Türkiye

**Figure 56.**
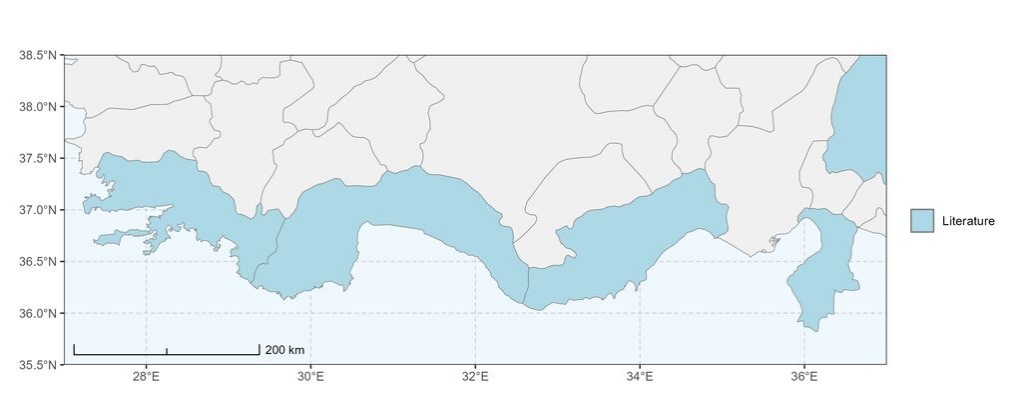
Distribution of *A. neocypriaca* in the Mediterranean Region of Türkiye.

Hatay, Sakarya (Adapazarı) (Warncke 1966); Antalya (Anamur, Side), İzmir, Muğla (Fethiye, Marmaris), Urfa (Birecik) (Warncke 1974); Aydın, Antep, Maraş, Mersin (Hazır 2010); Mersin (this study) (Fig. 56).

#### Flower records

**Asteraceae**: *Crepis foetida* L.

### Andrena (Melandrena) nigroaenea

(Kirby, 1802)

F787CAE4-6903-5F44-A844-927F3554B796

#### Materials

- **Type status:** Other material. **Occurrence:** recordedBy: Fatih Dikmen; individualCount: 2; sex: female; occurrenceID: A14B1A92-BF7E-5E9C-B7A8-A88282C925AD; **Location:** countryCode: TR; stateProvince: Antalya; locality: Side hotels; verbatimElevation: 13 m; verbatimLatitude: 36.79; verbatimLongitude: 31.399; **Identification:** identifiedBy: Thomas J. Wood; **Event:** eventDate: 13-04-2009; **Record Level:** collectionCode: ZMUI
- **Type status:** Other material. **Occurrence:** recordedBy: Fatih Dikmen; individualCount: 1; sex: female; occurrenceID: 11824214-303F-5888-A310-059419775634; **Location:** countryCode: TR; stateProvince: Antalya; locality: Alanya entrance; verbatimElevation: 11 m; verbatimLatitude: 36.656; verbatimLongitude: 31.701; **Identification:** identifiedBy: Thomas J. Wood; **Event:** eventDate: 14-04-2009; **Record Level:** collectionCode: ZMUI

#### Distribution

Afghanistan, Algeria, Armenia, Austria, Belgium, Bosnia and Herzegovina, Bulgaria, Cyprus, Croatia, Czechia, Democratic Republic of the Congo, Denmark, Egypt, Estonia, Finland, France, Georgia, Germany, Great Britain, Greece, Hungary, Iran, Israel and the West Bank, Italy, Latvia, Lebanon, Libya, Morocco, Netherlands, New Caledonia, Norway, Poland, Portugal, Romania, Russia, Slovakia, Spain, Syria, Ukraine, Tunisia and Türkiye (Gusenleitner and Schwarz 2002, Ascher and Pickering 2025)

##### Distribution in Türkiye

**Figure 57.**
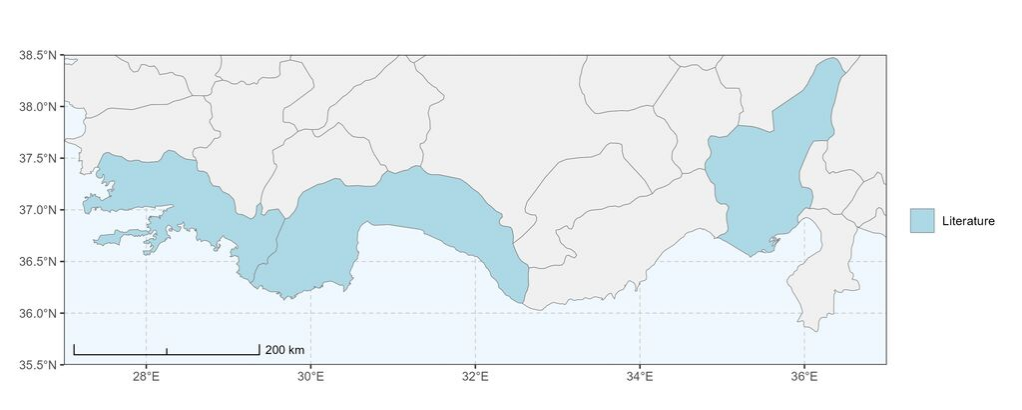
Distribution of *A. nigroaenea* in the Mediterranean Region of Türkiye.

Adana, Balıkesir (Ayvalık), Eskişehir (Sivrihisar), Konya, Malatya, Manisa, Nevşehir (Ürgüp), Toroslar (Warncke 1966); Ankara, Antalya, Aydın, Kastamonu, Kütahya, Muğla (Hazır et al. 2014); Antalya (this study) (Fig. 57).

#### Flower records

**Apiaceae**: *Anthriscus* Pers., *Anthriscus sylvestris* (L.) Hoffm., *Chaerophyllum temulum* L., *Heracleum sphondylium* L., *Oenanthe crocata* L. **Aquifoliaceae**: *Ilex aquifolium* L. **Asteraceae**: *Achillea millefolium* L., *Anthemis* L., *Bellis perennis* L., *Crepis* L., *Leucanthemum vulgare* Lam., *Taraxacum* agg. F.H.Wigg., *Taraxacum officinale* agg. F.H.Wigg., *Taraxacum* F.H.Wigg., *Tripleurospermum inodorum* (L.) Sch.Bip. **Brassicaceae**: *Brassica napus* L., *Cakile maritima* Scop., *Coincya wrightii* (O.E.Schulz) Stace, *Sisymbrium officinale* (L.) Scop. **Caryophyllaceae**: *Silene dioica* (L.) Clairv. **Cistaceae**: *Helianthemum nummularium* (L.) Mill. **Cornaceae**: *Cornus sanguinea* L. **Fabaceae**: *Medicago lupulina* L., *Trifolium hybridum* L., *Trifolium repens* L., *Ulex europaeus* L., *Vicia sativa* L. **Geraniaceae**: *Geranium* Tourn. ex L. **Lamiaceae**: *Stachys sylvatica* L. **Orchidaceae**: *Orchis mascula* (L.) L. **Plantaginaceae**: *Plantago lanceolata* L. **Ranunculaceae**: *Helleborus* Tourn. ex L., *Ranunculus acris* L., *Ranunculus bulbosus* L., *Ranunculus repens* L., *Ranunculus* L. **Resedaceae**: *Reseda lutea* L. **Rhamnaceae**: *Rhamnus cathartica* L. **Rosaceae**: *Crataegus monogyna* Jacq., *Crataegus* L., *Fragaria* L., *Rubus fruticosus* agg. L., *Rubus* L. **Rubiaceae**: *Galium aparine* L. **Salicaceae**: *Salix* L. **Sapindaceae**: *Acer campestre* L. **Solanaceae**: *Solanum dulcamara* L. **Viburnaceae**: *Sambucus nigra* L. (Balfour et al. 2022); **Aizoaceae**: *Carpobrotus acinaciformis* (L.) L.Bolus **Apiaceae**: *Aegopodium podagraria* L., *Daucus carota* L., *Elaeoselinum* W.D.J.Koch ex DC., *Thapsia foetida* L., *Thapsia villosa* L. **Asparagaceae**: *Ornithogalum narbonense* L. **Asteraceae**: *Achillea millefolium* L., *Chamaemelum fuscatum* (Brot.) Vasc., *Crepis foetida* L., *Glebionis coronaria* (L.) Cass. ex Spach, *Leucanthemum vulgare* Lam., *Pilosella officinarum* F.W.Schultz & Sch.Bip., *Taraxacum* F.H.Wigg., *Taraxacum officinale* F.H.Wigg. **Boraginaceae**: *Echium angustifolium* Mill. **Brassicaceae**: *Arabis hirsuta* (L.) Scop., *Aurinia saxatilis* (L.) Desv., *Biscutella laevigata* L., *Brassica juncea* (L.) Czern., *Brassica napus* L., *Brassica repanda* (Willd.) DC., *Bunias orientalis* L., *Erysimum odoratum* Ehrh. **Buxaceae**: *Buxus sempervirens* L. **Caryophyllaceae**: *Viscaria vulgaris* Bernh. **Cistaceae**: *Cistus albidus* L., *Cistus calycinus* L., *Cistus crispus* L., *Cistus ladanifer* L., *Cistus monspeliensis* L., *Helianthemum apenninum* (L.) Mill., *Helianthemum nummularium* (L.) Mill., *Helianthemum oelandicum* (L.) Dum.Cours. **Cornaceae**: *Cornus sanguinea* L. **Cupressaceae**: *Juniperus communis* L. **Fabaceae**: *Anthyllis vulneraria* L., *Hippocrepis comosa* L., *Lotus dorycnium* L., *Lotus fulgurans* (Porta) D.D.Sokoloff, *Onobrychis viciifolia* Scop., *Trifolium pratense* L., *Trifolium repens* L., *Vicia sativa* L. **Hydrangeaceae**: *Philadelphus coronarius* L., *Philadelphus incanus* Koehne **Lamiaceae**: *Lavandula stoechas* L., *Salvia rosmarinus* Spenn., *Teucrium capitatum* L., *Teucrium fruticans* L., *Thymus vulgaris* L. **Papaveraceae**: *Eschscholzia californica* Cham., *Glaucium flavum* Crantz **Plantaginaceae**: *Veronica arvensis* L. **Plumbaginaceae**: *Armeria maritima* (Mill.) Willd., *Plumbago auriculata* Lam. **Primulaceae**: *Primula veris* L. **Ranunculaceae**: *Helleborus foetidus* L., *Ranunculus* L., *Ranunculus acris* L., *Ranunculus auricomus* L., *Ranunculus bulbosus* L. **Resedaceae**: *Reseda lutea* L., *Reseda luteola* L., *Reseda phyteuma* L. **Rosaceae**: *Crataegus monogyna* Jacq., *Potentilla verna* L., *Prunus spinosa* L., *Pyracantha* M.Roem., *Rubus idaeus* L., *Spiraea* L., *Spiraea blumei* G.Don, *Spiraea japonica* L.f. **Rubiaceae**: *Galium pusillum* L. **Rutaceae**: *Ruta angustifolia* Pers., *Ruta montana* (L.) L. **Salicaceae**: *Salix aurita* L., *Salix repens* L. **Saxifragaceae**: *Saxifraga granulata* L. (Lanuza et al. 2025); **Brassicaceae**: *Sinapis arvensis* L.

### Andrena (Melandrena) nitidemula

Scheuchl & Hazır, 2012

943718F3-E4C6-598A-946F-7579D9399332

#### Materials

- **Type status:** Other material. **Occurrence:** recordedBy: Fatih Dikmen; individualCount: 1; sex: female; occurrenceID: 94E50711-6E95-50E3-AE89-660D211DF8D7; **Location:** countryCode: TR; stateProvince: Mersin; county: Mut; locality: Güzlek Plateau; verbatimElevation: 1414 m; verbatimLatitude: 36.654; verbatimLongitude: 33.107; **Identification:** identifiedBy: Thomas J. Wood; **Event:** eventDate: 04-06-2009; **Record Level:** collectionCode: ZMUI
- **Type status:** Other material. **Occurrence:** recordedBy: Fatih Dikmen; individualCount: 1; sex: female; occurrenceID: A0D53D07-D7E2-5C79-96B3-A37133A9CDAE; **Location:** countryCode: TR; stateProvince: Niğde; locality: Çamardı road; verbatimElevation: 1570 m; verbatimLatitude: 37.886; verbatimLongitude: 35.111; **Identification:** identifiedBy: Thomas J. Wood; **Event:** eventDate: 23-05-2008; **Record Level:** collectionCode: ZMUI

#### Distribution

Armenia, Georgia, Greece, Iran, Iraq, Syria and Türkiye (Scheuchl and Hazır 2012, Rasmont et al. 2017, Wood and Monfared 2022, Wood et al. 2024).

##### Distribution in Türkiye

**Figure 58.**
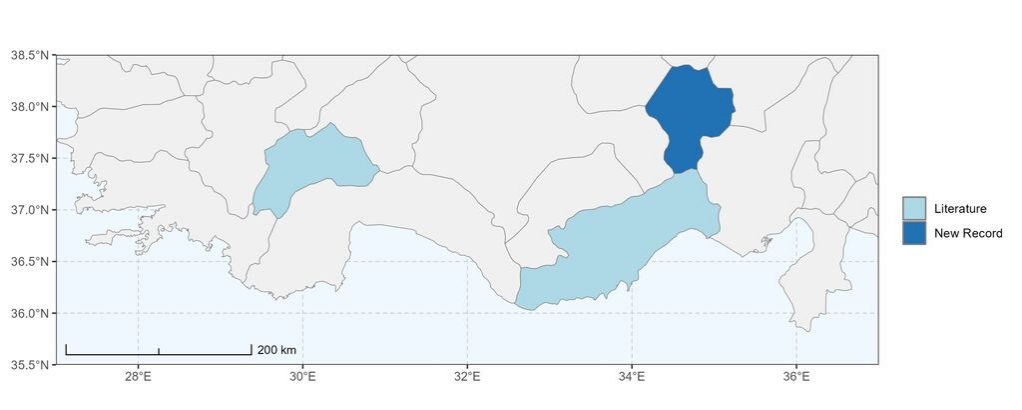
Distribution of *A. nitidemula* in the Mediterranean Region of Türkiye.

Kars (Karakurt) (Scheuchl and Hazır 2012); Ankara, Burdur, Konya, Mersin, Sivas (Hazır et al. 2014); Ankara, Burdur, Hakkari, Konya, Mersin, Sivas (Ascher and Pickering 2025); Mersin, Niğde (this study) (Fig. 58).

#### Flower records

**Brassicaceae**: *Brassica* L.

### Andrena (Nobandrena) nobilis

Morawitz, 1873

ED851035-9C25-5C22-A0CB-5C1296DF6298

#### Materials

- **Type status:** Other material. **Occurrence:** recordedBy: Fatih Dikmen; individualCount: 1; sex: female; occurrenceID: B3F88FC4-87D9-54D6-9340-6B1584E78C41; **Location:** countryCode: TR; stateProvince: Antalya; locality: Korkuteli-Finike road; verbatimElevation: 1346 m; verbatimLatitude: 36.949; verbatimLongitude: 30.131; **Identification:** identifiedBy: Thomas J. Wood; **Event:** eventDate: 10-06-2009; **Record Level:** collectionCode: ZMUI
- **Type status:** Other material. **Occurrence:** recordedBy: Fatih Dikmen; individualCount: 1; sex: female; occurrenceID: EF6CC033-0D40-5D31-8FF7-19247C00EA53; **Location:** countryCode: TR; stateProvince: Mersin; county: Mut; locality: Güzlek Plateau; verbatimElevation: 1414 m; verbatimLatitude: 36.654; verbatimLongitude: 33.107; **Identification:** identifiedBy: Thomas J. Wood; **Event:** eventDate: 04-06-2009; **Record Level:** collectionCode: ZMUI

#### Distribution

Armenia, Austria, Azerbaijan, Bulgaria, Croatia, Czechia, Georgia, Greece, Hungary, Iran, Italy, Poland, Romania, Russia, Slovakia, Turkmenistan, Türkiye and Ukraine (Gusenleitner and Schwarz 2002, Proshchalykin et al. 2017, Wood and Monfared 2022, Ascher and Pickering 2025).

##### Distribution in Türkiye

**Figure 59.**
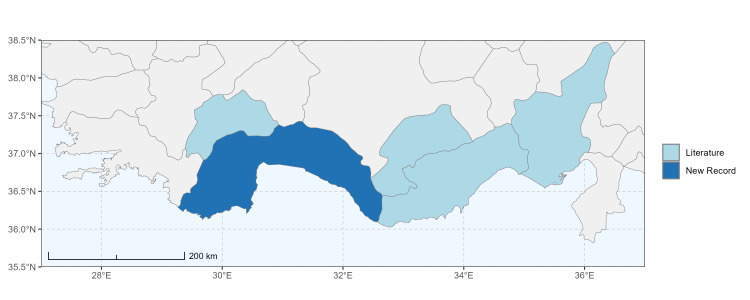
Distribution of *A. nobilis* in the Mediterranean Region of Türkiye.

Aksaray, Ankara, Kayseri, Kırıkkale, Konya (Beyşehir), Toros Mountains (Warncke 1966); Adana (Gülek, Pozantı), Amasya, İzmir (Selçuk), Mersin (Sertavul), Nevşehir (Ürgüp) (Warncke 1974); Erzurum (Oltu, Tortum, İspir, Horasan) (Özbek 1976); Aksaray, Ankara, Aydın, Burdur, Çorum, İzmir, Karaman, Konya, Kütahya, Nevşehir, Yozgat (Hazır 2010, Hazır et al. 2014); Antalya, Mersin (this study) (Fig. 59).

#### Flower records

**Brassicaceae**: *Sisymbrium altissimum* L.

### Andrena (Micrandrena) oedicnema

Warncke, 1975

EBF80BB9-B822-5A66-A78B-E216FEFFD49D

#### Materials

- **Type status:** Other material. **Occurrence:** recordedBy: Fatih Dikmen; individualCount: 5; sex: female; occurrenceID: 30D97900-92F3-5D78-8E80-7DEB6CA2E467; **Location:** countryCode: TR; stateProvince: Mersin; county: Mut; locality: Güzlek Plateau; verbatimElevation: 1414 m; verbatimLatitude: 36.654; verbatimLongitude: 33.107; **Identification:** identifiedBy: Thomas J. Wood; **Event:** eventDate: 04-06-2009; **Record Level:** collectionCode: ZMUI
- **Type status:** Other material. **Occurrence:** recordedBy: Fatih Dikmen; individualCount: 1; sex: female; occurrenceID: B1EBF2B9-484A-5C6F-8FBE-759B068FC3FD; **Location:** countryCode: TR; stateProvince: Antalya; locality: Akseki-Konya road; verbatimElevation: 1496 m; verbatimLatitude: 37.14; verbatimLongitude: 31.875; **Identification:** identifiedBy: Thomas J. Wood; **Event:** eventDate: 09-06-2009; **Record Level:** collectionCode: ZMUI

#### Distribution

Greece, Iran, Iraq, Israel and West Bank, Jordan, Lebanon, Syria and Türkiye (Gusenleitner and Schwarz 2002, Pisanty et al. 2018, Wood and Monfared 2022, Wood et al. 2024).

##### Distribution in Türkiye

**Figure 60.**
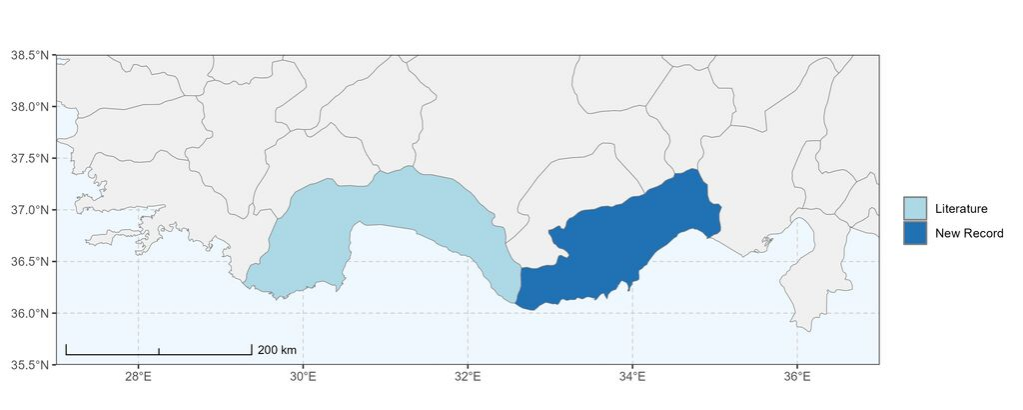
Distribution of *A. oedicnema* in the Mediterranean Region of Türkiye.

Antalya (Akseki), Nevşehir (Warncke 1974); Antalya, Mersin (this study) (Fig. 60).

#### Flower records

**Apiaceae**: *Prangos uechtritzii* Boiss. & Hausskn. **Brassicaceae**: *Isatis* L.

### Andrena (Truncandrena) optata

Warncke, 1975

5F539B1D-1D75-5326-8330-B59BF11FB001

#### Materials

- **Type status:** Other material. **Occurrence:** recordedBy: Fatih Dikmen; individualCount: 2; sex: female; occurrenceID: B8523B85-8F9F-5681-9069-965C64560094; **Location:** countryCode: TR; stateProvince: Antalya; locality: between Side-Belek; verbatimElevation: 16 m; verbatimLatitude: 36.843; verbatimLongitude: 31.292; **Identification:** identifiedBy: Thomas J. Wood; **Event:** eventDate: 13-04-2009; **Record Level:** collectionCode: ZMUI
- **Type status:** Other material. **Occurrence:** recordedBy: Fatih Dikmen; individualCount: 1; sex: female; occurrenceID: 01A6F16A-E56A-5F25-940E-794A32B4787D; **Location:** countryCode: TR; stateProvince: Antalya; locality: Side hotels; verbatimElevation: 13 m; verbatimLatitude: 36.79; verbatimLongitude: 31.399; **Identification:** identifiedBy: Thomas J. Wood; **Event:** eventDate: 13-04-2009; **Record Level:** collectionCode: ZMUI

#### Distribution

Albania, Bulgaria, Greece, Iran, Lebanon, Syria, Türkiye and Ukraine (Wood et al. 2020, Wood 2021b, Wood and Monfared 2022).

##### Distribution in Türkiye

**Figure 61.**
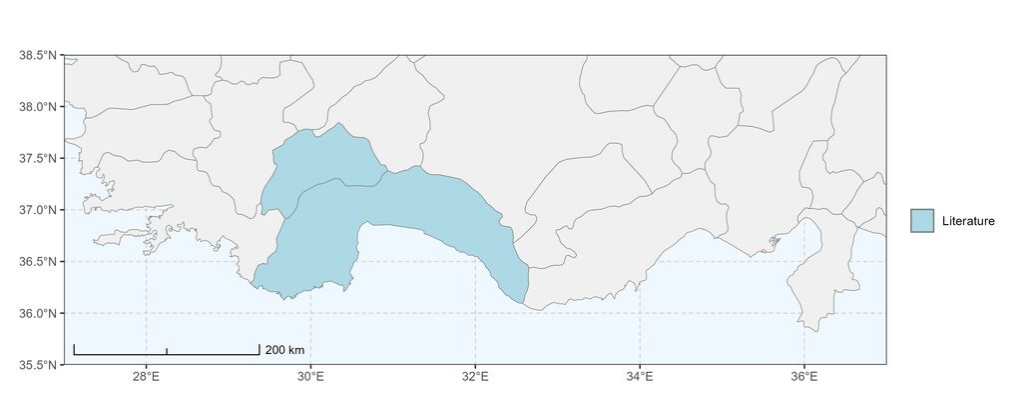
Distribution of *A. optata* in the Mediterranean Region of Türkiye.

Antalya (Serik, Side), Aydın (Kuşadası), Balıkesir (Ayvalık), Burdur (Acıgöl), Bursa, İstanbul (Üsküdar), İzmir (Selçuk), Konya, Manisa (Warncke 1974); Antalya, Aydın, Manisa (Hazır et al. 2014); Antalya (this study) (Fig. 61).

#### Flower records

**Brassicaceae**: *Brassica arvensis* L., *Raphanus raphanistrum* L. (Lanuza et al. 2025); **Brassicaceae**: *Sinapis arvensis* L. **Ranunculaceae**: *Ranunculus marginatus* d’Urv.

### Andrena (Chlorandrena) orientana

Warncke, 1965

CF5BFF19-01E2-5762-97F8-06305431A796

#### Materials

- **Type status:** Other material. **Occurrence:** recordedBy: Fatih Dikmen; individualCount: 4; sex: female; occurrenceID: 3FE642B6-0EFF-5334-9B83-74B37571417A; **Location:** countryCode: TR; stateProvince: Muğla; county: Marmaris; locality: Bördübet road; verbatimElevation: 195 m; verbatimLatitude: 36.857; verbatimLongitude: 28.126; **Identification:** identifiedBy: Thomas J. Wood; **Event:** eventDate: 04-04-2009; **Record Level:** collectionCode: ZMUI

#### Distribution

Bulgaria, Cyprus, Hungary, Iran, Israel and the West Bank, Jordan, Lebanon, North Macedonia, Romania, Syria, Türkiye and Ukraine (Schwenninger 2015, Wood et al. 2020, Wood and Monfared 2022).

##### Distribution in Türkiye

**Figure 62.**
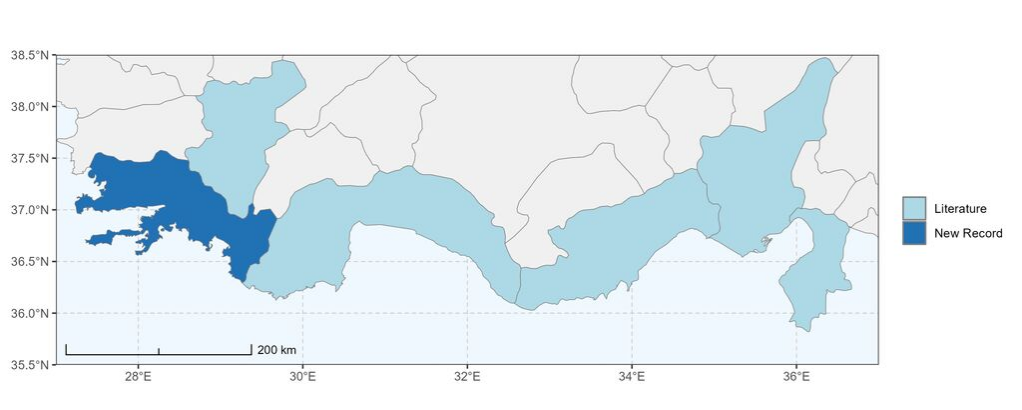
Distribution of *A. orientana* in the Mediterranean Region of Türkiye.

Adana (Misis), Sakarya (Adapazarı), Antalya (Akseki, Manavgat, Side), Balıkesir (Ayvalık), Bursa (İznik), Çanakkale (Bigadiç), Denizli (Pamukkale), Diyarbakır, Hatay, İstanbul (Büyükdere), Manisa, Mersin (Tarsus) (Warncke 1974); Antalya, Aydın, Urfa, Diyarbakır (Hazır 2010, Hazır et al. 2014); Muğla (this study) (Fig. 62).

#### Flower records

**Asteraceae**: *Leontodon tuberosus* L., *Sonchus asper* (L.) Hill (Lanuza et al. 2025); **Asteraceae**: *Crepis* L.

### Andrena (Chlorandrena) panurgimorpha

Mavromoustakis, 1957

B6B163EE-77C1-5B31-9346-19C8C9C2BD33

#### Materials

- **Type status:** Other material. **Occurrence:** recordedBy: Fatih Dikmen; individualCount: 4; sex: female; occurrenceID: D0804C79-2B08-5FF0-9A6F-7410EF8512F3; **Location:** countryCode: TR; stateProvince: Adana; locality: Çukurova University, Balcalı Campus; verbatimElevation: 83 m; verbatimLatitude: 37.049; verbatimLongitude: 35.357; **Identification:** identifiedBy: Thomas J. Wood; **Event:** eventDate: 23-05-2009; **Record Level:** collectionCode: ZMUI
- **Type status:** Other material. **Occurrence:** recordedBy: Fatih Dikmen; individualCount: 2; sex: male; occurrenceID: 8914B8D7-FC02-506E-9634-39111EEB31E8; **Location:** countryCode: TR; stateProvince: Adana; locality: Çukurova University, Balcalı Campus; verbatimElevation: 83 m; verbatimLatitude: 37.049; verbatimLongitude: 35.357; **Identification:** identifiedBy: Thomas J. Wood; **Event:** eventDate: 23-05-2009; **Record Level:** collectionCode: ZMUI

#### Distribution

Albania, Bulgaria, Cyprus, Greece, Iran, Israel and the West Bank, Jordan, Lebanon, Syria, Türkiye and Ukraine (Gusenleitner and Schwarz 2002, Wood et al. 2020, Wood 2021b, Wood and Monfared 2022).

##### Distribution in Türkiye

**Figure 63.**
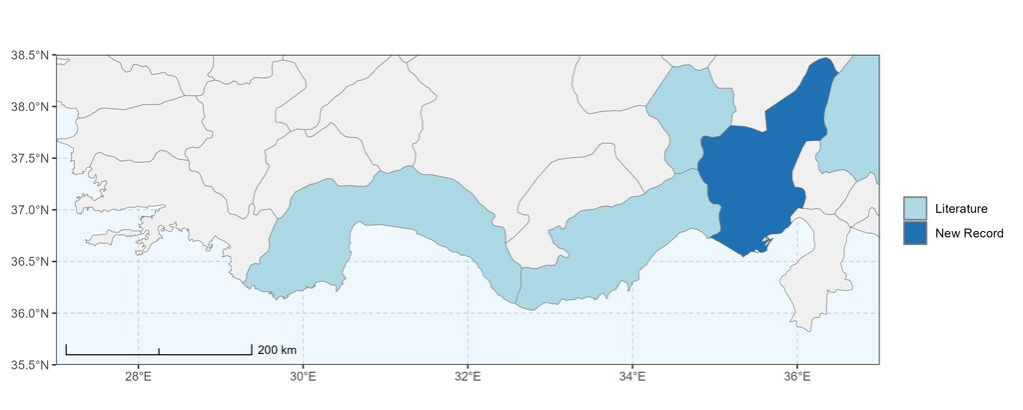
Distribution of *A. panurgimorpha* in the Mediterranean Region of Türkiye.

Balıkesir (Ayvalık), Çanakkale (Truva), Konya (Beyşehir) (Warncke 1966); Erzurum (İspir), Muş (Özbek 1976); Aksaray, Ankara, Antalya, Antep, Aydın, Çorum, Konya, Maraş, Mersin, Nevşehir, Niğde, Rize, Urfa (Hazır 2010); Adana (this study) (Fig. 63).

#### Flower records

**Asteraceae**: *Centaurea* L., *Tragopogon* L. **Fabaceae**: *Medicago sativa* L., *Melilotus officinalis* (L.) Lam. (Khodarahmi Ghahnavieh and Monfared 2019); **Asteraceae**: *Crepis commutata* (Spreng.) Greuter (Lanuza et al. 2025); **Asteraceae**: Crepis foetida subsp. rhoeadifolia (M.Bieb.) Čelak.

### Andrena (Euandrena) pileata canigica

Warncke, 1975

EAA5FD9F-A239-50E7-8021-CAC914C79F62

#### Materials

- **Type status:** Other material. **Occurrence:** recordedBy: Fatih Dikmen; individualCount: 1; sex: male; occurrenceID: 2C1C86EC-CE88-5281-A537-B109A1E2BE30; **Location:** countryCode: TR; stateProvince: Antalya; locality: Akseki-Konya road; verbatimElevation: 1496 m; verbatimLatitude: 37.14; verbatimLongitude: 31.875; **Identification:** identifiedBy: Thomas J. Wood; **Event:** eventDate: 09-06-2009; **Record Level:** collectionCode: ZMUI

#### Distribution

Greece and Türkiye (Wood 2024).

##### Distribution in Türkiye

**Figure 64.**
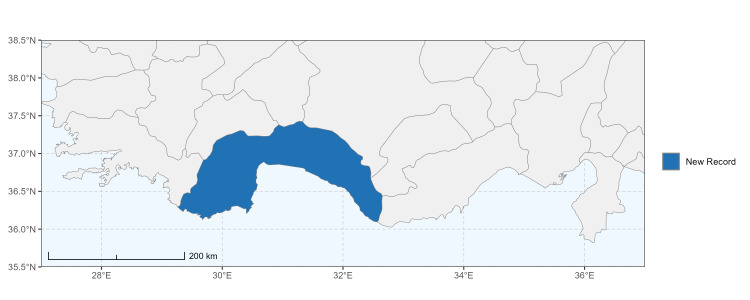
Distribution of A. pileata ssp. canigica in the Mediterranean Region of Türkiye.

**Figure 65.**
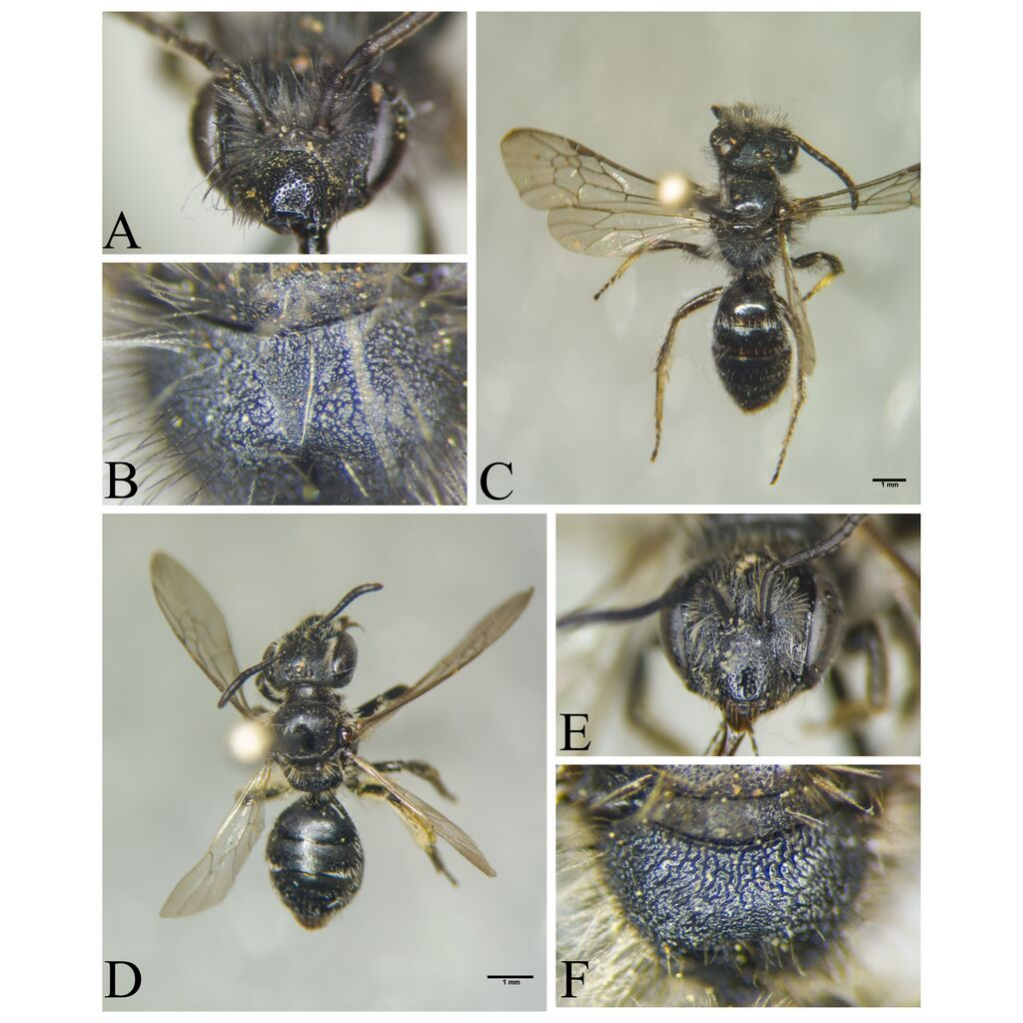
Andrena pileata ssp. canigica, male. Head, frontal view (A); propodeum, dorsal view (B); habitus, dorsal view (C). *Andrena rugulosa*, female. Habitus, dorsal view (D), head, frontal view (E), propodeum, dorsal view (F).

Bursa (Uludağ) (Warncke 1974); Bitlis, Hakkari (Wood 2024); Antalya (this study) (Fig. 64 and Fig. 65A-C).

#### Flower records

**Brassicaceae**: *Isatis* L.

### Andrena (Plastandrena) pilipes

Fabricius, 1781

7C59234C-51AE-5A50-975B-64A55BF9A0A9

#### Materials

- **Type status:** Other material. **Occurrence:** recordedBy: Fatih Dikmen; individualCount: 1; sex: female; occurrenceID: 79F265F9-C5CE-515B-8D0D-22058FD33ECF; **Location:** countryCode: TR; stateProvince: Antalya; county: Gazipaşa; locality: Beyrebucak; verbatimElevation: 93 m; verbatimLatitude: 36.216; verbatimLongitude: 32.392; **Identification:** identifiedBy: Thomas J. Wood; **Event:** eventDate: 14-04-2009; **Record Level:** collectionCode: ZMUI

#### Distribution

Afghanistan, Albania, Andorra, Algeria, Austria, Belgium, Bulgaria, China, Corsica, Croatia, Denmark, Finland, France, Germany, Great Britain, Hungary, Ireland, Iran, Italy, Kazakhstan, Latvia, Lithuania, Libya, Luxembourg, Moldova, Monaco, Morocco, Netherlands, Norway, Pakistan, Poland, Portugal, Romania, Russia, San Marino, Slovakia, Spain, Sweden, Switzerland, Tunisia, Turkmenistan, Türkiye, Ukraine and Uzbekistan (Gusenleitner and Schwarz 2002, Wood and Monfared 2022, Ascher and Pickering 2025).

##### Distribution in Türkiye

**Figure 66.**
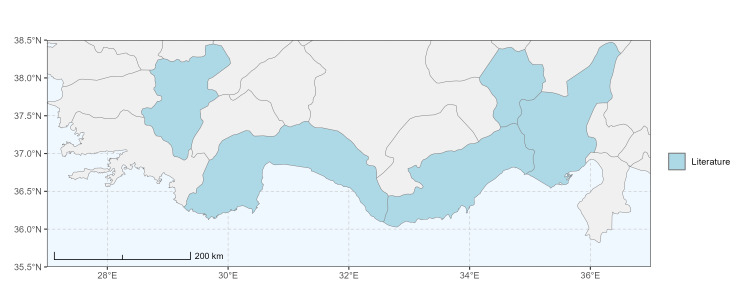
Distribution of *A. pilipes* in the Mediterranean Region of Türkiye.

Denizli (Dazkırı) (Warncke 1969); Adana, Aksaray, Ankara, Antalya, Aydın, İzmir, Kırşehir, Konya, Mersin, Niğde (Hazır 2010, Hazır et al. 2014); Antalya (this study) (Fig. 66).

#### Flower records

**Asteraceae**: *Senecio jacobaea* L. (Balfour et al. 2022); **Asparagaceae**: *Asparagus officinalis* L. **Asteraceae**: *Centaurea spinosa* L., *Glebionis coronaria* (L.) Cass. ex Spach, *Leontodon tuberosus* L. **Boraginaceae**: *Echium angustifolium* Mill. **Brassicaceae**: *Bunias orientalis* L., *Hirschfeldia incana* (L.) Lagr.-Foss., *Sisymbrium loeselii* L., *Sisymbrium loeselii* L., *Sisymbrium loeselii* L., *Spiraea* L. **Crassulaceae**: *Petrosedum sediforme* (Jacq.) Grulich **Ericaceae**: *Erica arborea* L., *Rhododendron* L. **Fabaceae**: *Adenocarpus hispanicus* (Lam.) DC. **Lamiaceae**: *Thymbra capitata* (L.) Cav., *Thymus praecox* Opiz **Oxalidaceae**: *Oxalis articulata* Savigny **Papaveraceae**: *Glaucium flavum* Crantz **Rosaceae**: *Crataegus azarolus* L., *Crataegus monogyna* Jacq., *Cydonia oblonga* Mill., *Photinia* Lindl., *Prunus laurocerasus* L. (Lanuza et al. 2025); **Brassicaceae**: *Sinapis arvensis* L.

### Andrena (Notandrena) purpureomicans

Alfken, 1935

9BBE18D5-8519-570E-B387-DE82A3AAF2D3

#### Materials

- **Type status:** Other material. **Occurrence:** recordedBy: Fatih Dikmen; individualCount: 1; sex: female; occurrenceID: 12E75408-842E-522A-9BA5-EEA5FAB3A228; **Location:** countryCode: TR; stateProvince: Antalya; locality: Burdur road, Korkuteli turn; verbatimElevation: 783 m; verbatimLatitude: 37.313; verbatimLongitude: 30.49; **Identification:** identifiedBy: Thomas J. Wood; **Event:** eventDate: 29-05-2009; **Record Level:** collectionCode: ZMUI

#### Distribution

Türkiye (Gusenleitner and Schwarz 2002).

##### Distribution in Türkiye

**Figure 67.**
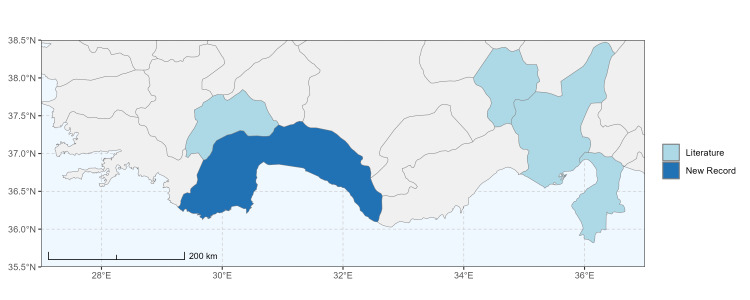
Distribution of *A. purpureomicans* in the Mediterranean Region of Türkiye.

Adana, Ankara, Konya, Hatay (Warncke 1966); Niğde (Ulukışla) (Warncke 1974); Erzurum (İspir), Muş (Özbek 1976); Aksaray, Ankara, Burdur, Konya, Kütahya, Manisa (Hazır 2010, Hazır et al. 2014); Antalya (this study) (Fig. 67).

#### Flower records

**Brassicaceae**: *Hirschfeldia incana* (L.) Lagr.-Foss.

### Andrena (Melandrena) pyropygia

Kreichbaumer, 1873

CA9B71FB-5E0A-5D58-8DE0-106869B6C5E1

#### Materials

- **Type status:** Other material. **Occurrence:** recordedBy: Fatih Dikmen; individualCount: 1; sex: female; occurrenceID: 56DD180C-5BF6-5809-9B37-2720796C9556; **Location:** countryCode: TR; stateProvince: Karaman; locality: Mut-Ermenek road; verbatimElevation: 1023 m; verbatimLatitude: 36.63; verbatimLongitude: 33.011; **Identification:** identifiedBy: Thomas J. Wood; **Event:** eventDate: 05-06-2009; **Record Level:** collectionCode: ZMUI
- **Type status:** Other material. **Occurrence:** recordedBy: Fatih Dikmen; individualCount: 1; sex: male; occurrenceID: 815DDB1F-7F5C-5CFB-8CF3-2DA27D78E6E1; **Location:** countryCode: TR; stateProvince: Hatay; locality: between Yayladağ and Samandağ; verbatimElevation: 450 m; verbatimLatitude: 35.923; verbatimLongitude: 36.054; **Identification:** identifiedBy: Thomas J. Wood; **Event:** eventDate: 27-04-2008; **Record Level:** collectionCode: ZMUI

#### Distribution

Cyprus, Greece, Iran, Israel and the West Bank, Lebanon, Russia, Syria, the Caucasus, Türkiye and Ukraine (Gusenleitner and Schwarz 2002, Wood et al. 2020, Wood and Monfared 2022).

##### Distribution in Türkiye

**Figure 68.**
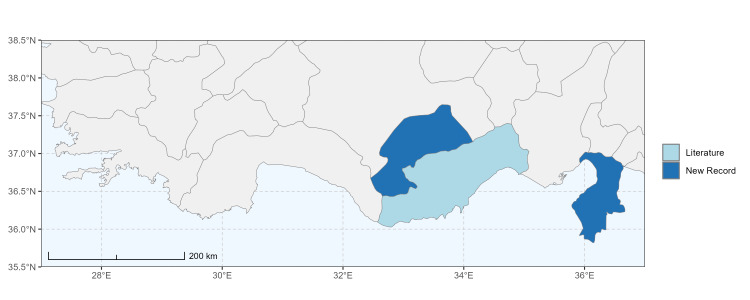
Distribution of *A. pyropygia* in the Mediterranean Region of Türkiye.

Amanos Mountains, Amasya, Bursa, Toros Mountains (Warncke 1966); Aydın (Kuşadası) (Warncke 1969); Aydın, Çorum, Mersin (Hazır 2010, Hazır et al. 2014); Hatay, Karaman (this study) (Fig. 68).

#### Flower records

**Resedaceae**: Reseda lutea var. lutea L.

### Andrena (Truncandrena) rufomaculata

Friese, 1921

0E99AD27-BB33-55DB-A712-3E6FA7BF4B7A

#### Materials

- **Type status:** Other material. **Occurrence:** recordedBy: Fatih Dikmen; individualCount: 1; sex: male; occurrenceID: FA74A199-1B63-5775-A765-EA2A5CDB507A; **Location:** countryCode: TR; stateProvince: Hatay; locality: between Kırıkhan and Hassa; verbatimElevation: 131 m; verbatimLatitude: 36.564; verbatimLongitude: 36.402; **Identification:** identifiedBy: Thomas J. Wood; **Event:** eventDate: 22-03-2009; **Record Level:** collectionCode: ZMUI

#### Distribution

Iran, Iraq, Israel and West Bank, Jordan, Lebanon, Syria and Türkiye (Wood et al. 2020, Wood and Monfared 2022, Wood et al. 2024).

##### Distribution in Türkiye

**Figure 69.**
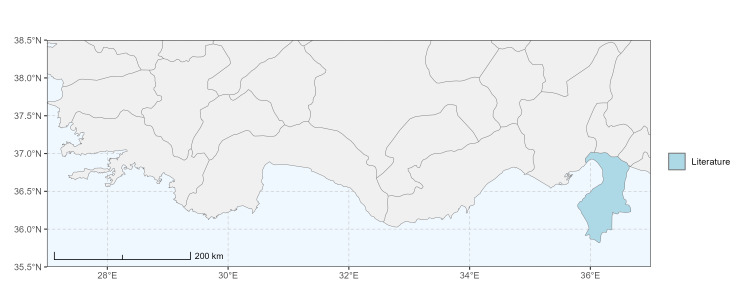
Distribution of *A. rufomaculata* in the Mediterranean Region of Türkiye.

Amanos Mountains (Warncke 1966); Hatay (Hazır 2010, Hazır et al. 2014); Hatay (this study) (Fig. 69).

#### Flower records

**Brassicaceae**: *Sisymbrium* L.

### Andrena (Euandrena) rufula

Schmiedeknecht, 1883

05804450-035D-52CB-B905-68879AB8F846

#### Materials

- **Type status:** Other material. **Occurrence:** recordedBy: Fatih Dikmen; individualCount: 4; sex: female; occurrenceID: E15A5BB1-4FD4-5740-A6B2-E37668098ABC; **Location:** countryCode: TR; stateProvince: Hatay; locality: Yayladağ; verbatimElevation: 423 m; verbatimLatitude: 35.908; verbatimLongitude: 36.078; **Identification:** identifiedBy: Thomas J. Wood; **Event:** eventDate: 22-03-2009; **Record Level:** collectionCode: ZMUI

#### Distribution

Austria, France, Greece, Hungary, Israel and the West Bank, Italy, Lebanon, Netherlands, North Macedonia, Romania, Slovakia, Spain, Switzerland, Syria, Türkiye and Ukraine (Gusenleitner and Schwarz 2002, Scheuchl and Willner 2016, Wood et al. 2020, Pisanty et al. 2022).

##### Distribution in Türkiye

**Figure 70.**
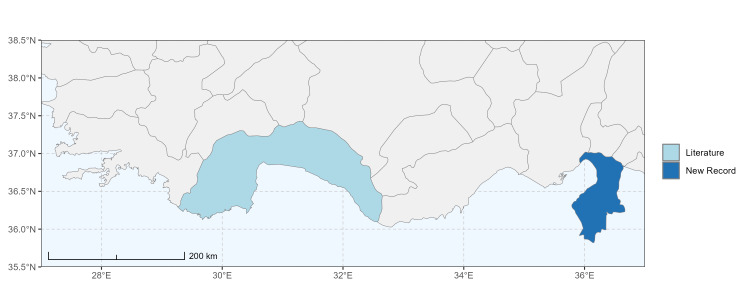
Distribution of *A. rufula* in the Mediterranean Region of Türkiye.

Toroslar (Warncke 1974); Antalya (Pisanty et al. 2022); Hatay (this study) (Fig. 70).

#### Notes

##### Flower records

**Buxaceae**: *Buxus sempervirens* L. **Plantaginaceae**: *Globularia vulgaris* L. **Rosaceae**: *Pyrus communis* L. (Lanuza et al. 2025); **Asteraceae**: *Senecio vernalis* Waldst. & Kit.

### Andrena (Micrandrena) rugothorace

Warncke, 1965

5FC0FF44-7AF1-51F2-AAE6-52F8399F62C0

#### Materials

- **Type status:** Other material. **Occurrence:** recordedBy: Fatih Dikmen; individualCount: 1; sex: female; occurrenceID: 21B1FBDD-310F-5027-BC6B-C83455949280; **Location:** countryCode: TR; stateProvince: Adana; locality: Çukurova University, Balcalı Campus; verbatimElevation: 145 m; verbatimLatitude: 37.065; verbatimLongitude: 35.366; **Identification:** identifiedBy: Thomas J. Wood; **Event:** eventDate: 20-03-2009; **Record Level:** collectionCode: ZMUI
- **Type status:** Other material. **Occurrence:** recordedBy: Fatih Dikmen; individualCount: 5; sex: female; occurrenceID: 1E22372D-3CE0-59D9-BA04-0C81CEBD4600; **Location:** countryCode: TR; stateProvince: Muğla; county: Marmaris; verbatimElevation: 9 m; verbatimLatitude: 36.985; verbatimLongitude: 28.216; **Identification:** identifiedBy: Thomas J. Wood; **Event:** eventDate: 02-04-2009; **Record Level:** collectionCode: ZMUI
- **Type status:** Other material. **Occurrence:** recordedBy: Fatih Dikmen; individualCount: 1; sex: female; occurrenceID: BA6D44FC-8E19-5F7B-BC39-7A6EAADF67AA; **Location:** countryCode: TR; stateProvince: Muğla; county: Datça; verbatimElevation: 1 m; verbatimLatitude: 36.802; verbatimLongitude: 27.866; **Identification:** identifiedBy: Thomas J. Wood; **Event:** eventDate: 04-04-2009; **Record Level:** collectionCode: ZMUI
- **Type status:** Other material. **Occurrence:** recordedBy: Fatih Dikmen; individualCount: 1; sex: female; occurrenceID: DC425280-782A-5CFE-B3BE-6C58441C1F98; **Location:** countryCode: TR; stateProvince: Antalya; locality: Akseki-Konya road; verbatimElevation: 1262 m; verbatimLatitude: 37.117; verbatimLongitude: 31.777; **Identification:** identifiedBy: Thomas J. Wood; **Event:** eventDate: 09-06-2009; **Record Level:** collectionCode: ZMUI
- **Type status:** Other material. **Occurrence:** recordedBy: Fatih Dikmen; individualCount: 1; sex: female; occurrenceID: 28D32FB9-9375-5D70-9530-BFF33D9C033E; **Location:** countryCode: TR; stateProvince: Antalya; county: Gazipaşa; locality: Zeytinada; verbatimElevation: 379 m; verbatimLatitude: 36.144; verbatimLongitude: 32.471; **Identification:** identifiedBy: Thomas J. Wood; **Event:** eventDate: 14-04-2009; **Record Level:** collectionCode: ZMUI

#### Distribution

Albania, Armenia, Azerbaijan, Georgia, Greece, Israel and the West Bank, North Macedonia and Türkiye (Gusenleitner and Schwarz 2002, Wood 2024).

##### Distribution in Türkiye

**Figure 71.**
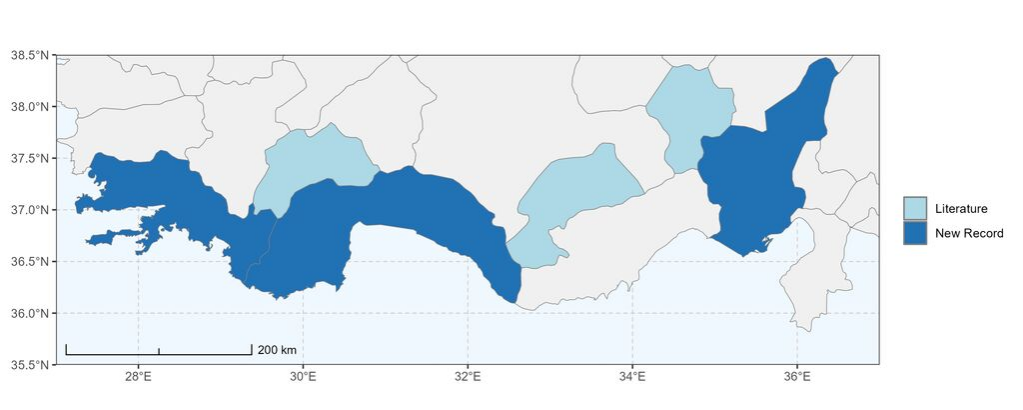
Distribution of *A. rugothorace* in the Mediterranean Region of Türkiye.

Ankara (Gölbaşı), Balıkesir (Ayvalık), Bolu (Gerede), Bursa (Karacabey), Burdur, Diyarbakır, Edirne, Erzurum (İspir), İstanbul (Belgrad Ormanı, Büyükdere, Üsküdar), Karaman (Madenşehri), Nevşehir, Niğde (Ulukışla) (Warncke 1974); Adana, Antalya, Muğla (this study) (Fig. 71).

#### Flower records

**Apiaceae**: *Pimpinella peregrina* L. **Asteraceae**: *Tripleurospermum* Sch.Bip. (Lanuza et al. 2025); **Asteraceae**: *Anthemis chia* L., *Anthemis pseudocotula* Boiss., *Calendula arvensis* L. **Cistaceae**: Cistus L.

### Andrena (Micrandrena) rugulosa

Stöckhert, 1935

BF950FD3-E85A-5B65-BE8B-7327CD047AD9

#### Materials

- **Type status:** Other material. **Occurrence:** recordedBy: Fatih Dikmen; individualCount: 1; sex: female; occurrenceID: 0E183282-C210-5BCA-A789-A8D6E7E5A0F4; **Location:** countryCode: TR; stateProvince: Niğde; locality: Çamardı road; verbatimElevation: 1570 m; verbatimLatitude: 37.886; verbatimLongitude: 35.111; **Identification:** identifiedBy: Thomas J. Wood; **Event:** eventDate: 23-05-2008; **Record Level:** collectionCode: ZMUI

#### Distribution

Albania, Austria, Bulgaria, Croatia, Czechia, France, Germany, Greece, Iran, Italy, Lebanon, North Macedonia, Poland, Romania, Slovakia, Slovenia, the Caucasus and Türkiye (Gusenleitner and Schwarz 2002, Wood and Monfared 2022).

##### Distribution in Türkiye

**Figure 72.**
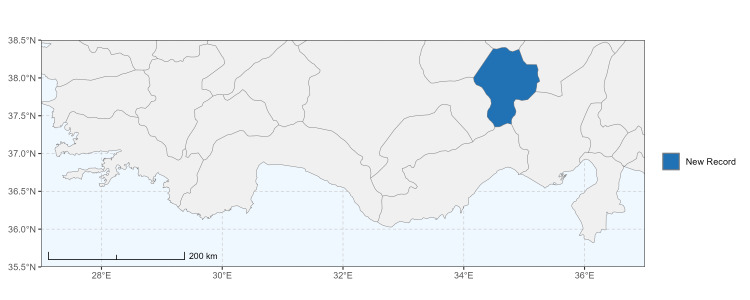
Distribution of *A. rugulosa* in the Mediterranean Region of Türkiye.

Erzurum (Warncke 1974); Niğde (this study) (Fig. 65D-F and Fig. 72).

#### Flower records

**Brassicaceae**: *Brassica* L.

### Andrena (Taeniandrena) russula

Lepeletier, 1841

36360F6F-4DD4-50F2-A80D-7325D329C804

#### Materials

- **Type status:** Other material. **Occurrence:** recordedBy: Fatih Dikmen; individualCount: 1; sex: female; occurrenceID: B9BD5383-60FC-5AD2-8F18-4B754F44B212; **Location:** countryCode: TR; stateProvince: Muğla; county: Datça; verbatimElevation: 1 m; verbatimLatitude: 36.802; verbatimLongitude: 27.866; **Identification:** identifiedBy: Thomas J. Wood; **Event:** eventDate: 04-04-2009; **Record Level:** collectionCode: ZMUI
- **Type status:** Other material. **Occurrence:** recordedBy: Fatih Dikmen; individualCount: 1; sex: female; occurrenceID: DBE64CC4-5CA9-5EA0-9B3E-BDF6AF6EA8CD; **Location:** countryCode: TR; stateProvince: Muğla; county: Marmaris; verbatimElevation: 9 m; verbatimLatitude: 36.985; verbatimLongitude: 28.216; **Identification:** identifiedBy: Thomas J. Wood; **Event:** eventDate: 02-04-2009; **Record Level:** collectionCode: ZMUI

#### Distribution

Albania, Algeria, Armenia, Austria, Azerbaijan, Belarus, Belgium, Bosnia and Herzegovina, Bulgaria, Croatia, Czechia, Denmark, Estonia, Finland, France, Georgia, Germany, Great Britain, Greece, Hungary, Ireland, Israel and the West Bank, Italy, Jordan, Latvia, Lebanon, Lithuania, Luxembourg, Montenegro, Morocco, Netherlands, North Macedonia, Norway, Poland, Portugal, Romania, Russia, Serbia, Slovakia, Slovenia, Spain, Sweden, Switzerland, Syria, Tunisia, Türkiye and Ukraine (Gusenleitner and Schwarz 2002, Praz et al. 2022, Wood and Monfared 2022).

##### Distribution in Türkiye

**Figure 73.**
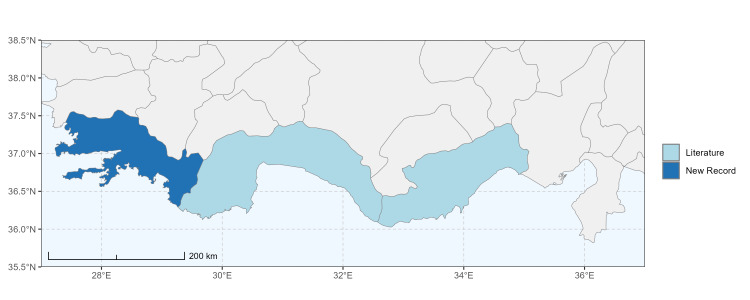
Distribution of *A. russula* in the Mediterranean Region of Türkiye.

Ankara (Kızılcahamam), Antalya (Akseki), Balıkesir (Ayvalık), Bolu, Erzurum, Konya (Beyşehir), Manisa (Akhisar), Mersin (Gülek) (Warncke 1974); Muğla (this study) (Fig. 73).

#### Flower records

**Cistaceae**: *Cistus* L.

### Andrena (Opandrena) schencki

Morawitz, 1866

0EE7005E-2421-5056-B989-A45704DD7F4B

#### Materials

- **Type status:** Other material. **Occurrence:** recordedBy: Fatih Dikmen; individualCount: 1; sex: male; occurrenceID: 07AA8994-9592-5E35-A34D-A4F752E67E3E; **Location:** countryCode: TR; stateProvince: Muğla; county: Datça; locality: Ballık hill; verbatimElevation: 0 m; verbatimLatitude: 36.808; verbatimLongitude: 27.901; **Identification:** identifiedBy: Thomas J. Wood; **Event:** eventDate: 04-04-2009; **Record Level:** collectionCode: ZMUI

#### Distribution

Armenia, Austria, Azerbaijan, Belarus, Belgium, Bosnia and Herzegovina, Bulgaria, Croatia, Czechia, Denmark, France, Georgia, Germany, Great Britain, Greece, Hungary, Italy, Latvia, Lithuania, Luxembourg, Netherlands, Poland, Portugal, Romania, Russia, Serbia, Slovakia, Slovenia, Spain, Sweden, Switzerland, Syria, Turkmenistan, Türkiye and Ukraine (Gusenleitner and Schwarz 2002, Osytshnjuk et al. 2008, Wood and Monfared 2022).

##### Distribution in Türkiye

**Figure 74.**
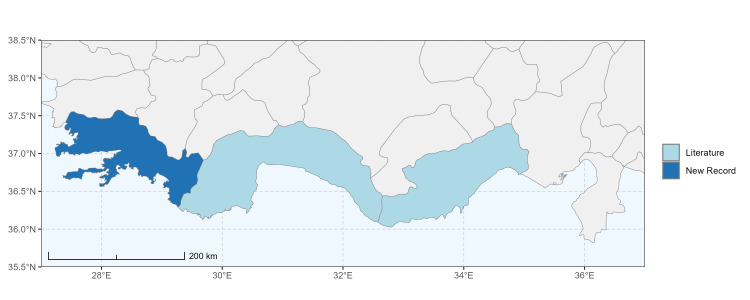
Distribution of *A. schencki* in the Mediterranean Region of Türkiye.

Mersin (Namrun) (Warncke 1969); Amanos Mountains, Balıkesir (Ayvalık), Konya (Beyşehir), Erzurum (Oltu, Hınıs, Pasinler, Horasan) (Özbek 1976); Ankara, Antalya, Aydın, Balıkesir, Çankırı, Çorum, Konya, Kütahya, Mersin (Hazır 2010, Hazır et al. 2014); Muğla (this study) (Fig. 74).

#### Flower records

**Fabaceae**: *Trifolium pratense* L. (Lanuza et al. 2025); **Asteraceae**: *Glebionis segetum* (L.) Fourr.

### Andrena (Truncandrena) schmiedeknechti

Magretti, 1883

EA7895C5-F759-5B0A-9F64-A014A31C0034

#### Materials

- **Type status:** Other material. **Occurrence:** recordedBy: Fatih Dikmen; individualCount: 1; sex: female; occurrenceID: 22570572-90D6-5C23-B2D2-2F23AA34F224; **Location:** countryCode: TR; stateProvince: Mersin; county: Mut; locality: Güzlek Plateau; verbatimElevation: 1414 m; verbatimLatitude: 36.654; verbatimLongitude: 33.107; **Identification:** identifiedBy: Thomas J. Wood; **Event:** eventDate: 04-06-2009; **Record Level:** collectionCode: ZMUI

#### Distribution

Algeria, Armenia, Azerbaijan, Georgia, Greece, Italy, Morocco, Tunisia,and Türkiye (Gusenleitner and Schwarz 2002).

##### Distribution in Türkiye

**Figure 75.**
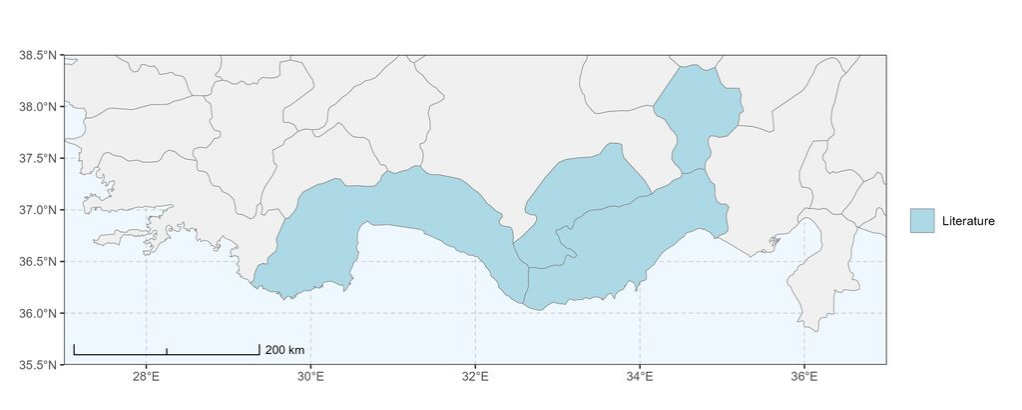
Distribution of *A. schmiedeknechti* in the Mediterranean Region of Türkiye.

Balıkesir (Ayvalık), Sakarya (Adapazarı) (Warncke 1966); Bursa (Mustafakemalpaşa), Çanakkale (Bigadiç), İzmir (Selçuk), Kars (Sarıkamış), Karaman (Madenşehri), Kayseri (Yenişehir), Manisa, Nevşehir (Ürgüp), Niğde (Ulukışla) (Warncke 1974); Ankara, Antalya, Aydın, Karaman, Konya, Mersin (Hazır 2010, Hazır et al. 2014); Mersin (this study) (Fig. 75).

#### Flower records

**Brassicaceae**: *Sisymbrium altissimum* L.

### Andrena (Scitandrena) scita

Eversmann, 1852

B687770D-EEBD-5145-9317-EFDAD483A58C

#### Materials

- **Type status:** Other material. **Occurrence:** recordedBy: Fatih Dikmen; individualCount: 2; sex: female; occurrenceID: CDE10BDB-C4C8-50B7-B11D-C55E6ECCF461; **Location:** countryCode: TR; stateProvince: Karaman; locality: Mut-Ermenek road; verbatimElevation: 1023 m; verbatimLatitude: 36.63; verbatimLongitude: 33.011; **Identification:** identifiedBy: Thomas J. Wood; **Event:** eventDate: 05-06-2009; **Record Level:** collectionCode: ZMUI
- **Type status:** Other material. **Occurrence:** recordedBy: Fatih Dikmen; individualCount: 2; sex: male; occurrenceID: 947F33CD-11F9-5B23-87CF-BDBFC673AB26; **Location:** countryCode: TR; stateProvince: Karaman; locality: Mut-Ermenek road; verbatimElevation: 1023 m; verbatimLatitude: 36.63; verbatimLongitude: 33.011; **Identification:** identifiedBy: Thomas J. Wood; **Event:** eventDate: 05-06-2009; **Record Level:** collectionCode: ZMUI
- **Type status:** Other material. **Occurrence:** recordedBy: Fatih Dikmen; individualCount: 1; sex: female; occurrenceID: 232D8AA0-49E1-5F05-94B7-96F166FFFF36; **Location:** countryCode: TR; stateProvince: Afyon; county: Dazkırı; locality: Örtülü village exit; verbatimElevation: 891 m; verbatimLatitude: 37.899; verbatimLongitude: 29.753; **Identification:** identifiedBy: Thomas J. Wood; **Event:** eventDate: 12-06-2009; **Record Level:** collectionCode: ZMUI

#### Distribution

Albania, Armenia, Austria, Azerbaijan, Bosnia and Herzegovina, Bulgaria, Croatia, Czechia, Georgia, Greece, Hungary, Iran, Israel and the West Bank, Italy, Jordan, Lebanon, North Macedonia, Romania, Russia, Serbia, Slovakia, Slovenia, Syria, Turkmenistan, Türkiye and Ukraine (Gusenleitner and Schwarz 2002, Wood and Monfared 2022).

##### Distribution in Türkiye

**Figure 76.**
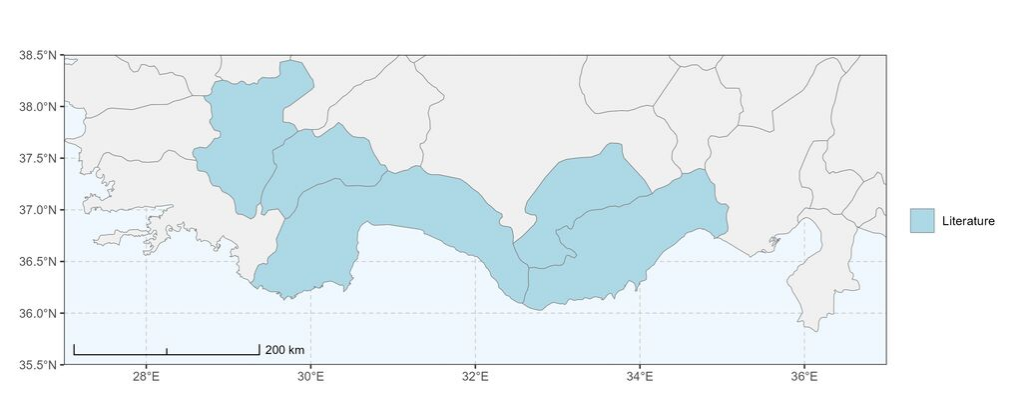
Distribution of *A. scita* in the Mediterranean Region of Türkiye.

Aksaray, Ankara, Bilecik, Çanakkale (Truva), Denizli (Çardak, Pamukkale), Kayseri (Erciyes), Konya (Beyşehir), Toroslar (Warncke 1966); Erzurum (Oltu, Tortum, İspir, Horasan, Hınıs) (Özbek 1976); Aksaray, Ankara, Antalya, Aydın, Balıkesir, Burdur, Çanakkale, Denizli, Karabük, Karaman, Kırşehir, Kütahya, Mersin, Nevşehir (Hazır 2010, Hazır et al. 2014); Afyon, Karaman (this study) (Fig. 76).

#### Flower records

**Brassicaceae**: *Cakile maritima* Scop., *Hirschfeldia incana* (L.) Lagr.-Foss. (Lanuza et al. 2025); **Brassicaceae**: *Brassica elongata* Ehrh., *Erysimum crassipes* Fisch. & C.A.Mey.

### Andrena (Micrandrena) spreta

Pérez, 1895

98114135-65E0-5934-9B95-F43E4DF4BB74

#### Materials

- **Type status:** Other material. **Occurrence:** recordedBy: Fatih Dikmen; individualCount: 1; sex: female; occurrenceID: EFF39067-B513-5581-BB1E-DF9E8C0A271D; **Location:** countryCode: TR; stateProvince: Hatay; locality: Belen Pass; verbatimElevation: 274 m; verbatimLatitude: 36.46; verbatimLongitude: 36.279; **Identification:** identifiedBy: Thomas J. Wood; **Event:** eventDate: 21-03-2009; **Record Level:** collectionCode: ZMUI
- **Type status:** Other material. **Occurrence:** recordedBy: Fatih Dikmen; individualCount: 1; sex: male; occurrenceID: 5B371ECD-FBA6-5A12-B1A6-9FEB6C214775; **Location:** countryCode: TR; stateProvince: Hatay; locality: Belen Pass; verbatimElevation: 274 m; verbatimLatitude: 36.46; verbatimLongitude: 36.279; **Identification:** identifiedBy: Thomas J. Wood; **Event:** eventDate: 21-03-2009; **Record Level:** collectionCode: ZMUI
- **Type status:** Other material. **Occurrence:** recordedBy: Fatih Dikmen; individualCount: 4; sex: female; occurrenceID: 2A8D4DDA-4346-5B67-89C5-EFE2CAB4176F; **Location:** countryCode: TR; stateProvince: Hatay; locality: between Harbiye-Yayladağ; verbatimElevation: 471 m; verbatimLatitude: 36.123; verbatimLongitude: 36.171; **Identification:** identifiedBy: Thomas J. Wood; **Event:** eventDate: 21-03-2009; **Record Level:** collectionCode: ZMUI
- **Type status:** Other material. **Occurrence:** recordedBy: Fatih Dikmen; individualCount: 3; sex: female; occurrenceID: 479E7653-3392-5074-B5EA-F110A26AAE22; **Location:** countryCode: TR; stateProvince: Antalya; locality: Side Hotels; verbatimElevation: 13 m; verbatimLatitude: 36.79; verbatimLongitude: 31.399; **Identification:** identifiedBy: Thomas J. Wood; **Event:** eventDate: 13-04-2009; **Record Level:** collectionCode: ZMUI
- **Type status:** Other material. **Occurrence:** recordedBy: Fatih Dikmen; individualCount: 4; sex: female; occurrenceID: 0D17F19C-C904-57A2-AED5-606FDFF97501; **Location:** countryCode: TR; stateProvince: Mersin; county: Anamur; verbatimElevation: 21 m; verbatimLatitude: 36.092; verbatimLongitude: 32.808; **Identification:** identifiedBy: Thomas J. Wood; **Event:** eventDate: 06-06-2009; **Record Level:** collectionCode: ZMUI

#### Distribution

Afghanistan, Albania, Algeria, Azerbaijan, Belgium, Croatia, Cyprus, Egypt, France, Greece, Iran, Israel and the West Bank, Italy, Libya, Morocco, Spain, Syria, Tunisia and Türkiye (Gusenleitner and Schwarz 2002, Ascher and Pickering 2025).

##### Distribution in Türkiye

**Figure 77.**
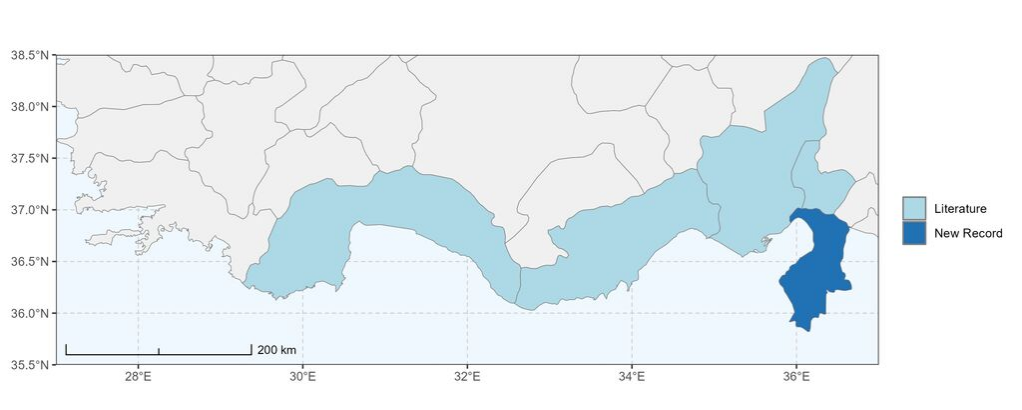
Distribution of *A. spreta* in the Mediterranean Region of Türkiye.

Adana, Antep, Antalya, Diyarbakır, Malatya, Mersin (Mut, Silifke, Tarsus), Osmaniye, Urfa (Warncke 1974); Antalya, Hatay, Mersin (this study) (Fig. 77).

#### Flower records

**Apiaceae**: *Elaeoselinum* W.D.J.Koch ex DC. (Lanuza et al. 2025); **Brassicaceae**: *Hirschfeldia incana* (L.) Lagr.-Foss., *Barbarea plantaginea* DC., *Sisymbrium* L.

### Andrena (Euandrena) symphyti

Schmiedeknecht, 1883

491DC995-48F1-5B0D-9511-E00904B73E7E

#### Materials

- **Type status:** Other material. **Occurrence:** recordedBy: Fatih Dikmen; individualCount: 2; sex: female; occurrenceID: BBD6BE77-07F0-587A-A89C-C607E7700554; **Location:** countryCode: TR; stateProvince: Niğde; county: Çamardı; locality: Pınarbaşı; verbatimElevation: 1512 m; verbatimLatitude: 37.878; verbatimLongitude: 35.107; **Identification:** identifiedBy: Thomas J. Wood; **Event:** eventDate: 23-05-2008; **Record Level:** collectionCode: ZMUI

#### Distribution

Albania, Armenia, Austria, Azerbaijan, Bosnia and Herzegovina, Bulgaria, Croatia, Czechia, France, Georgia, Germany, Gibraltar, Greece, Iran, Iraq, Italy, Moldova, Poland, Romania, Russia, Serbia, Slovakia, Spain, Switzerland, Türkiye and Ukraine (Gusenleitner and Schwarz 2002, Wood and Monfared 2022, Pisanty et al. 2022, Wood et al. 2024, Ascher and Pickering 2025).

##### Distribution in Türkiye

**Figure 78.**
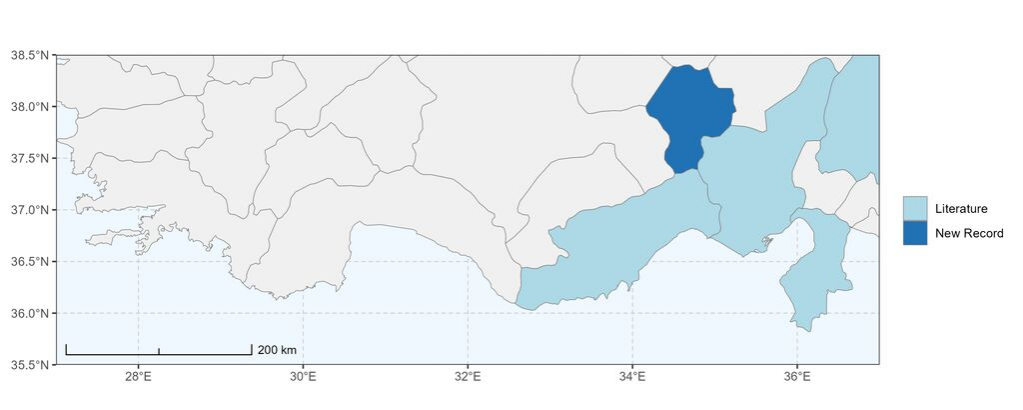
Distribution of *A. symphyti* in the Mediterranean Region of Türkiye.

Adana (Topraklı), Ankara, Erzurum (Horasan, Oltu), Gümüşhane, Hatay, İstanbul, Karaman (Madenşehri), Konya (Akşehir, Sarayönü), Mardin (Tanyeri), Mersin (Mut, Gülek), Nevşehir (Ürgüp), Rize (Çinçiva) (Warncke 1974); Erzurum (Özbek 1976); Aydın (Hazır 2010, Hazır et al. 2014); Kahramanmaraş (Pisanty et al. 2022); Niğde (this study) (Fig. 78).

### Andrena (Taeniandrena) taedium

Wood, 2023

0AC5DCEC-B2A9-5904-84A7-B260B55CAE71

#### Materials

- **Type status:** Other material. **Occurrence:** recordedBy: Fatih Dikmen; individualCount: 1; sex: female; occurrenceID: F276417C-530B-5D55-94B8-8D849D2E1798; **Location:** countryCode: TR; stateProvince: Muğla; locality: Datça road, Turunç; verbatimElevation: 347 m; verbatimLatitude: 36.774; verbatimLongitude: 28.22; **Identification:** identifiedBy: Thomas J. Wood; **Event:** eventDate: 03-04-2009; **Record Level:** collectionCode: ZMUI
- **Type status:** Other material. **Occurrence:** recordedBy: Fatih Dikmen; individualCount: 2; sex: female; occurrenceID: 4EEBCC15-ACB5-5BCE-830C-1ACFB55E4ED5; **Location:** countryCode: TR; stateProvince: Niğde; county: Pozantı; locality: Alihoca village exit; verbatimElevation: 1171 m; verbatimLatitude: 37.483; verbatimLongitude: 34.705; **Identification:** identifiedBy: Thomas J. Wood; **Event:** eventDate: 23-05-2009; **Record Level:** collectionCode: ZMUI
- **Type status:** Other material. **Occurrence:** recordedBy: Fatih Dikmen; individualCount: 1; sex: female; occurrenceID: DBC1ACAC-3318-52CB-9A82-1917FD7B38A4; **Location:** countryCode: TR; stateProvince: Antalya; locality: Saklıkent road, 4 km; verbatimElevation: 1759 m; verbatimLatitude: 36.87; verbatimLongitude: 30.343; **Identification:** identifiedBy: Thomas J. Wood; **Event:** eventDate: 11-06-2009; **Record Level:** collectionCode: ZMUI

#### Distribution

Greece, Iran, Lebanon and Türkiye (Wood 2023a).

##### Distribution in Türkiye

**Figure 79.**
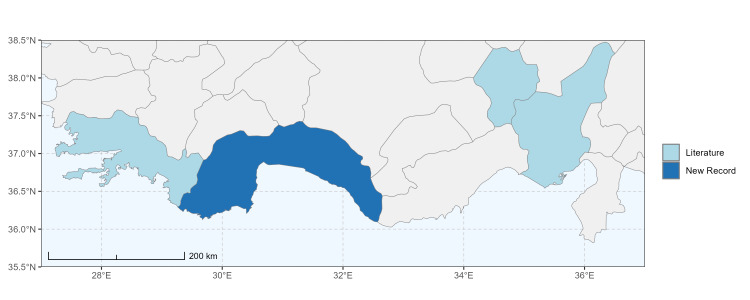
Distribution of *A. taedium* in the Mediterranean Region of Türkiye.

Adana, Adıyaman, Ağrı, Aydın (Çine), Bolu, Kars, Malatya, Muğla, Nevşehir (Göreme), Niğde (Çamardı), Siirt, Şırnak (Wood 2023a); Antalya, Muğla, Niğde (this study) (Fig. 79).

#### Flower records

**Brassicaceae**: *Lepidium chalepense* L., **Fabaceae**: *Securigera libanotica* (Boiss.) Lassen, *Trigonella kotschyi* Fenzl.

### Andrena (Melandrena) thoracica

(Fabricius, 1775)

A248FD76-CD2E-5724-A608-87DA50247F9E

#### Materials

- **Type status:** Other material. **Occurrence:** recordedBy: Fatih Dikmen; individualCount: 2; sex: male; occurrenceID: C1C79A05-6629-5955-9AFF-FCB92B4A821C; **Location:** countryCode: TR; stateProvince: Niğde; locality: Ulukışla road, 3 km before Kolsuz Pass; verbatimElevation: 1305 m; verbatimLatitude: 37.374; verbatimLongitude: 34.833; **Identification:** identifiedBy: Thomas J. Wood; **Event:** eventDate: 22-05-2008; **Record Level:** collectionCode: ZMUI

#### Distribution

Algeria, Austria, Belgium, Bulgaria, China, Corsica, Croatia, Cyprus, Denmark, Estonia, France, Germany, Great Britain, Greece, Hungary, Iran, Italy, Latvia, Lithuania, Luxembourg, Malta, Moldova, Monaco, Morocco, Netherlands, Norway, Poland, Portugal, Romania, Russia, San Marino, Slovakia, Slovenia, Spain, Sweden, Switzerland, Tunisia, Türkiye, Ukraine and Uzbekistan (Gusenleitner and Schwarz 2002, Osytshnjuk et al. 2008, Wood and Monfared 2022, Ascher and Pickering 2025).

##### Distribution in Türkiye

**Figure 80.**
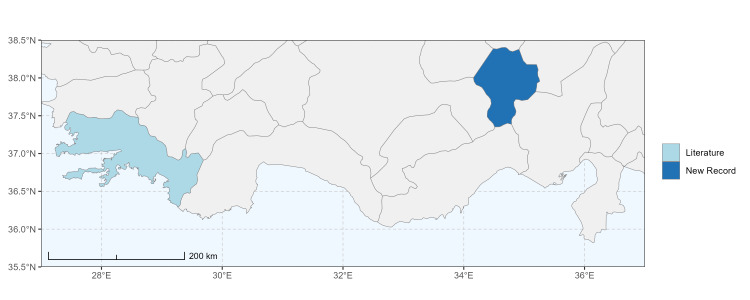
Distribution of *A. thoracica* in the Mediterranean Region of Türkiye.

**Figure 81.**
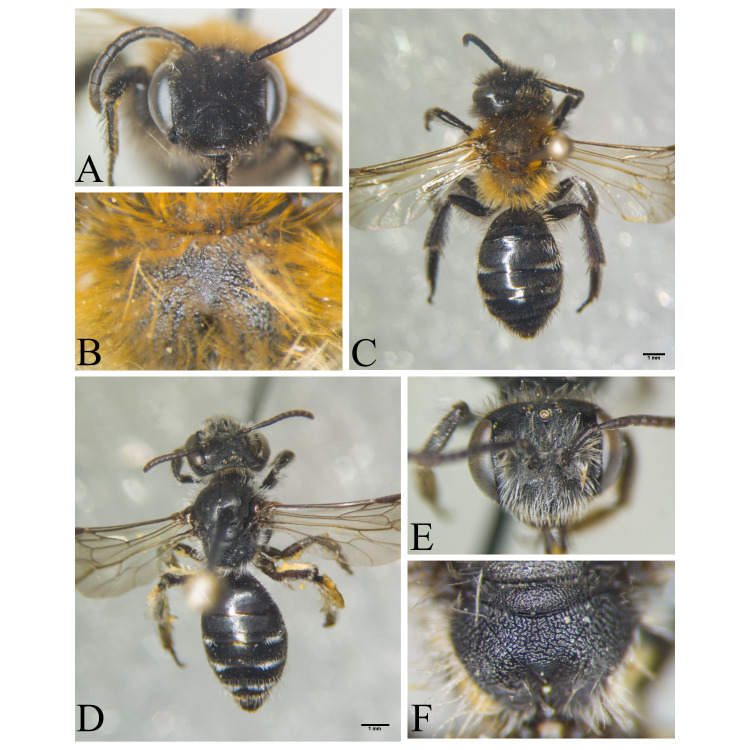
*Andrena thoracica*, male. Head, frontal view (A); propodeum, dorsal view (B); habitus, dorsal view (C). *Andrena torda*, female. Habitus, dorsal view (D), head, frontal view (E), propodeum, dorsal view (F).

Amasya, Balıkesir (Ayvalık), Eskişehir (Sivrihisar), Manisa (Warncke 1966); Van (Işık Mountain) (Warncke 1969); Ankara, Aydın, Erzurum, Muğla (Yatağan), Sivas (Hazır 2010, Hazır et al. 2014); Afyon (Sultandağı) (Güler et al. 2015); Niğde (this study) (Fig. 80 and Fig. 81A-C).

#### Flower records

**Amaryllidaceae**: *Allium* L., *Narcissus* L. **Apiaceae**: *Anthriscus* Pers., *Heracleum sphondylium* L. **Asteraceae**: *Achillea millefolium* L., *Anthemis* L., *Arctium minus* (Hill) Bernh., *Bellis perennis* L., *Centaurea nigra* L., *Cirsium* Mill., *Crepis* L., *Petasites* Mill., *Senecio* L., *Sonchus* L., *Taraxacum* F.H.Wigg., *Tussilago farfara* L. **Brassicaceae**: *Cardamine pratensis* L. **Caryophyllaceae**: *Stellaria* L. **Convolvulaceae**: *Convolvulus arvensis* L. **Cornaceae**: *Cornus sanguinea* L. **Cucurbitaceae**: *Bryonia dioica* Bojer **Fagaceae**: *Castanea sativa* Mill. **Lamiaceae**: *Glechoma hederacea* L., *Lamium album* L. **Lauraceae**: *Laurus nobilis* L. **Malvaceae**: *Tilia* L. **Oleaceae**: *Ligustrum vulgare* L. **Onagraceae**: *Chamaenerion angustifolium* (L.) Scop. **Orchidaceae**: *Herminium* L. **Papaveraceae**: *Papaver* L. **Ranunculaceae**: *Caltha palustris* L., *Clematis vitalba* L., *Ranunculus ficaria* L., *Ranunculus* L. **Rosaceae**: *Filipendula ulmaria* (L.) Maxim., *Potentilla* L., *Prunus* L., *Pyrus* L., *Rubus fruticosus* agg. L., *Rubus* L. **Salicaceae**: *Salix* L., *Salix triandra* L. **Sapindaceae**: *Acer campestre* L., *Acer pseudoplatanus* L., *Acer* L. (Balfour et al. 2022); **Cistaceae**: *Cistus ladanifer* L. (Lanuza et al. 2025); **Brassicaceae**: *Nasturtium officinale* R.Br.

### Andrena (Cordandrena) torda

Warncke, 1965

FCF0E656-34C2-5838-B570-8AF2969DEE30

#### Materials

- **Type status:** Other material. **Occurrence:** recordedBy: Fatih Dikmen; individualCount: 9; sex: female; occurrenceID: A6D7F031-6350-5219-A943-88DD2B64E82E; **Location:** countryCode: TR; stateProvince: Muğla; locality: Datça road; verbatimElevation: 278 m; verbatimLatitude: 36.797; verbatimLongitude: 28.709; **Identification:** identifiedBy: Thomas J. Wood; **Event:** eventDate: 03-04-2009; **Record Level:** collectionCode: ZMUI

#### Distribution

Cyprus, Greece, Iran, Iraq, Israel and West Bank, Lebanon and Türkiye (Gusenleitner and Schwarz 2002, Wood and Monfared 2022, Wood et al. 2024).

##### Distribution in Türkiye

**Figure 82.**
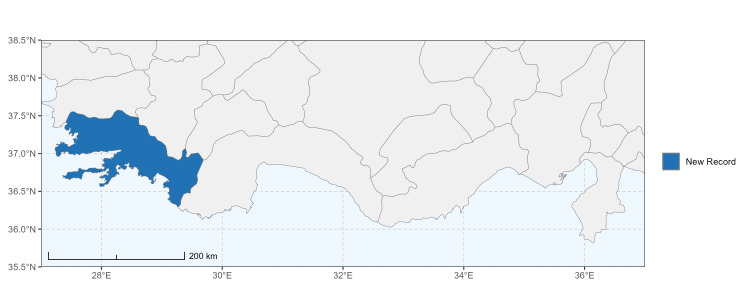
Distribution of *A. torda* in the Mediterranean Region of Türkiye.

Konya (Beyşehir, Sarayönü) (Warncke 1966); Ankara, Aydın, (Beyşehir), Diyarbakır (Warncke 1974); Aydın (Hazır et al. 2014); Muğla (this study) (Fig. 81D-F and Fig. 82).

#### Flower records

**Apiaceae**: *Tordylium apulum* L. **Asteraceae**: *Anthemis chia* L.

### Andrena (Simandrena) transitoria

Morawitz, 1871

E2DF2A79-1EF2-5D51-B8BB-57A7CBA895C9

#### Materials

- **Type status:** Other material. **Occurrence:** recordedBy: Fatih Dikmen; individualCount: 1; sex: female; occurrenceID: 44670737-31F5-533B-9DD0-36C9D43BD9AB; **Location:** countryCode: TR; stateProvince: Kahramanmaraş; locality: East of vineyard houses; verbatimElevation: 1288 m; verbatimLatitude: 37.634; verbatimLongitude: 36.957; **Identification:** identifiedBy: Thomas J. Wood; **Event:** eventDate: 01-07-2009; **Record Level:** collectionCode: ZMUI

#### Distribution

Albania, Armenia, Azerbaijan, Bulgaria, Georgia, Greece, Hungary, Iran, Israel and the West Bank, Italy, Jordan, Lebanon, Moldova, North Macedonia, Romania, Russia, Serbia, Slovakia, Syria, Turkmenistan, Türkiye and Ukraine (Warncke 1974, Gusenleitner and Schwarz 2002, Wood and Monfared 2022, Ascher and Pickering 2025).

##### Distribution in Türkiye

**Figure 83.**
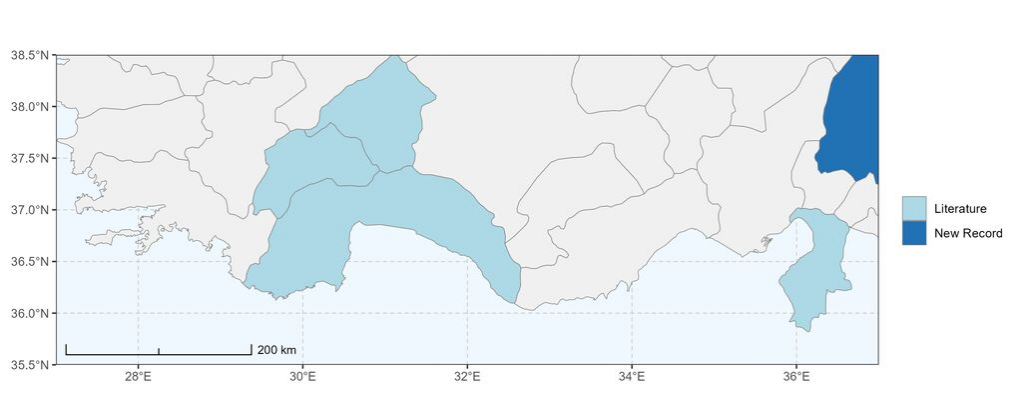
Distribution of *A. transitoria* in the Mediterranean Region of Türkiye.

Ankara, Elazığ, Eskişehir, Hatay, Konya (Beyşehir), Nevşehir (Ürgüp) (Warncke 1966); Erzurum (Tortum), Kars (Kağızman) (Özbek 1976); Aksaray, Ankara, Antalya, Bolu, Burdur, Antep, Isparta, Kırşehir, Konya, Kütahya, Nevşehir (Hazır 2010, Hazır et al. 2014); Kahramanmaraş (this study) (Fig. 83).

#### Flower records

**Asteraceae**: *Centaurea virgata* Lam.

### Andrena (Truncandrena) truncatilabris

Morawitz, 1877

7320035B-2974-59FC-9967-51E3DE96E988

#### Materials

- **Type status:** Other material. **Occurrence:** recordedBy: Fatih Dikmen; individualCount: 4; sex: female; occurrenceID: EE9C4F66-7B76-5B15-A037-C5BA6F64EC3B; **Location:** countryCode: TR; stateProvince: Burdur; locality: Antalya road, Korkuteli turn; verbatimElevation: 783 m; verbatimLatitude: 37.313; verbatimLongitude: 30.49; **Identification:** identifiedBy: Thomas J. Wood; **Event:** eventDate: 29-05-2009; **Record Level:** collectionCode: ZMUI
- **Type status:** Other material. **Occurrence:** recordedBy: Fatih Dikmen; individualCount: 1; sex: female; occurrenceID: 75A2941A-A5FD-56B4-9ECF-BC452D3DC16A; **Location:** countryCode: TR; stateProvince: Mersin; locality: Elmalıkuzu village; verbatimElevation: 1381 m; verbatimLatitude: 36.423; verbatimLongitude: 33.05; **Identification:** identifiedBy: Thomas J. Wood; **Event:** eventDate: 07-06-2009; **Record Level:** collectionCode: ZMUI
- **Type status:** Other material. **Occurrence:** recordedBy: Fatih Dikmen; individualCount: 2; sex: female; occurrenceID: 9D97F152-F711-58E8-AE4E-4CA5A72AD98C; **Location:** countryCode: TR; stateProvince: Antalya; locality: Korkuteli-Finike road; verbatimElevation: 1346 m; verbatimLatitude: 36.949; verbatimLongitude: 30.131; **Identification:** identifiedBy: Thomas J. Wood; **Event:** eventDate: 10-06-2009; **Record Level:** collectionCode: ZMUI

#### Distribution

Albania, Algeria, Armenia, Austria, Azerbaijan, Bosnia and Herzegovina, Bulgaria, Croatia, Cyprus, France, Georgia, Greece, Hungary, Iran, Israel and the West Bank, Italy, Jordan, Lebanon, Montenegro, Morocco, North Macedonia, Portugal, Romania, Russia, Serbia, Slovakia, Slovenia, Spain, Syria, Tunisia, Turkmenistan, Türkiye and Ukraine (Gusenleitner and Schwarz 2002, Wood and Monfared 2022, Ascher and Pickering 2025).

##### Distribution in Türkiye

**Figure 84.**
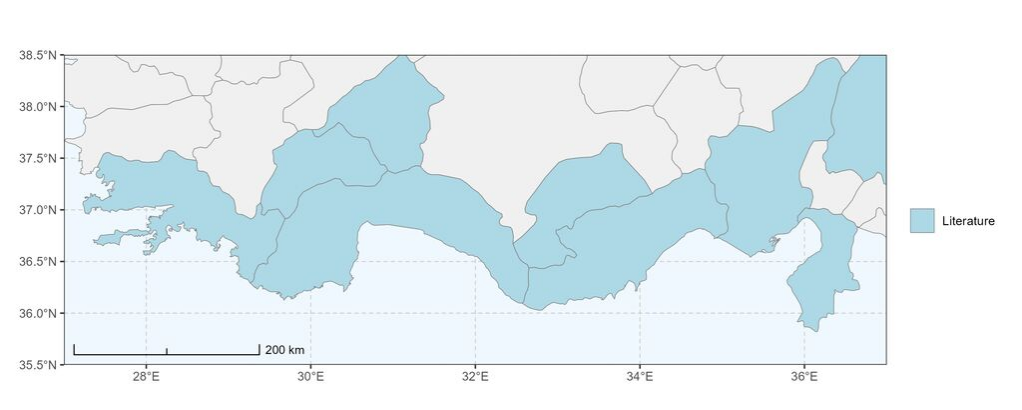
Distribution of *A. truncatilabris* in the Mediterranean Region of Türkiye.

Adana, Amanos Mountains, Ankara, Balıkesir (Ayvalık), Konya, Mersin (Tarsus), Sakarya (Adapazarı) (Warncke 1966); Erzurum (Tortum, Oltu, Narman, İspir, Horasan), Kars, Muş (Özbek 1976); Adana, Aksaray, Amasya, Ankara, Antalya, Antep, Aydın, Balıkesir, Burdur, Çanakkale, Çorum, Hatay, Isparta, Maraş, Karaman, Kırşehir, Konya, Kütahya, Mersin, Muğla, Nevşehir, Sivas, Yozgat (Hazır 2010, Hazır et al. 2014); Antalya, Burdur, Mersin (this study) (Fig. 84).

#### Flower records

**Brassicaceae**: Hirschfeldia incana (L.) Lagr.-Foss., *Diplotaxis tenuifolia* (L.) DC. **Fabaceae**: *Medicago* L. **Geraniaceae**: Erodium cicutarium subsp. cicutarium (L.) L Hér.

### Andrena (Truncandrena) tscheki tritica

Warncke, 1965

49D20A49-F3E4-5FC2-82E5-D02644E8084C

#### Materials

- **Type status:** Other material. **Occurrence:** recordedBy: Fatih Dikmen; individualCount: 1; sex: female; occurrenceID: C9FC2BB7-7585-5E7F-8E3A-898CAB1EC9A7; **Location:** countryCode: TR; stateProvince: Hatay; county: Yayladağ; locality: Harbiye; verbatimElevation: 471 m; verbatimLatitude: 36.123; verbatimLongitude: 36.171; **Identification:** identifiedBy: Thomas J. Wood; **Event:** eventDate: 22-03-2009; **Record Level:** collectionCode: ZMUI

#### Distribution

Greece, Israel and the West Bank and Türkiye (Gusenleitner and Schwarz 2002).

##### Distribution in Türkiye

**Figure 85.**
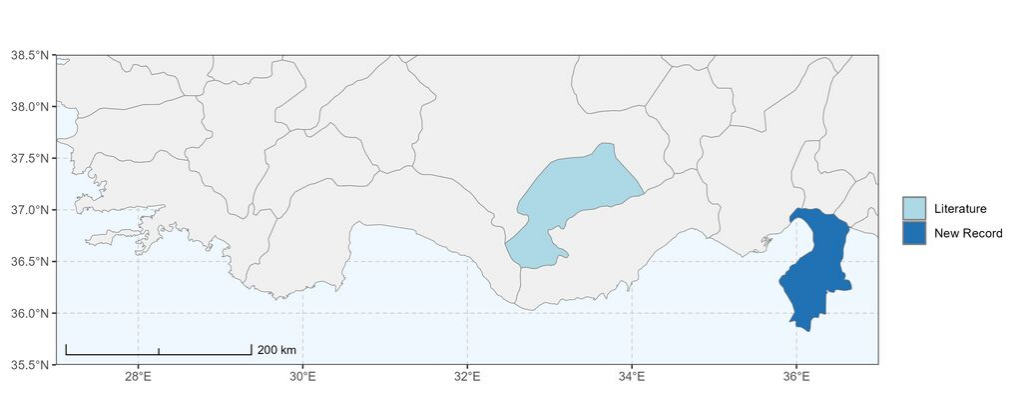
Distribution of A. tscheki ssp. tritica in the Mediterranean Region of Türkiye.

Bolkar Mountains (Warncke 1966); Karaman (Göktepe), Uşak (Elma Mountains) (Warncke 1974); Aydın (Hazır et al. 2014); Hatay (this study) (Fig. 85).

#### Flower records

**Lamiaceae**: Ballota saxatilis subsp. brachyodonta (Boiss.) P.H.Davis & Doroszenko.

### Andrena (Holandrena) variabilis

Smith, 1853

DFEB0ABE-9A54-501D-B81C-2CDF2079859A

#### Materials

- **Type status:** Other material. **Occurrence:** recordedBy: Fatih Dikmen; individualCount: 1; sex: female; occurrenceID: 2DAE3764-A642-584E-BFEA-C2C3B0567F24; **Location:** countryCode: TR; stateProvince: Adana; county: Tufanbeyli; locality: 5 km from Pınarlar village; verbatimElevation: 1451 m; verbatimLatitude: 36.245; verbatimLongitude: 36.289; **Identification:** identifiedBy: Thomas J. Wood; **Event:** eventDate: 02-07-2009; **Record Level:** collectionCode: ZMUI
- **Type status:** Other material. **Occurrence:** recordedBy: Fatih Dikmen; individualCount: 2; sex: female; occurrenceID: 03CEA0D2-BEB7-54E5-A1E3-F5A604888A35; **Location:** countryCode: TR; stateProvince: Mersin; locality: Mut-Gülnar road; verbatimElevation: 993 m; verbatimLatitude: 36.423; verbatimLongitude: 33.488; **Identification:** identifiedBy: Thomas J. Wood; **Event:** eventDate: 22-07-2009; **Record Level:** collectionCode: ZMUI

#### Distribution

Albania, Algeria, Armenia, Austria, Azerbaijan, Bosnia and Herzegovina, Bulgaria, Croatia, Czechia, France, Georgia, Germany, Greece, Hungary, Iran, Israel and the West Bank, Italy, Jordan, Lebanon, Moldova, Montenegro, Morocco, North Macedonia, Portugal, Romania, Serbia, Slovakia, Slovenia, Spain, Switzerland, Syria, Turkmenistan, Türkiye and Ukraine (Gusenleitner and Schwarz 2002, Osytshnjuk et al. 2008, Wood and Monfared 2022, Ascher and Pickering 2025).

##### Distribution in Türkiye

**Figure 86.**
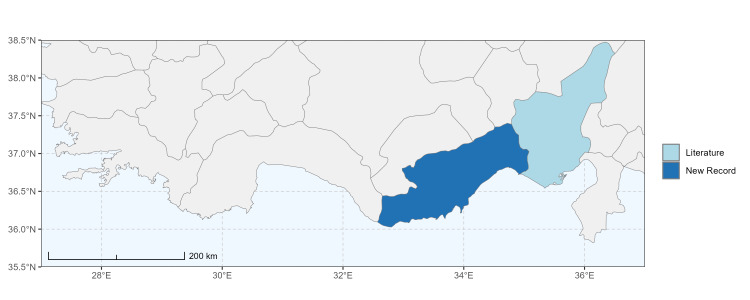
Distribution of *A. variabilis* in the Mediterranean Region of Türkiye.

Amanos Mountains, İstanbul (Warncke 1966); İzmir (Ensetepe) (Warncke 1969); Erzurum (İspir), Iğdır (Tuzluca) (Özbek 1976); Adana, Ankara, Konya, Sivas (Hazır 2010, Hazır et al. 2014); Adana, Mersin (this study) (Fig. 86).

#### Flower records

**Asteraceae**: *Cirsium arvense* (L.) Scop. **Fabaceae**: *Genista acanthoclada* DC. **Lamiaceae**: *Thymbra capitata* (L.) Cav. (Lanuza et al. 2025); **Apiaceae**: Eryngium campestre var. virens Link **Asteraceae**: Echinops pungens var. pungens Trautv. **Boraginaceae**: *Echium italicum* L.

### Andrena (Simandrena) vetula

Lepeletier, 1841

C079C0B4-892B-58D0-9377-9673B5F83576

#### Materials

- **Type status:** Other material. **Occurrence:** recordedBy: Fatih Dikmen; individualCount: 2; sex: female; occurrenceID: 28B1C3E9-D074-551A-B46A-C6D8C442FD37; **Location:** countryCode: TR; stateProvince: Mersin; county: Mut; locality: Sarıkavak road; verbatimElevation: 665 m; verbatimLatitude: 36.565; verbatimLongitude: 33.63; **Identification:** identifiedBy: Thomas J. Wood; **Event:** eventDate: 25-04-2008; **Record Level:** collectionCode: ZMUI

#### Distribution

Algeria, Azerbaijan, Croatia, Cyprus, Egypt, France, Georgia, Greece, Iran, Israel and the West Bank, Italy, Jordan, Lebanon, Libya, Morocco, Portugal, Russia, Spain, Syria, Tajikistan, Tunisia, Turkmenistan, Türkiye, Uzbekistan and Yemen (Gusenleitner and Schwarz 2002, Wood and Monfared 2022, Wood et al. 2024, Ascher and Pickering 2025).

##### Distribution in Türkiye

**Figure 87.**
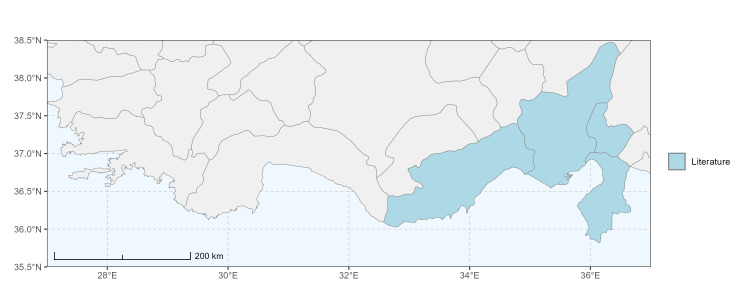
Distribution of *A. vetula* in the Mediterranean Region of Türkiye.

Adana (Karataş), Mersin (Mut, Silifke, Tarsus) (Warncke 1966); Amasya, Ankara (Warncke 1974); Erzurum (Narman, Oltu, Tortum) (Özbek 1976); Adana, Ankara, Antep, Çorum, Hatay, Mersin, Osmaniye, Urfa (Hazır 2010, Hazır et al. 2014); Mersin (this study) (Fig. 87).

#### Flower records

**Brassicaceae**: *Diplotaxis tenuifolia* (L.) DC.

### Andrena (Ulandrena) westensis

Warncke, 1965

9D11F51B-D91A-576A-88BF-C90BCA5D1FE5

#### Materials

- **Type status:** Other material. **Occurrence:** recordedBy: Fatih Dikmen; individualCount: 1; sex: female; occurrenceID: C95E5840-4E46-531B-BAAE-249CEFF142A8; **Location:** countryCode: TR; stateProvince: Muğla; county: Marmaris; locality: Özal Bay; verbatimElevation: 0 m; verbatimLatitude: 36.924; verbatimLongitude: 28.177; **Identification:** identifiedBy: Thomas J. Wood; **Event:** eventDate: 02-04-2009; **Record Level:** collectionCode: ZMUI
- **Type status:** Other material. **Occurrence:** recordedBy: Fatih Dikmen; individualCount: 1; sex: female; occurrenceID: 8E0234F8-27AA-545B-B95F-C6B57758EB06; **Location:** countryCode: TR; stateProvince: Muğla; county: Marmaris; locality: Bördübet road; verbatimElevation: 195 m; verbatimLatitude: 36.857; verbatimLongitude: 28.126; **Identification:** identifiedBy: Thomas J. Wood; **Event:** eventDate: 04-04-2009; **Record Level:** collectionCode: ZMUI

#### Distribution

Greece and Türkiye (Ascher and Pickering 2025).

##### Distribution in Türkiye

**Figure 88.**
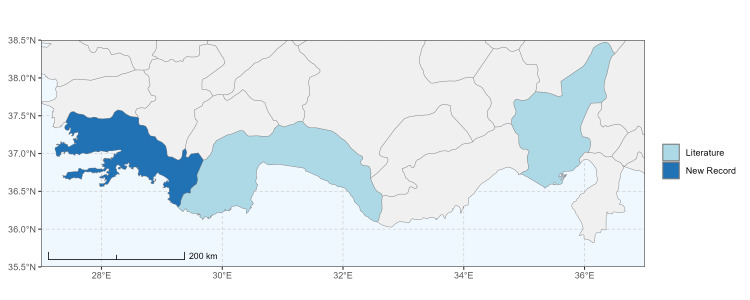
Distribution of *A. westensis* in the Mediterranean Region of Türkiye.

Adana (Gülek), Ankara, Antalya (Anamur, Finike), Çanakkale (Bigadiç), Manisa (Warncke 1974); Antalya, Aydın (Hazır 2010, Hazır et al. 2014); Muğla (this study) (Fig. 88).

#### Flower records

**Asteraceae**: *Crepis commutata* (Spreng.) Greuter, *Hypochaeris achyrophorus* L., *Leontodon tuberosus* L. **Cistaceae**: *Cistus salviifolius* L. (Lanuza et al. 2025); **Asteraceae**: *Crepis sancta* (L.) Bornm., *Crepis* L.

### Andrena wolfi

Gusenleitner & Scheuchl, 2000

D4F3C2F8-F18B-51A4-9283-CD7619238C51

#### Materials

- **Type status:** Other material. **Occurrence:** recordedBy: Fatih Dikmen; individualCount: 4; sex: male; occurrenceID: 25D7A3B0-E7B2-5948-BB32-B06CB74F6AEE; **Location:** countryCode: TR; stateProvince: Hatay; locality: between Reyhanlı and Antakya; verbatimElevation: 85 m; verbatimLatitude: 36.252; verbatimLongitude: 36.519; **Identification:** identifiedBy: Thomas J. Wood; **Event:** eventDate: 21-03-2009; **Record Level:** collectionCode: ZMUI

#### Distribution

Israel and the West Bank, Jordan, Syria and Türkiye* (Gusenleitner and Schwarz 2002, Ghisbain et al. 2023).

##### Distribution in Türkiye

**Figure 89.**
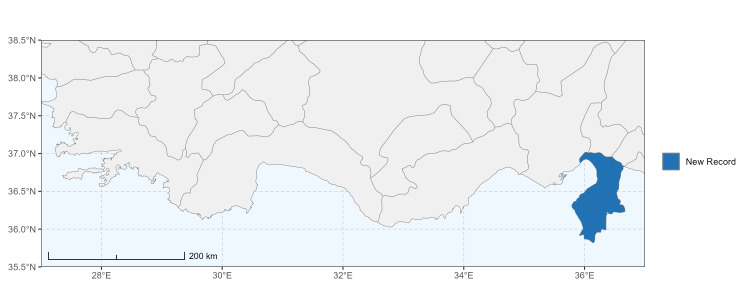
Distribution of *A. wolfi* in the Mediterranean Region of Türkiye.

**Figure 90.**
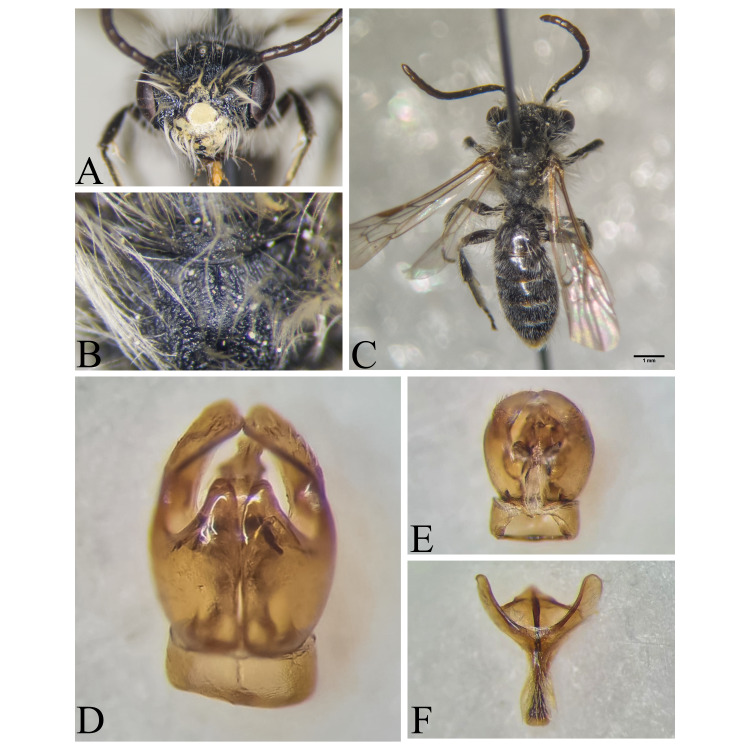
*Andrena wolfi*, male. Head, frontal view (A); propodeum, dorsal view (B); habitus, dorsal view (C); genital capsule, dorsal view (D); genital capsule, ventral view (E); S7 and S8 (F).

*Hatay (new record for Türkiye) (Fig. 89 and Fig. 90).

#### Flower records

**Brassicaceae**: *Erucaria microcarpa* Boiss. **Caryophyllaceae**: *Silene aegyptiaca* (L.) L.f. (Pisanty et al. 2016); **Brassicaceae**: *Ochthodium aegyptiacum* (L.) DC.

## Analysis

The table below (Table 1) summarises 81 species of *Andrena* bees collected from the Mediterranean Region of Türkiye. It provides taxonomic classification, subgenus information and authorship details. This compilation offers valuable data on the diversity and distribution of *Andrena* species in this biologically rich area.

**Biogeographical distribution**

This study documented a total of 26 subgenera and 81 *Andrena* taxa in the Mediterranean Region of Türkiye. Amongst these taxa, *Andrena wolfi* was identified as a new record for the Turkish fauna. Subgeneric richness (Table 1) was highest in *Micrandrena* (12 species) and *Melandrena* (8 species), indicating that these groups contribute substantially to the regional *Andrena* diversity. Provincial distribution analyses revealed that *A. alfkenelloides*, *A. flavipes*, *A. fulvitarsis*, *A. labialis*, *A. minutula* and *A. truncatilabris* were the most widespread species across the Mediterranean provinces. The complete provincial distribution of all 81 species is presented in Table 2, while the number of newly-recorded species by province is detailed in Table 3.

## Discussion

Situated at the confluence of three major> biogeographical realms (Mediterranean, Euro-Siberian and Irano-Turanian), Türkiye supports an exceptionally rich apifauna. However, despite this significant species diversity, knowledge regarding the distribution and systematics of the local *Andrena* fauna remains fragmented and comprehensive faunistic inventories are notably lacking (Güler 2011, Hazır et al. 2014, Güler et al. 2014, Dikmen 2018, Wood 2023a, Wood 2023b). In this context, the present study provides a significant contribution to filling this gap by documenting 81 *Andrena* taxa in the Mediterranean Region, a critical biodiversity hotspot. Amongst the sampled locations, species were observed to be most concentrated in the provinces of Hatay, Muğla and Antalya (Table 3). Our analyses of provincial distribution patterns identified *A. alfkenelloides*, *A. flavipes*, *A. fulvitarsis*, *A. labialis*, *A. minutula* and *A. truncatilabris* as the most widespread taxa across the Mediterranean Region. This faunistic composition is highly consistent with previous large-scale studies of Turkish *Andrena* fauna (e.g. Hazır (2010), Hazır et al. (2014)), particularly with regard to the dominance of *A. truncatilabris*, *A. labialis* and *A. flavipes*.

The ubiquity of these species is likely due to their broad ecological plasticity and life-history traits. For instance, *A. truncatilabris*, reported by Hazır (2010) as the most widespread species in Türkiye with a preference for steppe habitats, clearly maintains a strong presence in the Mediterranean zone, indicating a high tolerance for varying climatic conditions. Similarly, the extensive distribution of *A. flavipes* is well supported by literature: Warncke (1974) described it as ubiquitous and Özbek (1975, 1978) linked its success to its bivoltine phenology (two generations per year) and polylectic foraging behaviour (Hazır 2010). These traits enable *A. flavipes* to exploit a diverse array of floral resources over a prolonged flight period, facilitating its colonisation of various habitats, including agricultural landscapes.

An analysis of the recorded faunal composition reveals a relatively low rate of endemism; only three of the 81 taxa (Andrena hungarica ssp. macroura, *A. purpureomicans* and *A. cinnamonea*) are known endemics (approx. 3.7%). Regional distribution analysis further supports this pattern. Approximately 9% (7 taxa) of the studied fauna are restricted to the Mediterranean Region within the scope of these data. However, the vast majority (91%) are also recorded in other geographical regions of Turkey. This low level of endemicity suggests that the regional fauna is not highly isolated, but is rather dominated by species with wider geographical ranges; this contrasts with the pattern seen on the main Anatolian Plateau and in mountains areas (Warncke 1974).

Amongst the endemic taxa, Andrena hungarica ssp. macroura is of particular interest due to its complex taxonomy and distribution. Recent assessments show that the nominate form, *Andrena hungarica* Friese, 1887, is restricted to the Pannonian Basin. Furthermore, A. h. ssp. khursiensis Osytshnjuk, 1994 is now considered a junior synonym of *A. quadimaculata* Friese, 1921 (Wood and Bossert 2025). As a result, A. h. ssp. macroura remains the only valid lineage of this group, endemic to Anatolia. Previously, literature described this subspecies as a steppe element found only in the high-altitude regions of Central and Eastern Anatolia (Warncke 1974). Therefore, its discovery in a coastal Mediterranean locality (Muğla) is surprising, as this habitat differs significantly from its typical range. Despite this difference in climate, the morphology of our specimens matches the diagnostic description of the subspecies perfectly, confirming the identification (Fig. 37D-F). We hypothesise that this isolated coastal population may be a glacial relict that remained after populations moved to lower altitudes during the cooler periods of the Pleistocene. Alternatively, this finding suggests that Andrena hungarica ssp. macroura has higher ecological plasticity than previously thought. Future studies using molecular data and ecological modelling are needed to determine if this population is a separate cryptic lineage or simply shows the species' ability to adapt.

The most notable faunistic outcome of this study is the first record of *Andrena wolfi* from Türkiye. The identification is unequivocally supported by the morphology of the collected male specimens; specifically, the structure of the genital capsule and the hidden sterna (S7 and S8) exhibit complete congruence with the diagnostic characters described and illustrated by Pisanty et al. (2016) (Fig. 90). Historically, the known distribution of *A. wolfi* has been concentrated in the Levant (Gusenleitner and Schwarz 2002, Ghisbain et al. 2023). Consequently, the discovery of *Andrena wolfi* in southern Türkiye suggests two plausible scenarios: (1) the species is undergoing a natural northward range expansion, potentially driven by current climatic dynamics or (2) it was previously overlooked due to insufficient sampling intensity in suitable habitats during earlier faunistic surveys.

Beyond the distributional patterns, ecological data obtained from field surveys elucidate the critical floral associations of the regional *Andrena* community. Our results exhibit a strong concordance with literature records regarding the dominance of key plant families; however, slight variations in preference hierarchies were observed. While aggregated literature data generally position Asteraceae (hosting 25 species) as the primary pollen source, followed by Brassicaceae (22 species) and Fabaceae (16 species), our field observations revealed a slight dominance of Brassicaceae, which supported the highest diversity of *Andrena* taxa (38 species), followed closely by Asteraceae (35 species). Whereas published records most frequently identify *Crepis* (14 species), *Brassica* (11 species) and *Senecio* (10 species) as key foraging genera for *Andrena*, our field observations reveal a different pattern of floral use. In our dataset, the genera *Crepis* (18 species), *Sisymbrium* (9 species) and *Hirschfeldia* (8 species) were recorded as the most frequently visited. Notably, *Crepis* stands out as a consistently important floral resource, ranking as the top genus in both literature-based records and our field data. Additionally, the dietary classifications and specific floral specialisations of the documented *Andrena* species are summarised in Table 4.

## Supplementary Material

XML Treatment for Andrena (Ulandrena) abbreviata

XML Treatment for Andrena (Notandrena) aerinifrons

XML Treatment for Andrena (Micrandrena) alfkenella

XML Treatment for Andrena (Micrandrena) alfkenelloides

XML Treatment for Andrena (Micrandrena) alutacea

XML Treatment for Andrena (Chlorandrena) astica

XML Treatment for Andrena (Euandrena) bicolor

XML Treatment for Andrena (Plastandrena) bimaculata

XML Treatment for Andrena (Aenandrena) bisulcata

XML Treatment for Andrena (Cryptandrena) brumanensis

XML Treatment for Andrena (Micrandrena) cedricola

XML Treatment for Andrena (Chlorandrena) cinereophila

XML Treatment for Andrena (Simandrena) cinnamonea

XML Treatment for Andrena (Brachyandrena) colletiformis

XML Treatment for Andrena (Truncandrena) combusta

XML Treatment for Andrena (Cordandrena) cordialis

XML Treatment for Andrena (Poecilandrena) crassana

XML Treatment for Andrena (Cordandrena) cypria

XML Treatment for Andrena discordia

XML Treatment for Andrena (Simandrena) dorsata

XML Treatment for Andrena (Micrandrena) enslinella

XML Treatment for Andrena (Campylogaster) erberi

XML Treatment for Andrena (Chlorandrena) exquisita

XML Treatment for Andrena (Leimelissa) fallax

XML Treatment for Andrena (Holandrena) flavilabris

XML Treatment for Andrena (Melandrena) flavipes

XML Treatment for Andrena (Micrandrena) floricola

XML Treatment for Andrena (Holandrena) forsterella

XML Treatment for Andrena (Chrysandrena) fulvago

XML Treatment for Andrena (Ulandrena) fulvitarsis

XML Treatment for Andrena (Melanapis) fuscosa

XML Treatment for Andrena (Simandrena) gasparella

XML Treatment for Andrena (Chrysandrena) glandaria

XML Treatment for Andrena (Chrysandrena) hesperia

XML Treatment for Andrena (Chlorandrena) humabilis

XML Treatment for Andrena (Melandrena) hungarica macroura

XML Treatment for Andrena (Fuscandrena) iliaca

XML Treatment for Andrena (Micrandrena) immaculata

XML Treatment for Andrena (Ulandrena) isabellina

XML Treatment for Andrena (Ulandrena) kriechbaumeri

XML Treatment for Andrena (Holandrena) labialis

XML Treatment for Andrena (Aciandrena) lamiana

XML Treatment for Andrena (Poecilandrena) laticeps

XML Treatment for Andrena (Simandrena) lepida

XML Treatment for Andrena (Melandrena) limata

XML Treatment for Andrena (Melandrena) magna

XML Treatment for Andrena (Micrandrena) minutula

XML Treatment for Andrena monacha

XML Treatment for Andrena (Hamandrena) nasuta

XML Treatment for Andrena (Ulandrena) neocypriaca

XML Treatment for Andrena (Melandrena) nigroaenea

XML Treatment for Andrena (Melandrena) nitidemula

XML Treatment for Andrena (Nobandrena) nobilis

XML Treatment for Andrena (Micrandrena) oedicnema

XML Treatment for Andrena (Truncandrena) optata

XML Treatment for Andrena (Chlorandrena) orientana

XML Treatment for Andrena (Chlorandrena) panurgimorpha

XML Treatment for Andrena (Euandrena) pileata canigica

XML Treatment for Andrena (Plastandrena) pilipes

XML Treatment for Andrena (Notandrena) purpureomicans

XML Treatment for Andrena (Melandrena) pyropygia

XML Treatment for Andrena (Truncandrena) rufomaculata

XML Treatment for Andrena (Euandrena) rufula

XML Treatment for Andrena (Micrandrena) rugothorace

XML Treatment for Andrena (Micrandrena) rugulosa

XML Treatment for Andrena (Taeniandrena) russula

XML Treatment for Andrena (Opandrena) schencki

XML Treatment for Andrena (Truncandrena) schmiedeknechti

XML Treatment for Andrena (Scitandrena) scita

XML Treatment for Andrena (Micrandrena) spreta

XML Treatment for Andrena (Euandrena) symphyti

XML Treatment for Andrena (Taeniandrena) taedium

XML Treatment for Andrena (Melandrena) thoracica

XML Treatment for Andrena (Cordandrena) torda

XML Treatment for Andrena (Simandrena) transitoria

XML Treatment for Andrena (Truncandrena) truncatilabris

XML Treatment for Andrena (Truncandrena) tscheki tritica

XML Treatment for Andrena (Holandrena) variabilis

XML Treatment for Andrena (Simandrena) vetula

XML Treatment for Andrena (Ulandrena) westensis

XML Treatment for Andrena wolfi

## Figures and Tables

**Table 1.** Species List of *Andrena* Bees from the Turkish Mediterranean Area.

**Subgenus**	**Taxon priority**	**Author**
* Aciandrena *	*Andrena lamiana*	Warncke, 1965
* Aenandrena *	*Andrena bisulcata*	Morawitz, 1877
* Brachyandrena *	*Andrena colletiformis*	Morawitz, 1873
* Campylogaster *	*Andrena erberi*	Morawitz, 1871
* Chlorandrena *	*Andrena astica*	Warncke, 1967
	*Andrena cinereophila*	Warncke, 1965
	*Andrena exquisita*	Warncke, 1975
	*Andrena humabilis*	Warncke, 1965
	*Andrena orientana*	Warncke, 1965
	*Andrena panurgimorpha*	Mavromoustakis, 1957
* Chrysandrena *	*Andrena fulvago*	(Christ, 1791)
	*Andrena glandaria*	Warncke, 1975
	*Andrena hesperia*	Smith, 1853
* Cordandrena *	*Andrena cordialis*	Morawitz, 1877
	*Andrena cypria*	Pittioni, 1950
	*Andrena torda*	Warncke, 1965
* Cryptandrena *	*Andrena brumanensis*	Friese, 1899
* Euandrena *	*Andrena bicolor*	Fabricius, 1775
	Andrena pileata ssp. canigica	Warncke, 1975
	*Andrena rufula*	Schmiedeknecht, 1883
	*Andrena symphyti*	Schmiedeknecht, 1883
* Fuscandrena *	*Andrena iliaca*	Warncke, 1969
* Hamandrena *	*Andrena nasuta*	Giraud, 1863
* Holandrena *	*Andrena flavilabris*	Schenck, 1874
	*Andrena forsterella*	Osytshnjuk, 1978
	*Andrena labialis*	(Kirby, 1802)
	*Andrena variabilis*	Smith, 1853
* Leimelissa *	*Andrena fallax*	Eversmann, 1852
* Melanapis *	*Andrena fuscosa*	Erichson, 1835
* Melandrena *	*Andrena flavipes*	Panzer, 1799
	Andrena hungarica ssp. macroura*	Warncke, 1975
	*Andrena limata*	Smith, 1853
	*Andrena magna*	Warncke, 1965
	*Andrena nigroaenea*	(Kirby, 1802)
	*Andrena nitidemula*	Scheuchl & Hazır, 2012
	*Andrena pyropygia*	Kreichbaumer, 1873
	*Andrena thoracica*	(Fabricius, 1775)
* Micrandrena *	*Andrena alfkenella*	Perkins, 1914
	*Andrena alfkenelloides*	Warncke, 1965
	*Andrena alutacea*	Stöckhert, 1942
	*Andrena cedricola*	Wood, 2020
	*Andrena enslinella*	Stöckhert, 1924
	*Andrena floricola*	Eversmann, 1852
	*Andrena immaculata*	Warncke, 1975
	*Andrena minutula*	(Kirby, 1802)
	*Andrena oedicnema*	Warncke, 1975
	*Andrena rugothorace*	Warncke, 1965
	*Andrena rugulosa*	Stöckhert, 1935
	*Andrena spreta*	Pérez, 1895
* Nobandrena *	*Andrena nobilis*	Morawitz, 1873
* Notandrena *	*Andrena aerinifrons*	Dours, 1873
	Andrena purpureomicans*	Alfken, 1935
* Opandrena *	*Andrena schencki*	Morawitz, 1866
* Plastandrena *	*Andrena bimaculata*	(Kirby, 1802)
	*Andrena pilipes*	Fabricius, 1781
* Poecilandrena *	*Andrena crassana*	Warncke, 1965
	*Andrena laticeps*	Morawitz, 1877
* Scitandrena *	*Andrena scita*	Eversmann, 1852
* Simandrena *	Andrena cinnamonea*	Warncke, 1975
	*Andrena dorsata*	(Kirby, 1802)
	*Andrena gasparella*	Patiny, 1998
	*Andrena lepida*	Schenck, 1861
	*Andrena transitoria*	Morawitz, 1871
	*Andrena vetula*	Lepeletier, 1841
* Taeniandrena *	*Andrena russula*	Lepeletier, 1841
	*Andrena taedium*	Wood, 2023
* Truncandrena *	*Andrena combusta*	Morawitz, 1876
	*Andrena optata*	Warncke, 1975
	*Andrena rufomaculata*	Friese, 1921
	*Andrena schmiedeknechti*	Magretti, 1883
	*Andrena truncatilabris*	Morawitz, 1877
	Andrena tscheki ssp. tritica	Warncke, 1965
* Ulandrena *	*Andrena abbreviata*	Dours, 1873
	*Andrena fulvitarsis*	Brullé, 1833
	*Andrena isabellina*	Warncke, 1969
	*Andrena kriechbaumeri*	Schmiedeknecht, 1883
	*Andrena neocypriaca*	Mavromoustakis, 1956
	*Andrena westensis*	Warncke, 1965
*incertae sedis*	*Andrena discordia*	Wood, 2023
	*Andrena monacha*	Warncke, 1965
	Andrena wolfi**	Gusenleitner & Scheuchl, 2000

*: endemic to Türkiye (*Andrena hungarica macroura* Warncke, 1975 subspecies is endemic for Türkiye).

**: new record for Türkiye.

**Table 2.** Distribution of *Andrena* (Hymenoptera, Andrenidae) species in the provinces of the Turkish Mediterranean Region, with records compiled from literature and new collections.

***Andrena* species**	**Literature Records**	**Collection Records**
*Andrena abbreviata*	Adana, Antalya, Denizli, Mersin	Antalya, Mersin
*Andrena aerinifrons*		Hatay*
*Andrena alfkenella*	Adana	Hatay*
*Andrena alfkenelloides*	Adana, Antalya, Burdur, Denizli, Hatay, Mersin, Niğde, Osmaniye	Osmaniye
*Andrena alutacea*	Mersin	Mersin
*Andrena astica*	Adana, Kahramanmaraş	Adana, Hatay*
*Andrena bicolor*	Antalya	Hatay*
*Andrena bimaculata*	Adana	Hatay*
*Andrena bisulcata*	Adana, Hatay, Karaman, Mersin	Mersin
*Andrena brumanensis*	Adana, Antalya, Mersin	Antalya, Muğla*
*Andrena cedricola*		Niğde*
*Andrena cinereophila*	Antalya, Kahramanmaraş, Mersin	Adana*, Hatay*, Mersin, Muğla*
*Andrena cinnamonea*	Antalya, Hatay, Mersin	Hatay
*Andrena colletiformis*	Adana, Antalya, Burdur, Denizli, Karaman	Mersin*
*Andrena combusta*	Mersin	Mersin
*Andrena cordialis*	Burdur, Hatay	Burdur
*Andrena crassana*	Antalya, Hatay, Mersin, Muğla	Burdur*
*Andrena cypria*	Burdur, Mersin, Niğde	Muğla*
*Andrena discordia*	Mersin	Mersin
*Andrena dorsata*	Adana, Antalya, Burdur, Denizli, Hatay, Isparta, Kahramanmaraş, Mersin	Niğde*
*Andrena enslinella*	Antalya, Isparta	Niğde*
*Andrena erberi*	Adana	Mersin*
*Andrena exquisita*	Kahramanmaraş	Hatay*
*Andrena fallax*	Antalya, Burdur, Mersin, Niğde	Antalya
*Andrena flavilabris*		Burdur*
*Andrena flavipes*	Adana, Antalya, Burdur, Denizli, Hatay, Kahramanmaraş, Karaman, Mersin, Muğla	Antalya, Hatay, Kahramanmaraş, Mersin, Niğde*
*Andrena floricola*	Adana, Denizli, Mersin	Mersin
*Andrena forsterella*	Adana, Antalya, Hatay, Mersin	Adana, Kahramanmaraş*, Muğla*
*Andrena fulvago*		Kahramanmaraş*
*Andrena fulvitarsis*	Adana, Antalya, Karaman, Mersin, Muğla, Niğde	Burdur*
*Andrena fuscosa*	Adana, Antalya	Adana, Antalya
*Andrena gasparella*		Antalya*
*Andrena glandaria*	Antalya, Muğla	Adana*, Hatay*, Mersin*, Muğla
*Andrena hesperia*	Adana, Antalya, Burdur, Kahramanmaraş, Mersin, Muğla	Adana, Hatay*
*Andrena humabilis*	Adana, Antalya, Kahramanmaraş, Mersin	Adana, Hatay*, Muğla*, Osmaniye*
*Andrena hungarica*		Muğla*
*Andrena iliaca*		Hatay*
*Andrena immaculata*		Antalya*
*Andrena isabellina*	Hatay	Hatay
*Andrena kriechbaumeri*	Antalya, Mersin, Muğla	Mersin, Muğla
*Andrena labialis*	Antalya, Burdur, Denizli, Karaman, Mersin, Niğde	Adana*, Mersin
*Andrena lamiana*	Adana, Hatay, Mersin	Muğla*
*Andrena laticeps*	Mersin, Niğde	Niğde
*Andrena lepida*	Adana, Antalya, Burdur, Kahramanmaraş, Karaman	Hatay*
*Andrena limata*	Adana, Antalya, Burdur, Denizli, Muğla	Antalya
*Andrena magna*	Niğde	Denizli*
*Andrena minutula*		Antalya*, Muğla*, Osmaniye*
*Andrena monacha*	Adana, Antalya, Muğla, Osmaniye	Mersin*
*Andrena nasuta*	Karaman, Niğde	Mersin*
*Andrena neocypriaca*	Antalya, Hatay, Kahramanmaraş, Mersin, Muğla	Mersin
*Andrena nigroaenea*	Adana, Antalya, Muğla	Antalya
*Andrena nitidemula*	Burdur, Mersin	Mersin, Niğde*
*Andrena nobilis*	Adana, Burdur, Karaman, Mersin	Antalya*, Mersin
*Andrena oedicnema*	Antalya	Antalya, Mersin*
*Andrena optata*	Antalya, Burdur	Antalya
*Andrena orientana*	Adana, Antalya, Denizli, Hatay, Mersin	Muğla*
*Andrena panurgimorpha*	Antalya, Kahramanmaraş, Mersin, Niğde	Adana*
Andrena pileata ssp. canigica		Antalya*
*Andrena pilipes*	Adana, Antalya, Denizli, Mersin, Niğde	Antalya
*Andrena purpureomicans*	Adana, Burdur, Hatay, Niğde	Antalya*
*Andrena pyropygia*	Mersin	Hatay*, Karaman*
*Andrena rufomaculata*	Hatay	Hatay
*Andrena rufula*	Antalya	Hatay*
*Andrena rugothorace*	Burdur, Karaman, Niğde	Adana*, Antalya*, Muğla*
*Andrena rugulosa*		Niğde*
*Andrena russula*	Antalya, Mersin	Muğla*
*Andrena schencki*	Antalya, Mersin	Muğla*
*Andrena schmiedeknechti*	Antalya, Karaman, Mersin, Niğde	Mersin
*Andrena scita*	Antalya, Burdur, Denizli, Karaman, Mersin	Karaman
*Andrena spreta*	Adana, Antalya, Mersin, Osmaniye	Antalya, Hatay*, Mersin
*Andrena symphyti*	Adana, Hatay, Kahramanmaraş, Mersin	Niğde*
*Andrena taedium*	Adana, Muğla, Niğde	Antalya*, Muğla, Niğde
*Andrena thoracica*	Muğla	Niğde*
*Andrena torda*		Muğla*
*Andrena transitoria*	Antalya, Burdur, Hatay, Isparta	Kahramanmaraş*
*Andrena truncatilabris*	Adana, Antalya, Burdur, Hatay, Isparta, Kahramanmaraş, Karaman, Mersin, Muğla	Antalya, Burdur, Mersin
Andrena tscheki ssp. tritica	Karaman	Hatay*
*Andrena variabilis*	Adana	Adana, Mersin*
*Andrena vetula*	Adana, Hatay, Mersin, Osmaniye	Mersin
*Andrena westensis*	Adana, Antalya	Muğla*
*Andrena wolfi*		Hatay*

*New provincial record.

**Table 3.** Number of newly-recorded species by province in the Mediterranean Region of Türkiye.

**Province**	**New Records**
Hatay	17
Muğla	14
Antalya	8
Niğde	8
Mersin	7
Adana	5
Burdur	3
Kahramanmaraş	3
Osmaniye	2
Karaman	1
Denizli	1

**Table 4.** Host plant use and dietary classification for selected *Andrena* species from the Mediterranean Region of Turkey. Much unpublished data will be published in the near future as part of a revision of the *Andrena* of the southern Balkans (Wood 2026).

**Species**	**Host range**	**Literature**
*** Aciandrena ***		
*A. lamiana* Warncke	Probably broadly oligolectic on Brassicaceae	Wood, *unpublished data*
*** Aenandrena ***		
*A. bisulcata* Morawitz	Unclear, strongly associated with Brassicaceae	
*** Brachyandrena ***		
*A. colletiformis* Morawitz	Unclear, probably polylectic with a strong preference for Apiaceae	Wood (2023a); Wood, *unpublished data*
*** Campylogaster ***		
*A. erberi* Morawitz	Unknown	
*** Chlorandrena ***		
*A. astica* Warncke	Broadly oligolectic (Asteraceae: Cichorioideae)	Wood, *unpublished data*; inferred from phylogenetic position
*A. cinereophila* Warncke	Broadly oligolectic (Asteraceae: Cichorioideae)	Wood, *unpublished data*
*A. exquisita* Warncke	Broadly oligolectic (Asteraceae: Cichorioideae)	Wood, *unpublished data*
*A. humabilis* Warncke	Broadly oligolectic (Asteraceae: Cichorioideae)	Wood, *unpublished data*
*A. orientana* Warncke	Broadly oligolectic (Asteraceae: Cichorioideae)	Wood, *unpublished data*
*A. panurgimorpha* Mavromoustakis	Broadly oligolectic (Asteraceae: Cichorioideae)	Wood, *unpublished data*
*** Chrysandrena ***		
*A. fulvago* Christ	Broadly oligolectic (Asteraceae: Cichorioideae)	Westrich (1989)
*A. glandaria* Warncke	Broadly oligolectic (Asteraceae: Cichorioideae)	Inferred from phylogenetic position
*A. hesperia* Smith	Broadly oligolectic (Asteraceae: Cichorioideae)	Wood (2023a)
*** Cordandrena ***		
*A. cordialis* Morawitz	Polylectic	Wood, *unpublished data*
*A. cypria* Pittioni	Probably polylectic	Wood, *unpublished data*
*A. torda* Warncke	Probably polylectic	Wood, *unpublished data*
*** Cryptandrena ***		
*A. brumanensis* Friese	Possibly broadly oligolectic (Fabaceae)	Wood, *unpublished data*
*** Euandrena ***		
*A. bicolor* Fabricius	Polylectic	Westrich (1989)
A. pileate ssp. canigica Warncke	Polylectic	Wood, *unpublished data*
*A. rufula* Schmiedeknecht	Polylectic	Westrich (1989)
*A. symphyti* Schmiedeknecht	Broadly oligolectic (Boraginaceae)	Westrich (1989)
*** Fuscandrena ***		
*A. iliaca* Warncke	Unknown, possibly broadly oligolectic on Brassicaceae	Wood, *unpublished data*
*** Hamandrena ***		
*A. nasuta* Giraud	Narrowly oligolectic on *Anchusa* (Boraginaceae)	Westrich (1989)
*** Holandrena ***		
*A. flavilabris* Schenck	Polylectic	Manderey et al. (2008)
*A. forsterella* Osytshnjuk	Polylectic	Wood, *unpublished data*
*A. labialis* Kirby	Polylectic with a strong preference (Fabaceae)	Wood and Roberts (2017)
*A. variabilis* Smith	Polylectic	Wood, *unpublished data*
*** Leimelissa ***		
*A. fallax* Eversmann	Narrowly oligolectic on *Onobrychis* (Fabaceae)	Wood, *unpublished data*
*** Melanapis ***		
*A. fuscosa* Erichson	Polylectic	Wood (2023a)
*** Melandrena ***		
*A. flavipes* Panzer	Polylectic	Westrich (1989), Wood and Roberts (2017)
*A. hungarica ssp. macroura* Warncke	Unknown, probably polylectic	Inferred from phylogenetic position
*A. limata* Smith	Polylectic	Westrich 1989
*A. magna* Warncke	Unknown, probably polylectic	Inferred from phylogenetic position
*A. nigroaenea* Kirby	Polylectic	Westrich (1989), Wood and Roberts (2017)
*A. nitidemula* Scheuchl & Hazir	Unknown, probably polylectic	Inferred from phylogenetic position
*A. pyropygia* Kreichbaumer	Unknown, probably polylectic	Inferred from phylogenetic position
*A. thoracica* Fabricius	Polylectic	Westrich (1989), Wood and Roberts (2017)
*** Micrandrena ***		
*A. alfkenella* Perkins	Polylectic	Westrich (1989); Wood, *unpublished data*
*A. alfkenelloides* Warncke	Unknown	
*A. alutacea* Stöckhert	Broadly oligolectic on Apiaceae	Schmid-Egger and Scheuchl (1997)
*A. cedricola* Wood		
*A. enslinella* Stöckhert	Possibly broadly oligolectic (Brassicaceae)	Schmid-Egger and Scheuchl (1997)
*A. floricola* Eversmann	Broadly oligolectic (Brassicaceae)	Westrich (1989)
*A. immaculata* Warncke	Unknown	
*A. minutula* Kirby	Polylectic	Westrich (1989)
*A. oedicnema* Warncke	Unknown	
*A. rugothorace* Warncke	Broadly oligolectic (Asteraceae: Asteroideae)	Wood, *unpublished data*
*A. rugulosa* Stöckhert	Polylectic	Westrich (1989)
*A. spreta* Pérez	Polylectic with a strong preference (Brassicaceae)	Wood (2023a)
*** Nobandrena ***		
*A. nobilis* Morawitz	Broadly oligolectic (Brassicaceae)	Schmid-Egger and Scheuchl (1997)
*** Notandrena ***		
*A. aerinifrons* Dours	Broadly oligolectic (Brassicaceae)	Wood (2023a)
*A. purpureomicans* Alfken	Probably broadly oligolectic (Brassicaceae)	Inferred from phylogenetic position
*** Opandrena ***		
*A. schencki* Morawitz	Polylectic	Westrich (1989)
*** Plastandrena ***		
*A. bimaculata* Kirby	Polylectic	Westrich (1989)
*A. pilipes* Fabricius	Polylectic	Westrich (1989)
*** Poecilandrena ***		
*A. crassana* Warncke	Narrowly oligolectic on *Legousia* (Campanulaceae)	Wood, *unpublished data*
*A. laticeps* Morawitz	Unknown	
*** Scitandrena ***		
*A. scita* Eversmann	Polylectic	Wood, *unpublished data*
*** Simrandrena ***		
*A. cinnamonea* Warncke	Unknown	
*A. dorsata* Kirby	Polylectic	Westrich (1989), Wood and Roberts (2017)
*A. gasparella* Patiny	Unknown	
*A. lepida* Schenck	Polylectic	Westrich (1989)
*A. transitoria* Morawitz	Unknown	
*A. vetula* Lepeletier	Broadly oligolectic (Brassicaceae)	Wood (2023a)
*** Taeniandrena ***		
*A. russula* Lepeletier	Broadly oligolectic (Fabaceae)	Westrich (1989)
*A. taedium* Wood	Unknown, but certainly associated with Fabaceae	
*** Truncandrena ***		
*A. combusta* Morawitz	Probably broadly oligolectic (Brassicaceae)	Inferred from phylogenetic position
*A. optata* Warncke	Broadly oligolectic (Brassicaceae)	Wood, *unpublished data*
*A. rufomaculata* Friese	Probably broadly oligolectic (Brassicaceae)	Inferred from phylogenetic position
*A. schmiedeknechti* Magretti	Broadly oligolectic (Brassicaceae)	Wood, *unpublished data*
*A. truncatilabris* Morawitz	Broadly oligolectic (Brassicaceae)	Schmid-Egger and Scheuchl (1997)
A. tscheki ssp. tritica Warncke	Broadly oligolectic (Brassicaceae)	Westrich (1989); Wood, *unpublished data*
*** Ulandrena ***		
*A. abbreviata* Dours	Broadly oligolectic (Asteraceae; Asteroideae)	Wood, *unpublished data*
*A. fulvitarsis* Brullé	Broadly oligolectic (Asteraceae; Asteroideae)	Wood, *unpublished data*
*A. isabellina* Warncke	Broadly oligolectic (Asteraceae; Cichorioideae)	Wood, *unpublished data*
*A. kriechbaumeri* Schmiedeknecht	Broadly oligolectic (Asteraceae; Cichorioideae)	Wood, *unpublished data*
*A. neocypriaca* Mavromoustakis	Broadly oligolectic (Asteraceae; Asteroideae)	Wood, *unpublished data*
*A. westensis* Warncke	Broadly oligolectic (Asteraceae; Cichorioideae)	Wood, *unpublished data*
***incertae sedis***		
*A. discordia* Wood	Unknown	
*A. monacha* Warncke	Unknown	
*A. wolfi* Gusenleitner & Scheuchl	Unknown	
